# Survival estimation of oral cancer using fuzzy deep learning

**DOI:** 10.1186/s12903-024-04279-6

**Published:** 2024-05-02

**Authors:** Rachasak Somyanonthanakul, Kritsasith Warin, Sitthi Chaowchuen, Suthin Jinaporntham, Wararit Panichkitkosolkul, Siriwan Suebnukarn

**Affiliations:** 1 College of Digital Innovation Technology, Rangsit University, Pathum Thani, Thailand; 2 Faculty of Dentistry, Thammasat University, Pathum Thani, Thailand; 3 Udonthani Cancer Hospital, Muang Udonthani, Udonthani, Thailand; 4 Faculty of Dentistry, Khon Kaen University, Khon Kaen, Thailand; 5 Faculty of Science and Technology, Thammasat University, Pathum Thani, Thailand

**Keywords:** Oral cancer, Survival, Artificial intelligence, Deep learning, Fuzzy logic

## Abstract

**Background:**

Oral cancer is a deadly disease and a major cause of morbidity and mortality worldwide. The purpose of this study was to develop a fuzzy deep learning (FDL)-based model to estimate the survival time based on clinicopathologic data of oral cancer.

**Methods:**

Electronic medical records of 581 oral squamous cell carcinoma (OSCC) patients, treated with surgery with or without radiochemotherapy, were collected retrospectively from the Oral and Maxillofacial Surgery Clinic and the Regional Cancer Center from 2011 to 2019. The deep learning (DL) model was trained to classify survival time classes based on clinicopathologic data. Fuzzy logic was integrated into the DL model and trained to create FDL-based models to estimate the survival time classes.

**Results:**

The performance of the models was evaluated on a test dataset. The performance of the DL and FDL models for estimation of survival time achieved an accuracy of 0.74 and 0.97 and an area under the receiver operating characteristic (AUC) curve of 0.84 to 1.00 and 1.00, respectively.

**Conclusions:**

The integration of fuzzy logic into DL models could improve the accuracy to estimate survival time based on clinicopathologic data of oral cancer.

**Supplementary Information:**

The online version contains supplementary material available at 10.1186/s12903-024-04279-6.

## Background

Oral cancer, a major cause of morbidity and mortality worldwide, was estimated at over 370,000 new cases and was the cause of death in over 170,000 cases in 2020 [1]. More than 90% of oral cancers are oral squamous cell carcinomas (OSCCs) which originate from the mucosal epithelium of the oral cavity. Oral cancer is aggressive in its biological behavior, causes extensive destruction of surrounding structures, develops cervical lymph node metastases, and may develop distant metastases over time, even after effective local treatment [2]. The gold standard for diagnosis of oral cancer is tissue biopsy with histopathological assessment [3]. Current therapeutic decision-making in oral cancer depends on tumor staging, which is based on clinical, radiographic, and pathological examination, according to the tumor-node metastasis (TNM) system proposed by the American Joint Committee on Cancer (AJCC). Generally, the treatment of choice for OSCC is surgical resection. Postoperative adjuvant radiotherapy with or without chemotherapy is proposed based on the histopathology of the resection specimen [4]. Oral cancer treatments are primarily costly and critically influence patients in terms of postoperative facial appearance and quality of life. Since the survival rate of oral cancer is directly related to the stage at diagnosis, prevention and early detection efforts have the potential to not only reduce the incidence, but also improve the survival of those who develop this disease [5]. With advances in computational analytical research, the application of deep learning could be used to analyze and predict the survivability and associated variables of oral cancer. Survival estimation is valuable to both physicians and patients in determining cancer outcomes, contributing to appropriate treatment planning, and avoiding unnecessary therapies.

Deep learning (DL), a subfield of artificial intelligence (AI), is known to be a human-inspired brain neural network. DL has been demonstrated to make highly accurate predictions from large amounts of high feature dimensional data by representing complex relationships using multilevel structures [6]. In the medical field, DL has been proven to have efficient information processing and has been widely used in medical specialties including pathology, radiology, oncology, surgery, etc. In recent years, various studies have been conducted on the application of DL to the analysis of oral cancer data, including imaging and clinicopathological data, with the aim of increasing the survival rate of patients [7–9]. For the AI-based survivability prediction of oral cancer, there are various studies, which implemented machine learning (ML) and DL algorithms in prediction of oral cancer prognosis and survival rate, including random survival forests, logistic regression, Support Vector Machine (SVM), K-nearest neighbors, Naïve Bayes, and DL models [9–16].

Nevertheless, the characteristic of medical data (including cancer data) is unpredictable uncertainty, which is a challenge in the medical field and affects the DL model by not knowing the optimized architecture and parameters to predict future data. Data uncertainty arises from several sources including missing information, noise, and bias [17]. To overcome data uncertainties, the fuzzy logic system has been integrated with DL, called fuzzy deep learning (FDL), to solve classification problems. The fuzzy system automatically learns the fuzzy membership function to extract fuzzy rules from the training data. Fuzzy logic provides a dynamic, probable, real-time, intensive rule base for the system [18]. In recent years, FDL has been successfully applied and improved the classification and mortality prediction of various cancer data, such as melanoma diagnosis from clinical skin image data [19], detection of the indirect immunofluorescence pattern associated with nasopharyngeal cancer from pathological data [20], and prediction of breast cancer mortality using genomic data [18]. To predict cancer survivability, ‘time to event’, which is the time from cancer diagnosis to survival or death at a specific point of time, should be incorporated into the analysis of cancer data. Therefore, the application of FDL to clinicopathologic and survival time data of oral cancer might be able to address data uncertainty and achieve more precise mortality predictions for oral cancer patients.

The aims of this study are to develop and evaluate an FDL-based survival time estimation model that utilizes clinicopathological data to predict the survival time of oral cancer patients. This work is expected to be a medical decision support model for clinicians to establish the most appropriate treatment planning and predict the treatment outcome of oral cancer for the new era of medical practice.

## Methods

**Fig. 1:**
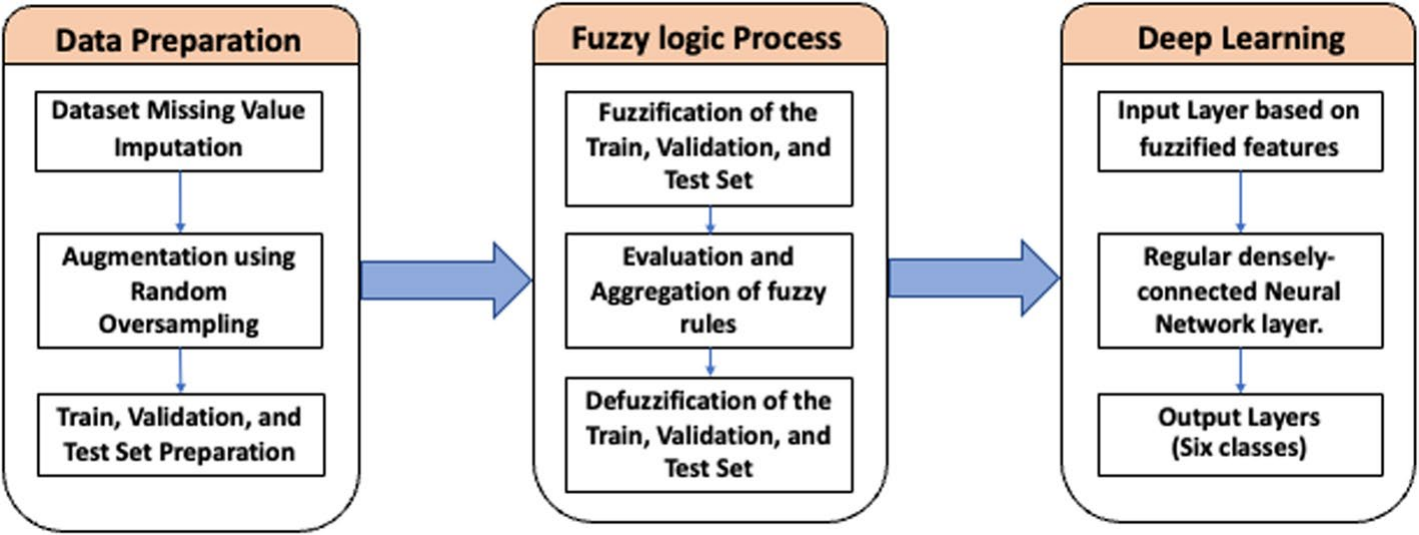
The methodology workflow

This work was divided into two experiments by deploying the DL-based multiclass classification architecture and integrating the fuzzy logic system to develop the FDL model for survival estimation of oral cancer. This work was performed under the guidelines of the Transparent reporting of a multivariable prediction model for individual prognosis or diagnosis (TRIPOD), the reporting guideline for diagnostic and prognostic prediction studies [21]. The methodologic workflow is illustrated in Fig. 1.

### Data acquisition

Electronic medical records of 891 OSCC patients, treated with surgery, with or without radiochemotherapy, between January 2011 and December 2019 from the Oral and Maxillofacial Surgery Clinic and the Regional Cancer Center, were included in this study. Inclusion criteria were: 1) histologically confirmed primary OSCC treated with surgery with or without radiochemotherapy, 2) post-operative follow-up of at least 5 years, and 3) availability of relevant clinicopathologic variables. Exclusion criteria were preoperative patients with metastatic disease, secondary primary cancer, perioperative mortality, history of radiotherapy and/or chemotherapy, or previous history of head and neck cancer. According to the exclusion criteria, 581 patient records were suitable for analysis and DL model development. The variables used for developing the DL models included clinical characteristics, pathologic findings after surgery, primary treatment, postoperative locoregional recurrence, distant metastases, and survival time. The overall survival time variable was categorized into six classes based on the range of survival time from 0-12 months to more than 60 months for the DL model’s learning process. The missing data values were resolved through imputation. In addition, the class imbalance data was augmented with the oversampling technique. To develop the DL model, the dataset was randomly split and assigned as the training (70%), validation (10%), and test datasets (20%), respectively. Data splitting technique is crucial in DL as it helps to prevent overfitting, reduce dataset bias, and enhance model performance [22]. The dataset analyzed during the current study is not publicly available but is available from the corresponding author upon reasonable request.

### Deep learning model

The basic architecture of a DL consists of three components: input layers, feature-extraction layer, and classification layer. In this study, the sequential model with densely connected neural network layer was selected to create DL-based classification models for estimation of the overall survival time of oral cancer patients based on clinicopathologic data of oral cancer. The sequential model, which is a linear stack of layers where each layer has exactly one input tensor and one output tensor, is a class of machine learning models designed for tasks that involve sequential data, including textual data, time series data or any other ordered data [23].

### Fuzzy logic systems

Fuzzy logic aims to perform representations in nonlinear ways just as human logic does. Linguistic terms are used that differ from conventional logic, which is usually binary, and fuzzy logic allows gradual representations in a continuous space, thereby allowing levels of uncertainty [24, 25]. Fuzzy sets allow the use of membership, meaning their elements are capable of being part of one or more classes at the same time. The range of these sets is defined as human logic, where they depend on the concept or user applying them.

The Fuzzy set $A$ is from Universe $X$ that goes from $[\mathrm{0,1}]$, belonging to a continuous function, i.e., ${\mu }_{A}:X\to [\mathrm{0,1}]$. The membership function $A$ is denoted as ${\mu }_{A}(x)$; this function is defined in Eq. (1).

1 $$A=\left\{\left(x,{\mu }_{A}\left(x\right)\right)|x\in X\right\}$$

The different membership functions (MFs) most used to represent a fuzzy set are the following: the triangular MF, the trapezoidal MF, and the Gaussian MF, which is used in the fuzzy edge-detection approach presented in this paper. The Gaussian MF consists of two parameters $\left\{c,\sigma \right\}$ and is expressed in Eq. (2); variable $c$ represents the mean of the MF and $\sigma$ the variation.

2 $$gaussian\left(x;c,\sigma \right)={\text{exp}}\left(-\frac{1}{2}\frac{{(x-c)}^{2}}{{\sigma }^{2}}\right)$$

### Fuzzy deep learning

**Fig. 2:**
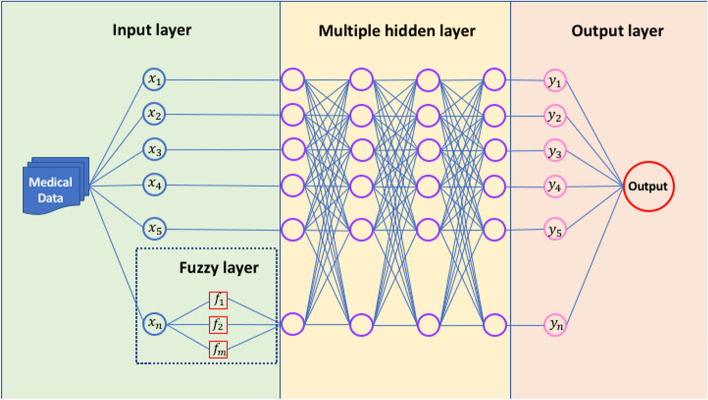
The process of fuzzy deep learning model

DLs offer a new approach for researchers to explore the various effective ways to overcome the "black-box" issue. One of the promising solutions found by researchers is to incorporate the concepts of the explainable rule-based structure called fuzzy inference with DL. Fuzzy systems can be used as an integral part of DL models by using fuzzy parameters or by using fuzzy logic for selecting training parameters (Fig. 2.). The FDL is divided into three main components:

(i) Input Membership Function layer (fuzzification and defuzzification part)
  In this step, the finetuned data after preprocessing has been applied into the model where every input ${x}_{i}$ in this layer is an adaptive MF to generate the membership degree of linguistic variables. This stage helps to convert discrete values into the fuzzy membership degree.
(ii) Deep learning part
  This is the most essential stage in terms of processing the data with a huge number of input features for some high-level abstraction in terms of DL. The output coming from the previous stage is used in this layer to initialize the DL. Since the proposed approach transmits the information in two ways, therefore it feed-forward the fuzzy input signals to the hidden layer. The nodes in the hidden layers are interconnected to each other in a way that the information flows in a forward pass until the next layer. The input information processed via a single neuron is presented in Fig. 2.
(iii) Output layer
  From this stage, the DL drives the defuzzification block. The features extracted and learned from the data via DNN will be further processed to defuzzifier and generates the output of the proposed model depending on whether or not the fuzzy if-then rules exists in the network. A benefit to this system is that these fuzzy rules are used to explain the behavior of the fuzzy system. It is to be noted, however, that expert knowledge is not required, as the network will be set up to automatically extract fuzzy rules from the numerical data. Parameters in this layer are linear parameters that are tuned during the training process of the model. After the process of defuzzification, the next layer is the last layer in which the output of the whole network is calculated as follows:

3 $${x}^{*}=\frac{\sum_{i=1}^{N}{\overline{w} }_{i}{f}_{i}}{\sum_{i=1}^{N}{w}_{i}}$$

Here,${x}^{*}$ is defuzzified and ${f}_{i}$ represents the firing area of ${i}^{th}$ rules and $N$ is the total number of rules fired and ${\overline{w} }_{i}$ represents the center of area. This layer simply sums up the outputs of all rules in the previous layer and converts fuzzy values into discrete output. Afterward, the learning algorithm back-propagates the error to update the weights and parameters of the model until the predicted output is similar to the desired output for efficient classification of the data. The weights of the nodes are updated using a gradient-based optimization algorithm [24–26] which combines between Stochastic Gradient Descent (SGD) with backpropagation.

The FDL approach enables the extraction of fuzzy rules with the involvement of a human expert to solve the "black-box" problem of DL, which achieves better accuracy than a DL with the same level of abstraction [26].

### Experiment

This work developed FDL**-** and DL- based survival time estimation models and compared the survival prediction performance with machine learning (ML) models, including support vector machines (SVM), random forest (RF).

### FDL- and DL- based survival time estimation models

The FDL model was then developed by combining the fuzzy logic and deep neural network into a single architecture. The proposed models were implemented using a Tesla P100 (Nvidia Corporation, CA, USA), Nvidia driver: 460.32 (Nvidia Corporation, CA, USA) and CUDA: 11.2 (Nvidia Corporation, CA, USA). TensorFlow and Keras packages were implemented for core layers regular densely-connected neural network layer model. FDL were initialized with a learning rate of 0.00001 and batch size of 32. Training was stopped after 12 epochs passed without improvement in validation accuracy and there was no significant indication of over-fitting.

### ML-based survival estimation models

Two traditional ML models, including SVM and RF, were adopted to create the ML-based survival estimation models with the training dataset for comparison to FDL-based survival time estimation model. The SVM is an ML that aims to find a hyperplane to maximize the margin between classes by mapping and transforming the instance space. SVM cast survival analysis as a classification problem with an ordinal target variable [27, 28]. RF is an ML that is most frequently used to solve problems related to classification and regression by building ensembles from decision trees and combining the results to give a final decision. RF extends random forest to censored data over the entire lifetime [29].

The proposed models were implemented using a Tesla P100 (Nvidia Corporation, CA, USA), Nvidia driver: 460.32 (Nvidia Corporation, CA, USA) and CUDA: 11.2 (Nvidia Corporation, CA, USA). The model is developed using TensorFlow and scikit-learn package of Python programming. The hyperparameter of SVM tuning uses grid search and cross validation and training SVM model using radial kernel. For the hyperparameter of RF, we have defined 10 trees in our random forest and the loss function, we have used entropy to measure the quality of the split.

### Model evaluation and statistical analysis

The statistical analysis was performed using the R programming language (R Core Team, Vienna, Austria, 2018). The estimation performance of models was evaluated by precision, recall, F1 score, sensitivity, specificity, and accuracy. Receiver operating characteristic (ROC) was generated using a Python script. The ROC curve plotted by varying the operating threshold was used to assess the ability of the model to distinguish each class. An area under the ROC curve (AUC) was used to summarize the accuracy for model classification.

## Results

The clinicopathologic characteristics of the 581 patients in this study were as follows: 78 patients were at stage I; 109 patients were at stage II; 106 patients were at stage III; 279 patients were stage IVa; and 9 patients were at stage IVb according to the 7^th^ edition of American Joint Committee on Cancer (AJCC) staging [4]. After a 5-year follow-up after treatment, 78 patients had local recurrence, 35 patients had distant metastases, and 279 patients had died. For overall survival time of oral cancer patients, 279 patients died in 60 months, and 302 patients survived more than 60 months. The clinicopathologic, primary treatment, postoperative recurrence, distant metastasis, and survival time variables of the dataset, which were divided into six classes according to the survival time, are shown in Table 1.

**Table 1:** Summary of clinicopathologic, primary treatment, postoperative recurrence, distant metastasis, and survival time variables of the overall dataset (*n*=581).

Variables		Number of patients (%)
Sex	Male	278 (47.8%)
	Female	303 (52.2%)
Age	< 41 years	41 (7.1%)
	41 – 60 years	241 (41.4%)
	>60 years	299 (51.5 %)
Tumor location	Oral tongue	258 (44.4%)
	Floor of mouth	53 (9.1%)
	Buccal mucosa	64 (11.0%)
	Alveolar ridge	110 (18.9%)
	Hard palate	13 (2.2%)
	Lip	67 (11.6%)
	Retromolar trigone	16 (2.8%)
T stage	T1	98 (16.9%)
	T2	194 (33.4%)
	T3	100 (17.2%)
	T4a	186 (32.0%)
	T4b	3 (0.5%)
pN stage	N0	298 (51.3%)
	N1	107 (18.4%)
	N2a	25 (4.3%)
	N2b	108 (18.6%)
	N2c	38 (6.5%)
	N3	5 (0.9%)
TNM stage	I	78 (13.4%)
	II	109 (18.8%)
	III	106 (18.2%)
	IVa	279 (48.0%)
	IVb	9 (1.6%)
Histologic grade	Well	387 (66.6%)
	Moderate	165 (28.4%)
	Poor	29 (5.0%)
Lymph node metastasis	Positive	283 (48.7%)
	Negative	298 (51.3%)
Lymphovascular invasion	Positive	100 (17.2%)
	Negative	481 (82.8%)
Perineural invasion	Positive	138 (23.8%)
	Negative	443 (76.2%)
Margin	Positive	114 (19.6%)
	Close	20 (3.4%)
	Clear	447 (77.0%)
Extranodal extension	Positive	44 (7.6%)
	Negative	537 (92.4%)
Primary treatment	Surgery only	150 (25.8%)
	Surgery combines with radiotherapy	302 (52.0%)
	Surgery combines with concurrence chemoradiotherapy	129 (22.2%)
Locoregional recurrence	Local recurrence	78 (13.4%)
	No recurrence	503 (86.6%)
Post operative distant metastasis	Lung metastasis	29 (5.0%)
	Bone metastasis	6 (1.0%)
	No metastasis	546 (94.0%)
Overall survival time (Class)	0 – 12 months (Class 0)	154 (26.5%)
	13 – 24 months (Class 1)	67 (11.5%)
	25 – 36 months (Class 2)	33 (5.7%)
	37 – 48 months (Class 3)	19 (3.3%)
	49 – 60 months (Class 4)	6 (1.0%)
	> 60 months (Class 5)	302 (52.0%)

### DL-based survival time estimation model

**Fig. 3:**
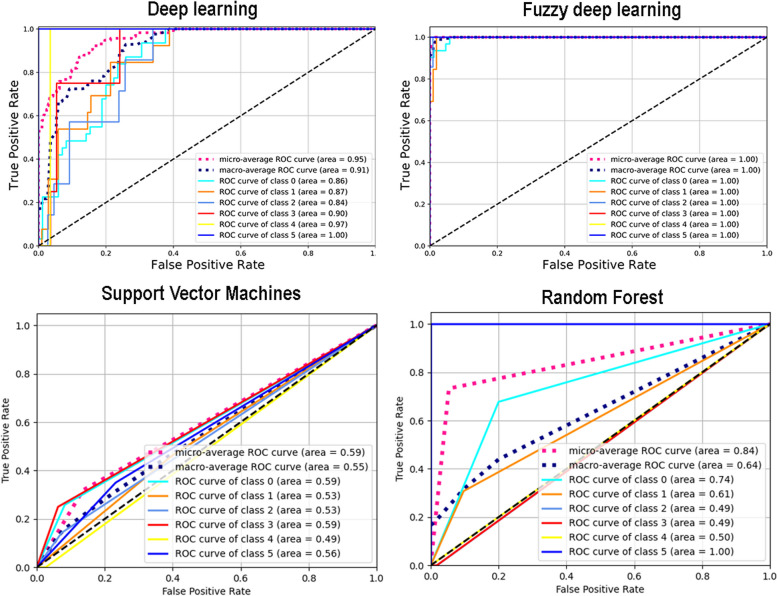
The receiver operating characteristic (ROC) curves for multiclass classification of DL, FDL, SVM and RF in survival time estimation. The AUC of DL, FDL, SVM and RF were 0.84 to 1.00, 1.00, 0.49 to 0.59, and 0.49 to 1.00 for classification of survival time classes, respectively. AUC, area under the ROC curve; DL, deep learning; FDL, fuzzy deep learning; SVM, Support Vector Machines; RF, Random Forest

The DL model’s performance for oral cancer survival time estimation based on clinicopathologic data is reported in Table 2. The DL-based survival time estimation model achieved a precision of 0.00 to 0.97, a recall (sensitivity) of 0.00 to 1.00, an F1 score of 0.00 to 0.98, and a specificity of 0.82 to 1.00 for estimating oral cancer survival time. In addition, the overall accuracy of the model was 0.74. The AUC ranged from 0.84 to 1.00, indicating excellent agreement. The ROC curves of the model are shown in Fig. 3.

**Table 2:** Multiclass classification performances of deep learning in estimation of oral cancer survival time

Class	Precision	Recall (Sensitivity)	F1-score	Accuracy	Specificity	AUC of ROC curve
Class 0: 0 – 12 months	0.74	0.45	0.56	0.81	0.82	0.86
Class 1: 13 – 24 months	0.38	0.62	0.47	0.84	0.95	0.87
Class 2: 25 – 36 months	0.29	0.29	0.29	0.91	0.95	0.84
Class 3: 37 – 48 months	0.29	0.50	0.36	0.94	0.98	0.90
Class 4: 49 – 60 months	0.00	0.00	0.00	0.99	0.99	0.97
Class 5: > 60 months	0.97	1.00	0.98	0.98	1.00	1.00
				**Overall accuracy = 0.74**		

*AUC* Area under the receiver operating characteristic (ROC) curve

### FDL-based survival time estimation model

The FDL model’s performance for oral cancer survival time estimation based on clinicopathologic data is reported in Table 3. The FDL-based survival time estimation model achieved a precision of 0.8 to 1.00, a recall (sensitivity) of 0.9 to 1.00, an F1 score of 0.89 to 1.00, and a specificity of 0.97 to 1.00 for estimating oral cancer survival time. In addition, the overall accuracy of the model was 0.97. The AUC of all classes was 1.00, indicating excellent agreement. The ROC curves of the model are shown in Fig. 3.

**Table 3:** Multiclass classification performances of Fuzzy deep learning in estimation of oral cancer survival time

Class	Precision	Recall (Sensitivity)	F1-score	Accuracy	Specificity	AUC of ROC curve
Class 0: 0 – 12 months	1.00	0.90	0.95	0.97	0.97	1.00
Class 1: 13 – 24 months	0.86	0.92	0.89	0.97	0.99	1.00
Class 2: 25 – 36 months	0.88	1.00	0.93	0.99	1.00	1.00
Class 3: 37 – 48 months	0.80	1.00	0.89	0.99	1.00	1.00
Class 4: 49 – 60 months	1.00	1.00	1.00	1.00	1.00	1.00
Class 5: > 60 months	1.00	1.00	1.00	1.00	1.00	1.00
				**Overall accuracy = 0.97**		

*AUC* Area under the receiver operating characteristic (ROC) curve

### ML-based survival estimation models

The performance of SVM and RF models for oral cancer survival time estimation based on clinicopathologic data is shown in Table 4. The SVM and RF models achieved a precision of 0.00 to 0.62, and 0.00 to 1.00, a recall (sensitivity) of 0.00 to 0.46, and 0.00 to 1.00, an F1 score of 0.00 to 0.45, and 0.00 to 1.00, and a specificity of 0.52 to 0.99, and 0.87 to 1.00 for estimating oral cancer survival time, respectively. In addition, the overall accuracy of the SVM and RF models was 0.90 and 0.91, respectively. The AUC of SVM and RF ranged from 0.49 to 0.59 and 0.49 to 1.00, respectively. The ROC curves of the model are shown in Fig. 3.

**Table 4:** Multiclass classification performances of machine learning models in estimation of oral cancer survival time

Models	Class	Precision	Recall (Sensitivity)	F1-score	Accuracy	Specificity	AUC of ROC curve
Support Vector Machines	Class 0: 0 – 12 months	0.53	0.26	0.35	0.74	0.77	0.59
	Class 1: 13 – 24 months	0.13	0.46	0.20	0.59	0.90	0.53
	Class 2: 25 – 36 months	0.11	0.14	0.13	0.88	0.94	0.53
	Class 3: 37 – 48 months	0.13	0.25	0.17	0.91	0.97	0.59
	Class 4: 49 – 60 months	0.00	0.00	0.00	0.97	0.99	0.49
	Class 5: > 60 months	0.62	0.35	0.45	0.55	0.52	0.56
					**Overall accuracy = 0.90**		
Random Forest	Class 0: 0 – 12 months	0.55	0.68	0.61	0.77	0.87	0.74
	Class 1: 13 – 24 months	0.29	0.31	0.30	0.84	0.91	0.61
	Class 2: 25 – 36 months	0.00	0.00	0.00	0.92	0.94	0.49
	Class 3: 37 – 48 months	0.00	0.00	0.00	0.95	0.96	0.49
	Class 4: 49 – 60 months	0.00	0.00	0.00	0.99	0.99	0.50
	Class 5: > 60 months	1.00	1.00	1.00	1.00	1.00	1.00
					**Overall accuracy = 0.91**		

*AUC* Area under the receiver operating characteristic (ROC) curve

## Discussion

This article proposes an FDL-based survival time estimation model based on clinicopathologic variables of oral cancer with the ability to estimate survival time within 5 years after surgical treatment of oral cancer patients. Oral cancer mortality may vary in each patient, even at the same TNM stage and treatment, due to the uncertain nature of the cancer data, which affected the ability of the AI or DL model to correctly predict or estimate the survival time of cancer patients. The application of a fuzzy system to DL can tackle such uncertain and imprecise information through the process of fuzzification. Numerical input was converted to fuzzy sets or linguistic statements with some degree of membership. The broad framework provided by synthesizing control rules based on real medical data has been shown to be effective in improving the performance rate and accuracy of model prediction of uncertainty data [30].

This study demonstrated that integrating the fuzzy logic with the DL model yielded better optimized capability, which achieved an overall accuracy of 0.97 and an AUC of 1.00. The performance was higher than the original DL model, which achieved an overall accuracy of 0.74 and an AUC of 0.84 to 1.00, for oral cancer survival time estimation. In addition, the performance of FDL model also achieved higher accuracy than the traditional ML models, including SVM and RF, which achieved an overall accuracy of 0.90 and 0.91, for oral cancer survival time estimation, respectively. Improved accuracy of FDL indicated that the fuzzy logic affected model performance by increasing flexibility and improving classification performance in the analysis of clinicopathologic variables to estimate survival time of oral cancer patients. A previous study on the application of the fuzzy system to analyze cancer data found that the fuzzy system could improve the accuracy of DL models for the prediction of breast cancer mortality with an accuracy of 0.87 [18]. In oral cancer, clinicopathologic factors; including TNM staging, adverse pathologic features, and tumor recurrence; were related to oral cancer prognosis that affected patient survival time [31, 32]. A patient with oral cancer dying within a short interval after diagnosis is a more hazardous situation than that of a patient dying after a long survival interval. Therefore, a more accurate survival estimation model can offer several advantages, including extracting the meaningful information from clinicopathological data in oral cancer, connecting the significance of features related to the oral cancer patient outcomes, and providing precise survival estimation results for cancer mortality using clinicopathological data.

In the real-world scenario, the cancer patient might want to know their prognosis and even survival time after cancer treatment. Traditional survival analysis methods, including the Cox proportional hazards regression model, have been used to estimate survival outcomes for individuals and have generally focused on differences in patient cohorts [33]. These models make linearity assumptions and therefore cannot model nonlinear relationships that may be present in a real-world setting, reflecting the complexity of biomedicine data. Unlike traditional survival analysis methods, DL models exhibit improved fit for variables with a nonlinear relationship, which can automatically survival risks using nonlinear risk functions and to predict individual survival outcomes from learned representations [34, 35]. However, the characteristic of oral cancer data is not only nonlinear relationships, but also unpredictable uncertainty. To deal with this uncertain nature of oral cancer data, this study integrated the fuzzy logic system with DL to create the FDL model and found that the FDL model achieved higher accuracy in estimating survival time of oral cancer than the DL model. The results demonstrated that the fuzzy logic system had an advantage in dealing with the uncertain nature of oral cancer data. Therefore, this FDL-based data analysis model could be used to benefit oral cancer physicians, including oral and maxillofacial surgeons, otolaryngologists, and oncologists. This clinical decision-making model based on clinicopathologic data could be used in treatment planning and prognosis prediction to avoid unnecessary treatment for oral cancer patients. Moreover, this model could also be beneficial for cancer patients to plan and manage their private affairs and family issues from the survival time and disease prognosis.

This study has limitations that need to be addressed. First, the limited amount of data and the class imbalance of clinicopathologic variables, especially TNM staging, which is a common problem of DL research in the medical field [36]. The cancer data used in this study included only surgical cases and were obtained from only two cancer centers. Second, the variables analyzed in this study were only clinical and pathological factors from surgical cases of oral cancer. The future direction of this study is to establish a larger dataset of oral cancer from multi-cancer centers with other treatment modalities data, including treatment with radiotherapy only and/or chemotherapy or palliative treatment of oral cancer patients. In addition, we will consider integrating the fuzzy logic system to Transformer, which was based solely on attention mechanisms, dispensing with recurrence and convolutions entirely [37], to develop an AI-based medical model for oral cancer data analysis. This is expected to make the model more robust and reliable for implementation in the real-world situation. Moreover, the DL-based survival prediction of oral cancer should consider genomic data as another key parameter to analyze and predict the survival rate of oral cancer patients.

## Conclusions

The integration of fuzzy logic and DL into a single architecture could improve the performance of the multiclass classification model to classify and estimate oral cancer survival time with promising results. This model is expected to provide an AI-based oral cancer survival time estimation based on clinicopathologic data as supplementary information for clinicians to select the most appropriate treatment planning for the oral cancer patient.

### Supplementary Information

**Supplementary Material 1.** 

**Supplementary Material 2.** 

**Supplementary Material 3.** 

## Data Availability

The data of this study is available from the corresponding author upon reasonable request.

## Abbreviations

DL: Deep learning
FDL: Fuzzy deep learning
OSCC: Oral squamous cell carcinoma
SVM: Support vector machines
RF: Random forest
AUC: Area under the receiver operating characteristic curve
