# Supplementary material for: Survival estimation of oral cancer using fuzzy deep learning
Source: BMC Oral Health. 2024 May 2;24:519. doi: 10.1186/s12903-024-04279-6 (PMC11067185; doi:10.1186/s12903-024-04279-6)
Supplement: Supplementary file 1 — Supplementary Material 1. [file 12903_2024_4279_MOESM1_ESM.pdf]

FuzzyDeepLearningOralCancer_FDL

April 17, 2024

[1]:
```
# for reading data
import pandas as pd
import numpy as np
from sklearn.preprocessing import LabelEncoder
from keras.wrappers.scikit_learn import KerasClassifier
from keras.utils import np_utils

# for modeling
from keras.models import Sequential
from keras.layers import Dense, Dropout
from keras.callbacks import EarlyStopping
from tensorflow.keras import optimizers
```

[2]:
```
train_ds = pd.read_csv('8.Training_oral_cancer.csv')
df_train_ds = train_ds.sample(frac=1).reset_index(drop=True)

validate_ds = pd.read_csv('4.validate_oral_cancer.csv')
df_validate_ds = validate_ds.sample(frac=1).reset_index(drop=True)


test_ds = pd.read_csv('5.test_oral_cancer.csv')
df_test_ds = test_ds.sample(frac=1).reset_index(drop=True)
```

[3]:
```
############# Training  split into X and Y
Y_train = df_train_ds['target']
X_train = df_train_ds.drop(['target'], axis=1)
print("Training Set")
print(X_train.shape)
print(Y_train.shape)

# convert to numpy arrays
X_train = np.array(X_train)


############# Validate split into X and Y
Y_validate = df_validate_ds['target']
X_validate = df_validate_ds.drop(['target'], axis=1)
print("Validation Set")
```

```python
print(X_validate.shape)
print(Y_validate.shape)

# convert to numpy arrays
X_validate = np.array(X_validate)


############ Test split into X and Y
Y_test = df_test_ds['target']
X_test = df_test_ds.drop(['target'], axis=1)
print("Test Set")
print(X_test.shape)
print(Y_test.shape)

# convert to numpy arrays
X_test = np.array(X_test)
```

```
Training Set
(1812, 13)
(1812,)
Validation Set
(58, 13)
(58,)
Test Set
(116, 13)
(116,)
```

[4]:

```python
############################ Show Y Training
print("############################ Y Training")

Y_train.head()

## 0        setosa
## 1     virginica
## 2    versicolor
## 3     virginica
## 4        setosa

# work with labels
# encode class values as integers
encoder = LabelEncoder()
encoder.fit(Y_train)
encoded_Y_train = encoder.transform(Y_train)
# convert integers to dummy variables (i.e. one hot encoded)
dummy_y_train = np_utils.to_categorical(encoded_Y_train)

print(encoded_Y_train)
```

```python
############################# Show Y Validate
print("############################# Y Validation")
Y_validate.head()
encoder = LabelEncoder()
encoder.fit(Y_validate)
encoded_Y_validate = encoder.transform(Y_validate)
dummy_y_validate = np_utils.to_categorical(encoded_Y_validate)
print(encoded_Y_validate)
print(dummy_y_validate)


############################# Show Y Test
print("############################# Y Validation")
Y_test.head()
encoder = LabelEncoder()
encoder.fit(Y_test)
encoded_Y_test = encoder.transform(Y_test)
dummy_y_test = np_utils.to_categorical(encoded_Y_test)
print(encoded_Y_test)
print(dummy_y_test)
```

```
############################# Y Training
[3 5 5 … 2 2 2]
[[0. 0. 0. 1. 0. 0.]
 [0. 0. 0. 0. 0. 1.]
 [0. 0. 0. 0. 0. 1.]
 …
 [0. 0. 1. 0. 0. 0.]
 [0. 0. 1. 0. 0. 0.]
 [0. 0. 1. 0. 0. 0.]]
############################# Y Validation
[1 5 3 5 5 0 5 5 5 5 5 1 0 2 4 2 5 5 0 0 5 0 0 0 0 5 5 1 5 0 5 5 5 0 5 5 1 5
 5 5 5 1 5 5 5 5 2 5 0 1 5 0 5 3 0 0 0 5 1]
[[0. 1. 0. 0. 0. 0.]
 [0. 0. 0. 0. 0. 1.]
 [0. 0. 0. 1. 0. 0.]
 [0. 0. 0. 0. 0. 1.]
 [0. 0. 0. 0. 0. 1.]
 [1. 0. 0. 0. 0. 0.]
 [0. 0. 0. 0. 0. 1.]
 [0. 0. 0. 0. 0. 1.]
 [0. 0. 0. 0. 0. 1.]
 [0. 0. 0. 0. 0. 1.]
 [0. 1. 0. 0. 0. 0.]
 [1. 0. 0. 0. 0. 0.]
 [0. 0. 1. 0. 0. 0.]
```

```
 [0. 0. 0. 0. 1. 0.]
 [0. 0. 1. 0. 0. 0.]
 [0. 0. 0. 0. 0. 1.]
 [0. 0. 0. 0. 0. 1.]
 [1. 0. 0. 0. 0. 0.]
 [1. 0. 0. 0. 0. 0.]
 [0. 0. 0. 0. 0. 1.]
 [1. 0. 0. 0. 0. 0.]
 [1. 0. 0. 0. 0. 0.]
 [1. 0. 0. 0. 0. 0.]
 [1. 0. 0. 0. 0. 0.]
 [0. 0. 0. 0. 0. 1.]
 [0. 0. 0. 0. 0. 1.]
 [0. 1. 0. 0. 0. 0.]
 [0. 0. 0. 0. 0. 1.]
 [1. 0. 0. 0. 0. 0.]
 [0. 0. 0. 0. 0. 1.]
 [0. 0. 0. 0. 0. 1.]
 [0. 0. 0. 0. 0. 1.]
 [1. 0. 0. 0. 0. 0.]
 [0. 0. 0. 0. 0. 1.]
 [0. 0. 0. 0. 0. 1.]
 [0. 1. 0. 0. 0. 0.]
 [0. 0. 0. 0. 0. 1.]
 [0. 0. 0. 0. 0. 1.]
 [0. 0. 0. 0. 0. 1.]
 [0. 0. 0. 0. 0. 1.]
 [0. 1. 0. 0. 0. 0.]
 [0. 0. 0. 0. 0. 1.]
 [0. 0. 0. 0. 0. 1.]
 [0. 0. 0. 0. 0. 1.]
 [0. 0. 0. 0. 0. 1.]
 [0. 0. 1. 0. 0. 0.]
 [0. 0. 0. 0. 0. 1.]
 [1. 0. 0. 0. 0. 0.]
 [0. 1. 0. 0. 0. 0.]
 [0. 0. 0. 0. 0. 1.]
 [1. 0. 0. 0. 0. 0.]
 [0. 0. 0. 0. 0. 1.]
 [0. 0. 0. 1. 0. 0.]
 [1. 0. 0. 0. 0. 0.]
 [1. 0. 0. 0. 0. 0.]
 [1. 0. 0. 0. 0. 0.]
 [0. 0. 0. 0. 0. 1.]
 [0. 1. 0. 0. 0. 0.]]
############################ Y Validation
[3 4 5 1 3 5 1 5 1 1 1 5 2 5 0 5 5 5 5 5 5 5 5 0 1 1 5 0 5 0 5 5 0 5 0 5 0
 3 0 5 5 5 0 5 0 2 5 5 0 5 5 0 0 1 5 0 5 2 0 0 0 0 5 5 5 5 2 0 5 0 0 1 0 1
```

```
 0 1 5 5 0 5 5 1 5 5 0 0 5 5 5 0 0 5 5 5 5 0 5 5 5 5 1 2 5 5 5 0 5 3 5 5 5
 5 2 5 2 0]
[[0. 0. 0. 1. 0. 0.]
 [0. 0. 0. 0. 1. 0.]
 [0. 0. 0. 0. 0. 1.]
 [0. 1. 0. 0. 0. 0.]
 [0. 0. 0. 1. 0. 0.]
 [0. 0. 0. 0. 0. 1.]
 [0. 1. 0. 0. 0. 0.]
 [0. 0. 0. 0. 0. 1.]
 [0. 1. 0. 0. 0. 0.]
 [0. 1. 0. 0. 0. 0.]
 [0. 1. 0. 0. 0. 0.]
 [0. 0. 0. 0. 0. 1.]
 [0. 0. 1. 0. 0. 0.]
 [0. 0. 0. 0. 0. 1.]
 [1. 0. 0. 0. 0. 0.]
 [0. 0. 0. 0. 0. 1.]
 [0. 0. 0. 0. 0. 1.]
 [0. 0. 0. 0. 0. 1.]
 [0. 0. 0. 0. 0. 1.]
 [0. 0. 0. 0. 0. 1.]
 [0. 0. 0. 0. 0. 1.]
 [0. 0. 0. 0. 0. 1.]
 [0. 0. 0. 0. 0. 1.]
 [1. 0. 0. 0. 0. 0.]
 [0. 1. 0. 0. 0. 0.]
 [0. 1. 0. 0. 0. 0.]
 [0. 0. 0. 0. 0. 1.]
 [1. 0. 0. 0. 0. 0.]
 [0. 0. 0. 0. 0. 1.]
 [1. 0. 0. 0. 0. 0.]
 [0. 0. 0. 0. 0. 1.]
 [0. 0. 0. 0. 0. 1.]
 [1. 0. 0. 0. 0. 0.]
 [0. 0. 0. 0. 0. 1.]
 [1. 0. 0. 0. 0. 0.]
 [0. 0. 0. 0. 0. 1.]
 [1. 0. 0. 0. 0. 0.]
 [0. 0. 0. 1. 0. 0.]
 [1. 0. 0. 0. 0. 0.]
 [0. 0. 0. 0. 0. 1.]
 [0. 0. 0. 0. 0. 1.]
 [0. 0. 0. 0. 0. 1.]
 [1. 0. 0. 0. 0. 0.]
 [0. 0. 0. 0. 0. 1.]
 [1. 0. 0. 0. 0. 0.]
 [0. 0. 1. 0. 0. 0.]
```

```
[0. 0. 0. 0. 0. 1.]
[0. 0. 0. 0. 0. 1.]
[1. 0. 0. 0. 0. 0.]
[0. 0. 0. 0. 0. 1.]
[0. 0. 0. 0. 0. 1.]
[1. 0. 0. 0. 0. 0.]
[1. 0. 0. 0. 0. 0.]
[0. 1. 0. 0. 0. 0.]
[0. 0. 0. 0. 0. 1.]
[1. 0. 0. 0. 0. 0.]
[0. 0. 0. 0. 0. 1.]
[0. 0. 1. 0. 0. 0.]
[1. 0. 0. 0. 0. 0.]
[1. 0. 0. 0. 0. 0.]
[1. 0. 0. 0. 0. 0.]
[1. 0. 0. 0. 0. 0.]
[0. 0. 0. 0. 0. 1.]
[0. 0. 0. 0. 0. 1.]
[0. 0. 0. 0. 0. 1.]
[0. 0. 0. 0. 0. 1.]
[0. 0. 1. 0. 0. 0.]
[1. 0. 0. 0. 0. 0.]
[0. 0. 0. 0. 0. 1.]
[1. 0. 0. 0. 0. 0.]
[1. 0. 0. 0. 0. 0.]
[0. 1. 0. 0. 0. 0.]
[1. 0. 0. 0. 0. 0.]
[0. 1. 0. 0. 0. 0.]
[1. 0. 0. 0. 0. 0.]
[0. 1. 0. 0. 0. 0.]
[0. 0. 0. 0. 0. 1.]
[0. 0. 0. 0. 0. 1.]
[1. 0. 0. 0. 0. 0.]
[0. 0. 0. 0. 0. 1.]
[0. 0. 0. 0. 0. 1.]
[0. 1. 0. 0. 0. 0.]
[0. 0. 0. 0. 0. 1.]
[0. 0. 0. 0. 0. 1.]
[1. 0. 0. 0. 0. 0.]
[1. 0. 0. 0. 0. 0.]
[0. 0. 0. 0. 0. 1.]
[0. 0. 0. 0. 0. 1.]
[0. 0. 0. 0. 0. 1.]
[1. 0. 0. 0. 0. 0.]
[1. 0. 0. 0. 0. 0.]
[0. 0. 0. 0. 0. 1.]
[0. 0. 0. 0. 0. 1.]
[0. 0. 0. 0. 0. 1.]
```

```
 [0. 0. 0. 0. 0. 1.]
 [1. 0. 0. 0. 0. 0.]
 [0. 0. 0. 0. 0. 1.]
 [0. 0. 0. 0. 0. 1.]
 [0. 0. 0. 0. 0. 1.]
 [0. 0. 0. 0. 0. 1.]
 [0. 1. 0. 0. 0. 0.]
 [0. 0. 1. 0. 0. 0.]
 [0. 0. 0. 0. 0. 1.]
 [0. 0. 0. 0. 0. 1.]
 [0. 0. 0. 0. 0. 1.]
 [1. 0. 0. 0. 0. 0.]
 [0. 0. 0. 0. 0. 1.]
 [0. 0. 0. 1. 0. 0.]
 [0. 0. 0. 0. 0. 1.]
 [0. 0. 0. 0. 0. 1.]
 [0. 0. 0. 0. 0. 1.]
 [0. 0. 0. 0. 0. 1.]
 [0. 0. 1. 0. 0. 0.]
 [0. 0. 0. 0. 0. 1.]
 [0. 0. 1. 0. 0. 0.]
 [1. 0. 0. 0. 0. 0.]]
```

[5]:

```
# build a model
model = Sequential()
model.add(Dense(84, input_shape=(X_train.shape[1],), activation='relu')) #␣
↪input shape is (features,)
model.add(Dense(48, activation='relu'))
model.add(Dense(24, activation='relu'))
model.add(Dense(12, activation='relu'))
model.add(Dense(6, activation='softmax'))

model.summary()

learning_rate = 0.0001
optimizer = optimizers.Adam(learning_rate)
# compile the model
model.compile(optimizer = optimizer,
              #optimizer='rmsprop',
              loss='categorical_crossentropy', # this is different instead of␣
↪binary_crossentropy (for regular classification)
              metrics=['accuracy'])
```

```
Model: "sequential"

_________________________________________________________________
 Layer (type)                Output Shape              Param #
=================================================================
 dense (Dense)               (None, 84)                1176
```

```
 dense_1 (Dense)             (None, 48)                4080

 dense_2 (Dense)             (None, 24)                1176

 dense_3 (Dense)             (None, 12)                300

 dense_4 (Dense)             (None, 6)                 78

=================================================================
Total params: 6,810
Trainable params: 6,810
Non-trainable params: 0
_________________________________________________________________
```

[44]:

```
import keras
from keras.callbacks import EarlyStopping


es = keras.callbacks.EarlyStopping(monitor='val_loss',
                                   mode='min',
                                   patience=10,
                                   restore_best_weights=True) # important -
 ↪otherwise you just return the last weigths...

# now we just update our model fit call
history = model.fit(X_train,
                    dummy_y_train,
                    #callbacks=[es],
                    epochs=1000, # you can set this to a big number!
                    batch_size=10,
                    shuffle=True,
                    validation_split=0.2,
                    verbose=1)
```

```
Epoch 1/1000
145/145 [==============================] - 1s 7ms/step - loss: 0.0777 -
accuracy: 0.9724 - val_loss: 0.1842 - val_accuracy: 0.9421
Epoch 2/1000
145/145 [==============================] - 1s 6ms/step - loss: 0.0774 -
accuracy: 0.9724 - val_loss: 0.1794 - val_accuracy: 0.9421
Epoch 3/1000
145/145 [==============================] - 1s 6ms/step - loss: 0.0797 -
accuracy: 0.9710 - val_loss: 0.2045 - val_accuracy: 0.9339
Epoch 4/1000
145/145 [==============================] - 1s 6ms/step - loss: 0.0784 -
accuracy: 0.9710 - val_loss: 0.1673 - val_accuracy: 0.9421
Epoch 5/1000
```

```
145/145 [==============================] - 1s 6ms/step - loss: 0.0763 -
accuracy: 0.9710 - val_loss: 0.1836 - val_accuracy: 0.9366
Epoch 6/1000
145/145 [==============================] - 1s 7ms/step - loss: 0.0796 -
accuracy: 0.9717 - val_loss: 0.1877 - val_accuracy: 0.9421
Epoch 7/1000
145/145 [==============================] - 1s 6ms/step - loss: 0.0771 -
accuracy: 0.9703 - val_loss: 0.2098 - val_accuracy: 0.9449
Epoch 8/1000
145/145 [==============================] - 1s 6ms/step - loss: 0.0808 -
accuracy: 0.9710 - val_loss: 0.2481 - val_accuracy: 0.9394
Epoch 9/1000
145/145 [==============================] - 1s 6ms/step - loss: 0.0816 -
accuracy: 0.9731 - val_loss: 0.1740 - val_accuracy: 0.9421
Epoch 10/1000
145/145 [==============================] - 1s 6ms/step - loss: 0.0770 -
accuracy: 0.9710 - val_loss: 0.1691 - val_accuracy: 0.9449
Epoch 11/1000
145/145 [==============================] - 1s 6ms/step - loss: 0.0765 -
accuracy: 0.9710 - val_loss: 0.1726 - val_accuracy: 0.9421
Epoch 12/1000
145/145 [==============================] - 1s 6ms/step - loss: 0.0778 -
accuracy: 0.9710 - val_loss: 0.1865 - val_accuracy: 0.9449
Epoch 13/1000
145/145 [==============================] - 1s 7ms/step - loss: 0.0749 -
accuracy: 0.9703 - val_loss: 0.1754 - val_accuracy: 0.9366
Epoch 14/1000
145/145 [==============================] - 1s 8ms/step - loss: 0.0757 -
accuracy: 0.9724 - val_loss: 0.1797 - val_accuracy: 0.9366
Epoch 15/1000
145/145 [==============================] - 1s 6ms/step - loss: 0.0778 -
accuracy: 0.9724 - val_loss: 0.1984 - val_accuracy: 0.9394
Epoch 16/1000
145/145 [==============================] - 1s 7ms/step - loss: 0.0770 -
accuracy: 0.9717 - val_loss: 0.1734 - val_accuracy: 0.9449
Epoch 17/1000
145/145 [==============================] - 1s 7ms/step - loss: 0.0754 -
accuracy: 0.9724 - val_loss: 0.1726 - val_accuracy: 0.9449
Epoch 18/1000
145/145 [==============================] - 1s 6ms/step - loss: 0.0755 -
accuracy: 0.9738 - val_loss: 0.1873 - val_accuracy: 0.9394
Epoch 19/1000
145/145 [==============================] - 1s 7ms/step - loss: 0.0765 -
accuracy: 0.9696 - val_loss: 0.1683 - val_accuracy: 0.9394
Epoch 20/1000
145/145 [==============================] - 1s 5ms/step - loss: 0.0803 -
accuracy: 0.9703 - val_loss: 0.1649 - val_accuracy: 0.9449
Epoch 21/1000
```

```
145/145 [==============================] - 1s 4ms/step - loss: 0.0790 -
accuracy: 0.9689 - val_loss: 0.1883 - val_accuracy: 0.9394
Epoch 22/1000
145/145 [==============================] - 0s 3ms/step - loss: 0.0764 -
accuracy: 0.9703 - val_loss: 0.1629 - val_accuracy: 0.9366
Epoch 23/1000
145/145 [==============================] - 0s 3ms/step - loss: 0.0744 -
accuracy: 0.9683 - val_loss: 0.1763 - val_accuracy: 0.9366
Epoch 24/1000
145/145 [==============================] - 1s 4ms/step - loss: 0.0766 -
accuracy: 0.9710 - val_loss: 0.2032 - val_accuracy: 0.9339
Epoch 25/1000
145/145 [==============================] - 1s 4ms/step - loss: 0.0810 -
accuracy: 0.9696 - val_loss: 0.1918 - val_accuracy: 0.9366
Epoch 26/1000
145/145 [==============================] - 0s 3ms/step - loss: 0.0794 -
accuracy: 0.9696 - val_loss: 0.1987 - val_accuracy: 0.9421
Epoch 27/1000
145/145 [==============================] - 0s 3ms/step - loss: 0.0757 -
accuracy: 0.9703 - val_loss: 0.1782 - val_accuracy: 0.9366
Epoch 28/1000
145/145 [==============================] - 0s 3ms/step - loss: 0.0782 -
accuracy: 0.9717 - val_loss: 0.1655 - val_accuracy: 0.9421
Epoch 29/1000
145/145 [==============================] - 0s 3ms/step - loss: 0.0785 -
accuracy: 0.9738 - val_loss: 0.1694 - val_accuracy: 0.9421
Epoch 30/1000
145/145 [==============================] - 1s 4ms/step - loss: 0.0770 -
accuracy: 0.9717 - val_loss: 0.1698 - val_accuracy: 0.9366
Epoch 31/1000
145/145 [==============================] - 0s 3ms/step - loss: 0.0795 -
accuracy: 0.9683 - val_loss: 0.2091 - val_accuracy: 0.9449
Epoch 32/1000
145/145 [==============================] - 1s 4ms/step - loss: 0.0746 -
accuracy: 0.9731 - val_loss: 0.2064 - val_accuracy: 0.9394
Epoch 33/1000
145/145 [==============================] - 1s 4ms/step - loss: 0.0802 -
accuracy: 0.9689 - val_loss: 0.1656 - val_accuracy: 0.9449
Epoch 34/1000
145/145 [==============================] - 1s 4ms/step - loss: 0.0787 -
accuracy: 0.9738 - val_loss: 0.1710 - val_accuracy: 0.9421
Epoch 35/1000
145/145 [==============================] - 0s 3ms/step - loss: 0.0758 -
accuracy: 0.9731 - val_loss: 0.1689 - val_accuracy: 0.9366
Epoch 36/1000
145/145 [==============================] - 1s 4ms/step - loss: 0.0762 -
accuracy: 0.9724 - val_loss: 0.1711 - val_accuracy: 0.9366
Epoch 37/1000
```

```
145/145 [==============================] - 0s 3ms/step - loss: 0.0827 -
accuracy: 0.9662 - val_loss: 0.2174 - val_accuracy: 0.9366
Epoch 38/1000
145/145 [==============================] - 1s 4ms/step - loss: 0.0811 -
accuracy: 0.9717 - val_loss: 0.1896 - val_accuracy: 0.9394
Epoch 39/1000
145/145 [==============================] - 1s 4ms/step - loss: 0.0810 -
accuracy: 0.9669 - val_loss: 0.1910 - val_accuracy: 0.9366
Epoch 40/1000
145/145 [==============================] - 0s 3ms/step - loss: 0.0798 -
accuracy: 0.9717 - val_loss: 0.1926 - val_accuracy: 0.9366
Epoch 41/1000
145/145 [==============================] - 0s 3ms/step - loss: 0.0778 -
accuracy: 0.9710 - val_loss: 0.1664 - val_accuracy: 0.9366
Epoch 42/1000
145/145 [==============================] - 0s 3ms/step - loss: 0.0788 -
accuracy: 0.9696 - val_loss: 0.1781 - val_accuracy: 0.9366
Epoch 43/1000
145/145 [==============================] - 1s 4ms/step - loss: 0.0760 -
accuracy: 0.9724 - val_loss: 0.1828 - val_accuracy: 0.9366
Epoch 44/1000
145/145 [==============================] - 0s 3ms/step - loss: 0.0799 -
accuracy: 0.9689 - val_loss: 0.2019 - val_accuracy: 0.9394
Epoch 45/1000
145/145 [==============================] - 0s 3ms/step - loss: 0.0801 -
accuracy: 0.9703 - val_loss: 0.1937 - val_accuracy: 0.9421
Epoch 46/1000
145/145 [==============================] - 1s 4ms/step - loss: 0.0768 -
accuracy: 0.9676 - val_loss: 0.1880 - val_accuracy: 0.9449
Epoch 47/1000
145/145 [==============================] - 1s 4ms/step - loss: 0.0763 -
accuracy: 0.9731 - val_loss: 0.1832 - val_accuracy: 0.9394
Epoch 48/1000
145/145 [==============================] - 0s 3ms/step - loss: 0.0745 -
accuracy: 0.9731 - val_loss: 0.1702 - val_accuracy: 0.9421
Epoch 49/1000
145/145 [==============================] - 0s 3ms/step - loss: 0.0802 -
accuracy: 0.9696 - val_loss: 0.2026 - val_accuracy: 0.9449
Epoch 50/1000
145/145 [==============================] - 1s 4ms/step - loss: 0.0813 -
accuracy: 0.9689 - val_loss: 0.1989 - val_accuracy: 0.9449
Epoch 51/1000
145/145 [==============================] - 0s 3ms/step - loss: 0.0774 -
accuracy: 0.9703 - val_loss: 0.2082 - val_accuracy: 0.9449
Epoch 52/1000
145/145 [==============================] - 0s 3ms/step - loss: 0.0753 -
accuracy: 0.9710 - val_loss: 0.1990 - val_accuracy: 0.9421
Epoch 53/1000
```

```
145/145 [==============================] - 0s 3ms/step - loss: 0.0777 -
accuracy: 0.9738 - val_loss: 0.2210 - val_accuracy: 0.9394
Epoch 54/1000
145/145 [==============================] - 0s 3ms/step - loss: 0.0777 -
accuracy: 0.9696 - val_loss: 0.1839 - val_accuracy: 0.9366
Epoch 55/1000
145/145 [==============================] - 0s 3ms/step - loss: 0.0777 -
accuracy: 0.9724 - val_loss: 0.1747 - val_accuracy: 0.9421
Epoch 56/1000
145/145 [==============================] - 0s 3ms/step - loss: 0.0777 -
accuracy: 0.9738 - val_loss: 0.2081 - val_accuracy: 0.9449
Epoch 57/1000
145/145 [==============================] - 1s 3ms/step - loss: 0.0766 -
accuracy: 0.9724 - val_loss: 0.1817 - val_accuracy: 0.9421
Epoch 58/1000
145/145 [==============================] - 1s 4ms/step - loss: 0.0782 -
accuracy: 0.9696 - val_loss: 0.1849 - val_accuracy: 0.9449
Epoch 59/1000
145/145 [==============================] - 1s 4ms/step - loss: 0.0774 -
accuracy: 0.9689 - val_loss: 0.1893 - val_accuracy: 0.9394
Epoch 60/1000
145/145 [==============================] - 1s 4ms/step - loss: 0.0756 -
accuracy: 0.9731 - val_loss: 0.2176 - val_accuracy: 0.9449
Epoch 61/1000
145/145 [==============================] - 1s 4ms/step - loss: 0.0818 -
accuracy: 0.9669 - val_loss: 0.1847 - val_accuracy: 0.9311
Epoch 62/1000
145/145 [==============================] - 0s 3ms/step - loss: 0.0792 -
accuracy: 0.9696 - val_loss: 0.1884 - val_accuracy: 0.9394
Epoch 63/1000
145/145 [==============================] - 0s 3ms/step - loss: 0.0802 -
accuracy: 0.9717 - val_loss: 0.1743 - val_accuracy: 0.9366
Epoch 64/1000
145/145 [==============================] - 1s 4ms/step - loss: 0.0755 -
accuracy: 0.9703 - val_loss: 0.1785 - val_accuracy: 0.9366
Epoch 65/1000
145/145 [==============================] - 0s 3ms/step - loss: 0.0748 -
accuracy: 0.9724 - val_loss: 0.2159 - val_accuracy: 0.9394
Epoch 66/1000
145/145 [==============================] - 1s 4ms/step - loss: 0.0758 -
accuracy: 0.9710 - val_loss: 0.1766 - val_accuracy: 0.9366
Epoch 67/1000
145/145 [==============================] - 0s 3ms/step - loss: 0.0795 -
accuracy: 0.9710 - val_loss: 0.2119 - val_accuracy: 0.9339
Epoch 68/1000
145/145 [==============================] - 0s 3ms/step - loss: 0.0792 -
accuracy: 0.9717 - val_loss: 0.1756 - val_accuracy: 0.9421
Epoch 69/1000
```

```
145/145 [==============================] - 1s 4ms/step - loss: 0.0746 -
accuracy: 0.9731 - val_loss: 0.2089 - val_accuracy: 0.9366
Epoch 70/1000
145/145 [==============================] - 1s 4ms/step - loss: 0.0813 -
accuracy: 0.9703 - val_loss: 0.2046 - val_accuracy: 0.9449
Epoch 71/1000
145/145 [==============================] - 1s 4ms/step - loss: 0.0774 -
accuracy: 0.9696 - val_loss: 0.2160 - val_accuracy: 0.9394
Epoch 72/1000
145/145 [==============================] - 0s 3ms/step - loss: 0.0791 -
accuracy: 0.9703 - val_loss: 0.2129 - val_accuracy: 0.9339
Epoch 73/1000
145/145 [==============================] - 1s 4ms/step - loss: 0.0786 -
accuracy: 0.9662 - val_loss: 0.1834 - val_accuracy: 0.9366
Epoch 74/1000
145/145 [==============================] - 1s 4ms/step - loss: 0.0786 -
accuracy: 0.9717 - val_loss: 0.2064 - val_accuracy: 0.9449
Epoch 75/1000
145/145 [==============================] - 1s 4ms/step - loss: 0.0770 -
accuracy: 0.9710 - val_loss: 0.1944 - val_accuracy: 0.9366
Epoch 76/1000
145/145 [==============================] - 1s 4ms/step - loss: 0.0792 -
accuracy: 0.9724 - val_loss: 0.1893 - val_accuracy: 0.9394
Epoch 77/1000
145/145 [==============================] - 0s 3ms/step - loss: 0.0786 -
accuracy: 0.9717 - val_loss: 0.1755 - val_accuracy: 0.9366
Epoch 78/1000
145/145 [==============================] - 1s 4ms/step - loss: 0.0787 -
accuracy: 0.9683 - val_loss: 0.2107 - val_accuracy: 0.9366
Epoch 79/1000
145/145 [==============================] - 0s 3ms/step - loss: 0.0744 -
accuracy: 0.9738 - val_loss: 0.1721 - val_accuracy: 0.9421
Epoch 80/1000
145/145 [==============================] - 1s 4ms/step - loss: 0.0762 -
accuracy: 0.9710 - val_loss: 0.1717 - val_accuracy: 0.9311
Epoch 81/1000
145/145 [==============================] - 1s 4ms/step - loss: 0.0766 -
accuracy: 0.9745 - val_loss: 0.1808 - val_accuracy: 0.9394
Epoch 82/1000
145/145 [==============================] - 1s 3ms/step - loss: 0.0790 -
accuracy: 0.9724 - val_loss: 0.1782 - val_accuracy: 0.9366
Epoch 83/1000
145/145 [==============================] - 1s 4ms/step - loss: 0.0774 -
accuracy: 0.9703 - val_loss: 0.1749 - val_accuracy: 0.9449
Epoch 84/1000
145/145 [==============================] - 0s 3ms/step - loss: 0.0774 -
accuracy: 0.9724 - val_loss: 0.1779 - val_accuracy: 0.9421
Epoch 85/1000
```

```
145/145 [==============================] - 1s 4ms/step - loss: 0.0772 -
accuracy: 0.9703 - val_loss: 0.1756 - val_accuracy: 0.9421
Epoch 86/1000
145/145 [==============================] - 0s 3ms/step - loss: 0.0773 -
accuracy: 0.9696 - val_loss: 0.1741 - val_accuracy: 0.9421
Epoch 87/1000
145/145 [==============================] - 0s 3ms/step - loss: 0.0781 -
accuracy: 0.9683 - val_loss: 0.1847 - val_accuracy: 0.9366
Epoch 88/1000
145/145 [==============================] - 0s 3ms/step - loss: 0.0769 -
accuracy: 0.9717 - val_loss: 0.1812 - val_accuracy: 0.9366
Epoch 89/1000
145/145 [==============================] - 1s 4ms/step - loss: 0.0744 -
accuracy: 0.9738 - val_loss: 0.1763 - val_accuracy: 0.9366
Epoch 90/1000
145/145 [==============================] - 0s 3ms/step - loss: 0.0771 -
accuracy: 0.9731 - val_loss: 0.1885 - val_accuracy: 0.9394
Epoch 91/1000
145/145 [==============================] - 0s 3ms/step - loss: 0.0758 -
accuracy: 0.9717 - val_loss: 0.1747 - val_accuracy: 0.9366
Epoch 92/1000
145/145 [==============================] - 1s 4ms/step - loss: 0.0754 -
accuracy: 0.9731 - val_loss: 0.2014 - val_accuracy: 0.9449
Epoch 93/1000
145/145 [==============================] - 0s 3ms/step - loss: 0.0786 -
accuracy: 0.9710 - val_loss: 0.2116 - val_accuracy: 0.9449
Epoch 94/1000
145/145 [==============================] - 1s 4ms/step - loss: 0.0733 -
accuracy: 0.9724 - val_loss: 0.1859 - val_accuracy: 0.9366
Epoch 95/1000
145/145 [==============================] - 0s 3ms/step - loss: 0.0827 -
accuracy: 0.9703 - val_loss: 0.1854 - val_accuracy: 0.9394
Epoch 96/1000
145/145 [==============================] - 1s 4ms/step - loss: 0.0765 -
accuracy: 0.9731 - val_loss: 0.1709 - val_accuracy: 0.9421
Epoch 97/1000
145/145 [==============================] - 1s 4ms/step - loss: 0.0793 -
accuracy: 0.9703 - val_loss: 0.1995 - val_accuracy: 0.9449
Epoch 98/1000
145/145 [==============================] - 0s 3ms/step - loss: 0.0769 -
accuracy: 0.9703 - val_loss: 0.1896 - val_accuracy: 0.9394
Epoch 99/1000
145/145 [==============================] - 0s 3ms/step - loss: 0.0782 -
accuracy: 0.9696 - val_loss: 0.1964 - val_accuracy: 0.9449
Epoch 100/1000
145/145 [==============================] - 1s 4ms/step - loss: 0.0771 -
accuracy: 0.9683 - val_loss: 0.1741 - val_accuracy: 0.9366
Epoch 101/1000
```

```
145/145 [==============================] - 0s 3ms/step - loss: 0.0799 -
accuracy: 0.9696 - val_loss: 0.1895 - val_accuracy: 0.9394
Epoch 102/1000
145/145 [==============================] - 0s 3ms/step - loss: 0.0771 -
accuracy: 0.9738 - val_loss: 0.1798 - val_accuracy: 0.9366
Epoch 103/1000
145/145 [==============================] - 1s 3ms/step - loss: 0.0758 -
accuracy: 0.9724 - val_loss: 0.1716 - val_accuracy: 0.9421
Epoch 104/1000
145/145 [==============================] - 0s 3ms/step - loss: 0.0779 -
accuracy: 0.9689 - val_loss: 0.2011 - val_accuracy: 0.9339
Epoch 105/1000
145/145 [==============================] - 1s 4ms/step - loss: 0.0772 -
accuracy: 0.9738 - val_loss: 0.1771 - val_accuracy: 0.9449
Epoch 106/1000
145/145 [==============================] - 0s 3ms/step - loss: 0.0746 -
accuracy: 0.9717 - val_loss: 0.2056 - val_accuracy: 0.9339
Epoch 107/1000
145/145 [==============================] - 1s 3ms/step - loss: 0.0789 -
accuracy: 0.9717 - val_loss: 0.1708 - val_accuracy: 0.9366
Epoch 108/1000
145/145 [==============================] - 0s 3ms/step - loss: 0.0804 -
accuracy: 0.9724 - val_loss: 0.1980 - val_accuracy: 0.9366
Epoch 109/1000
145/145 [==============================] - 1s 4ms/step - loss: 0.0791 -
accuracy: 0.9696 - val_loss: 0.1878 - val_accuracy: 0.9394
Epoch 110/1000
145/145 [==============================] - 1s 4ms/step - loss: 0.0779 -
accuracy: 0.9717 - val_loss: 0.1929 - val_accuracy: 0.9394
Epoch 111/1000
145/145 [==============================] - 0s 3ms/step - loss: 0.0779 -
accuracy: 0.9689 - val_loss: 0.1766 - val_accuracy: 0.9366
Epoch 112/1000
145/145 [==============================] - 0s 3ms/step - loss: 0.0770 -
accuracy: 0.9710 - val_loss: 0.2366 - val_accuracy: 0.9284
Epoch 113/1000
145/145 [==============================] - 1s 4ms/step - loss: 0.0771 -
accuracy: 0.9717 - val_loss: 0.1750 - val_accuracy: 0.9421
Epoch 114/1000
145/145 [==============================] - 0s 3ms/step - loss: 0.0759 -
accuracy: 0.9703 - val_loss: 0.1767 - val_accuracy: 0.9421
Epoch 115/1000
145/145 [==============================] - 1s 4ms/step - loss: 0.0782 -
accuracy: 0.9717 - val_loss: 0.1704 - val_accuracy: 0.9421
Epoch 116/1000
145/145 [==============================] - 0s 3ms/step - loss: 0.0789 -
accuracy: 0.9689 - val_loss: 0.1865 - val_accuracy: 0.9449
Epoch 117/1000
```

```
145/145 [==============================] - 0s 3ms/step - loss: 0.0764 -
accuracy: 0.9717 - val_loss: 0.1858 - val_accuracy: 0.9366
Epoch 118/1000
145/145 [==============================] - 1s 4ms/step - loss: 0.0824 -
accuracy: 0.9689 - val_loss: 0.1925 - val_accuracy: 0.9311
Epoch 119/1000
145/145 [==============================] - 0s 3ms/step - loss: 0.0802 -
accuracy: 0.9696 - val_loss: 0.1771 - val_accuracy: 0.9394
Epoch 120/1000
145/145 [==============================] - 1s 4ms/step - loss: 0.0778 -
accuracy: 0.9689 - val_loss: 0.1770 - val_accuracy: 0.9421
Epoch 121/1000
145/145 [==============================] - 0s 3ms/step - loss: 0.0765 -
accuracy: 0.9724 - val_loss: 0.1712 - val_accuracy: 0.9449
Epoch 122/1000
145/145 [==============================] - 0s 3ms/step - loss: 0.0755 -
accuracy: 0.9710 - val_loss: 0.1965 - val_accuracy: 0.9394
Epoch 123/1000
145/145 [==============================] - 0s 3ms/step - loss: 0.0755 -
accuracy: 0.9717 - val_loss: 0.1955 - val_accuracy: 0.9394
Epoch 124/1000
145/145 [==============================] - 1s 4ms/step - loss: 0.0740 -
accuracy: 0.9745 - val_loss: 0.1729 - val_accuracy: 0.9421
Epoch 125/1000
145/145 [==============================] - 0s 3ms/step - loss: 0.0756 -
accuracy: 0.9717 - val_loss: 0.1808 - val_accuracy: 0.9421
Epoch 126/1000
145/145 [==============================] - 0s 3ms/step - loss: 0.0766 -
accuracy: 0.9710 - val_loss: 0.1877 - val_accuracy: 0.9366
Epoch 127/1000
145/145 [==============================] - 0s 3ms/step - loss: 0.0759 -
accuracy: 0.9703 - val_loss: 0.1852 - val_accuracy: 0.9394
Epoch 128/1000
145/145 [==============================] - 0s 3ms/step - loss: 0.0751 -
accuracy: 0.9710 - val_loss: 0.1954 - val_accuracy: 0.9394
Epoch 129/1000
145/145 [==============================] - 1s 4ms/step - loss: 0.0779 -
accuracy: 0.9731 - val_loss: 0.1771 - val_accuracy: 0.9421
Epoch 130/1000
145/145 [==============================] - 0s 3ms/step - loss: 0.0764 -
accuracy: 0.9703 - val_loss: 0.1884 - val_accuracy: 0.9449
Epoch 131/1000
145/145 [==============================] - 1s 4ms/step - loss: 0.0757 -
accuracy: 0.9696 - val_loss: 0.1950 - val_accuracy: 0.9394
Epoch 132/1000
145/145 [==============================] - 0s 3ms/step - loss: 0.0768 -
accuracy: 0.9717 - val_loss: 0.1618 - val_accuracy: 0.9421
Epoch 133/1000
```

```
145/145 [==============================] - 0s 3ms/step - loss: 0.0781 -
accuracy: 0.9696 - val_loss: 0.1737 - val_accuracy: 0.9394
Epoch 134/1000
145/145 [==============================] - 0s 3ms/step - loss: 0.0773 -
accuracy: 0.9696 - val_loss: 0.1980 - val_accuracy: 0.9394
Epoch 135/1000
145/145 [==============================] - 0s 3ms/step - loss: 0.0779 -
accuracy: 0.9696 - val_loss: 0.1812 - val_accuracy: 0.9366
Epoch 136/1000
145/145 [==============================] - 0s 3ms/step - loss: 0.0785 -
accuracy: 0.9717 - val_loss: 0.1690 - val_accuracy: 0.9366
Epoch 137/1000
145/145 [==============================] - 1s 4ms/step - loss: 0.0755 -
accuracy: 0.9724 - val_loss: 0.1843 - val_accuracy: 0.9366
Epoch 138/1000
145/145 [==============================] - 0s 3ms/step - loss: 0.0784 -
accuracy: 0.9689 - val_loss: 0.1718 - val_accuracy: 0.9394
Epoch 139/1000
145/145 [==============================] - 0s 3ms/step - loss: 0.0785 -
accuracy: 0.9703 - val_loss: 0.1920 - val_accuracy: 0.9394
Epoch 140/1000
145/145 [==============================] - 1s 4ms/step - loss: 0.0729 -
accuracy: 0.9710 - val_loss: 0.1711 - val_accuracy: 0.9366
Epoch 141/1000
145/145 [==============================] - 1s 4ms/step - loss: 0.0746 -
accuracy: 0.9717 - val_loss: 0.2040 - val_accuracy: 0.9339
Epoch 142/1000
145/145 [==============================] - 0s 3ms/step - loss: 0.0777 -
accuracy: 0.9683 - val_loss: 0.2105 - val_accuracy: 0.9449
Epoch 143/1000
145/145 [==============================] - 0s 3ms/step - loss: 0.0816 -
accuracy: 0.9696 - val_loss: 0.1923 - val_accuracy: 0.9449
Epoch 144/1000
145/145 [==============================] - 1s 4ms/step - loss: 0.0777 -
accuracy: 0.9710 - val_loss: 0.1721 - val_accuracy: 0.9421
Epoch 145/1000
145/145 [==============================] - 0s 3ms/step - loss: 0.0763 -
accuracy: 0.9703 - val_loss: 0.1839 - val_accuracy: 0.9394
Epoch 146/1000
145/145 [==============================] - 1s 4ms/step - loss: 0.0804 -
accuracy: 0.9683 - val_loss: 0.1992 - val_accuracy: 0.9394
Epoch 147/1000
145/145 [==============================] - 1s 4ms/step - loss: 0.0771 -
accuracy: 0.9724 - val_loss: 0.1904 - val_accuracy: 0.9394
Epoch 148/1000
145/145 [==============================] - 1s 3ms/step - loss: 0.0749 -
accuracy: 0.9683 - val_loss: 0.1624 - val_accuracy: 0.9449
Epoch 149/1000
```

```
145/145 [==============================] - 1s 4ms/step - loss: 0.0780 -
accuracy: 0.9669 - val_loss: 0.1900 - val_accuracy: 0.9449
Epoch 150/1000
145/145 [==============================] - 1s 4ms/step - loss: 0.0774 -
accuracy: 0.9717 - val_loss: 0.1742 - val_accuracy: 0.9449
Epoch 151/1000
145/145 [==============================] - 1s 4ms/step - loss: 0.0784 -
accuracy: 0.9710 - val_loss: 0.1851 - val_accuracy: 0.9449
Epoch 152/1000
145/145 [==============================] - 1s 3ms/step - loss: 0.0761 -
accuracy: 0.9717 - val_loss: 0.1945 - val_accuracy: 0.9449
Epoch 153/1000
145/145 [==============================] - 0s 3ms/step - loss: 0.0786 -
accuracy: 0.9703 - val_loss: 0.1956 - val_accuracy: 0.9394
Epoch 154/1000
145/145 [==============================] - 0s 3ms/step - loss: 0.0772 -
accuracy: 0.9676 - val_loss: 0.2124 - val_accuracy: 0.9394
Epoch 155/1000
145/145 [==============================] - 0s 3ms/step - loss: 0.0755 -
accuracy: 0.9717 - val_loss: 0.1823 - val_accuracy: 0.9366
Epoch 156/1000
145/145 [==============================] - 0s 3ms/step - loss: 0.0776 -
accuracy: 0.9731 - val_loss: 0.1775 - val_accuracy: 0.9421
Epoch 157/1000
145/145 [==============================] - 1s 4ms/step - loss: 0.0764 -
accuracy: 0.9703 - val_loss: 0.1835 - val_accuracy: 0.9394
Epoch 158/1000
145/145 [==============================] - 0s 3ms/step - loss: 0.0771 -
accuracy: 0.9717 - val_loss: 0.1954 - val_accuracy: 0.9394
Epoch 159/1000
145/145 [==============================] - 1s 4ms/step - loss: 0.0775 -
accuracy: 0.9703 - val_loss: 0.1826 - val_accuracy: 0.9366
Epoch 160/1000
145/145 [==============================] - 0s 3ms/step - loss: 0.0801 -
accuracy: 0.9724 - val_loss: 0.2073 - val_accuracy: 0.9449
Epoch 161/1000
145/145 [==============================] - 1s 4ms/step - loss: 0.0774 -
accuracy: 0.9710 - val_loss: 0.1741 - val_accuracy: 0.9449
Epoch 162/1000
145/145 [==============================] - 0s 3ms/step - loss: 0.0744 -
accuracy: 0.9703 - val_loss: 0.1847 - val_accuracy: 0.9394
Epoch 163/1000
145/145 [==============================] - 1s 4ms/step - loss: 0.0791 -
accuracy: 0.9724 - val_loss: 0.2035 - val_accuracy: 0.9394
Epoch 164/1000
145/145 [==============================] - 0s 3ms/step - loss: 0.0789 -
accuracy: 0.9689 - val_loss: 0.1886 - val_accuracy: 0.9366
Epoch 165/1000
```

```
145/145 [==============================] - 1s 4ms/step - loss: 0.0771 -
accuracy: 0.9731 - val_loss: 0.1748 - val_accuracy: 0.9449
Epoch 166/1000
145/145 [==============================] - 0s 3ms/step - loss: 0.0764 -
accuracy: 0.9717 - val_loss: 0.1757 - val_accuracy: 0.9339
Epoch 167/1000
145/145 [==============================] - 0s 3ms/step - loss: 0.0776 -
accuracy: 0.9710 - val_loss: 0.2090 - val_accuracy: 0.9449
Epoch 168/1000
145/145 [==============================] - 1s 4ms/step - loss: 0.0775 -
accuracy: 0.9724 - val_loss: 0.1911 - val_accuracy: 0.9449
Epoch 169/1000
145/145 [==============================] - 0s 3ms/step - loss: 0.0781 -
accuracy: 0.9710 - val_loss: 0.1961 - val_accuracy: 0.9366
Epoch 170/1000
145/145 [==============================] - 1s 4ms/step - loss: 0.0770 -
accuracy: 0.9710 - val_loss: 0.1927 - val_accuracy: 0.9449
Epoch 171/1000
145/145 [==============================] - 0s 3ms/step - loss: 0.0764 -
accuracy: 0.9717 - val_loss: 0.1887 - val_accuracy: 0.9449
Epoch 172/1000
145/145 [==============================] - 1s 3ms/step - loss: 0.0746 -
accuracy: 0.9710 - val_loss: 0.1690 - val_accuracy: 0.9449
Epoch 173/1000
145/145 [==============================] - 0s 3ms/step - loss: 0.0770 -
accuracy: 0.9717 - val_loss: 0.2048 - val_accuracy: 0.9394
Epoch 174/1000
145/145 [==============================] - 1s 4ms/step - loss: 0.0756 -
accuracy: 0.9683 - val_loss: 0.1889 - val_accuracy: 0.9421
Epoch 175/1000
145/145 [==============================] - 1s 4ms/step - loss: 0.0760 -
accuracy: 0.9717 - val_loss: 0.1997 - val_accuracy: 0.9421
Epoch 176/1000
145/145 [==============================] - 1s 6ms/step - loss: 0.0768 -
accuracy: 0.9710 - val_loss: 0.1786 - val_accuracy: 0.9421
Epoch 177/1000
145/145 [==============================] - 1s 7ms/step - loss: 0.0771 -
accuracy: 0.9703 - val_loss: 0.1862 - val_accuracy: 0.9449
Epoch 178/1000
145/145 [==============================] - 1s 4ms/step - loss: 0.0768 -
accuracy: 0.9710 - val_loss: 0.1810 - val_accuracy: 0.9339
Epoch 179/1000
145/145 [==============================] - 1s 7ms/step - loss: 0.0781 -
accuracy: 0.9703 - val_loss: 0.1825 - val_accuracy: 0.9449
Epoch 180/1000
145/145 [==============================] - 0s 3ms/step - loss: 0.0745 -
accuracy: 0.9731 - val_loss: 0.1793 - val_accuracy: 0.9449
Epoch 181/1000
```

```
145/145 [==============================] - 1s 4ms/step - loss: 0.0734 -
accuracy: 0.9689 - val_loss: 0.2010 - val_accuracy: 0.9449
Epoch 182/1000
145/145 [==============================] - 1s 4ms/step - loss: 0.0793 -
accuracy: 0.9696 - val_loss: 0.1846 - val_accuracy: 0.9449
Epoch 183/1000
145/145 [==============================] - 0s 3ms/step - loss: 0.0739 -
accuracy: 0.9703 - val_loss: 0.1914 - val_accuracy: 0.9449
Epoch 184/1000
145/145 [==============================] - 1s 4ms/step - loss: 0.0814 -
accuracy: 0.9696 - val_loss: 0.1796 - val_accuracy: 0.9366
Epoch 185/1000
145/145 [==============================] - 1s 4ms/step - loss: 0.0780 -
accuracy: 0.9676 - val_loss: 0.1827 - val_accuracy: 0.9311
Epoch 186/1000
145/145 [==============================] - 1s 4ms/step - loss: 0.0781 -
accuracy: 0.9710 - val_loss: 0.1858 - val_accuracy: 0.9449
Epoch 187/1000
145/145 [==============================] - 1s 3ms/step - loss: 0.0759 -
accuracy: 0.9710 - val_loss: 0.1847 - val_accuracy: 0.9449
Epoch 188/1000
145/145 [==============================] - 1s 4ms/step - loss: 0.0746 -
accuracy: 0.9710 - val_loss: 0.2007 - val_accuracy: 0.9394
Epoch 189/1000
145/145 [==============================] - 1s 4ms/step - loss: 0.0827 -
accuracy: 0.9689 - val_loss: 0.1940 - val_accuracy: 0.9449
Epoch 190/1000
145/145 [==============================] - 1s 4ms/step - loss: 0.0768 -
accuracy: 0.9703 - val_loss: 0.1813 - val_accuracy: 0.9449
Epoch 191/1000
145/145 [==============================] - 1s 4ms/step - loss: 0.0756 -
accuracy: 0.9724 - val_loss: 0.1731 - val_accuracy: 0.9449
Epoch 192/1000
145/145 [==============================] - 1s 4ms/step - loss: 0.0754 -
accuracy: 0.9731 - val_loss: 0.1728 - val_accuracy: 0.9449
Epoch 193/1000
145/145 [==============================] - 0s 3ms/step - loss: 0.0756 -
accuracy: 0.9731 - val_loss: 0.1910 - val_accuracy: 0.9366
Epoch 194/1000
145/145 [==============================] - 1s 3ms/step - loss: 0.0748 -
accuracy: 0.9731 - val_loss: 0.1770 - val_accuracy: 0.9366
Epoch 195/1000
145/145 [==============================] - 0s 3ms/step - loss: 0.0767 -
accuracy: 0.9710 - val_loss: 0.1756 - val_accuracy: 0.9394
Epoch 196/1000
145/145 [==============================] - 1s 4ms/step - loss: 0.0780 -
accuracy: 0.9689 - val_loss: 0.1991 - val_accuracy: 0.9449
Epoch 197/1000
```

```
145/145 [==============================] - 1s 4ms/step - loss: 0.0774 -
accuracy: 0.9724 - val_loss: 0.1791 - val_accuracy: 0.9449
Epoch 198/1000
145/145 [==============================] - 1s 4ms/step - loss: 0.0768 -
accuracy: 0.9738 - val_loss: 0.1792 - val_accuracy: 0.9449
Epoch 199/1000
145/145 [==============================] - 1s 4ms/step - loss: 0.0757 -
accuracy: 0.9717 - val_loss: 0.1668 - val_accuracy: 0.9449
Epoch 200/1000
145/145 [==============================] - 1s 3ms/step - loss: 0.0772 -
accuracy: 0.9710 - val_loss: 0.1915 - val_accuracy: 0.9449
Epoch 201/1000
145/145 [==============================] - 1s 4ms/step - loss: 0.0747 -
accuracy: 0.9710 - val_loss: 0.1845 - val_accuracy: 0.9366
Epoch 202/1000
145/145 [==============================] - 1s 4ms/step - loss: 0.0763 -
accuracy: 0.9717 - val_loss: 0.1660 - val_accuracy: 0.9449
Epoch 203/1000
145/145 [==============================] - 1s 4ms/step - loss: 0.0753 -
accuracy: 0.9689 - val_loss: 0.1825 - val_accuracy: 0.9394
Epoch 204/1000
145/145 [==============================] - 0s 3ms/step - loss: 0.0759 -
accuracy: 0.9745 - val_loss: 0.1707 - val_accuracy: 0.9366
Epoch 205/1000
145/145 [==============================] - 0s 3ms/step - loss: 0.0744 -
accuracy: 0.9731 - val_loss: 0.2151 - val_accuracy: 0.9394
Epoch 206/1000
145/145 [==============================] - 0s 3ms/step - loss: 0.0769 -
accuracy: 0.9710 - val_loss: 0.1630 - val_accuracy: 0.9421
Epoch 207/1000
145/145 [==============================] - 1s 3ms/step - loss: 0.0743 -
accuracy: 0.9717 - val_loss: 0.1791 - val_accuracy: 0.9421
Epoch 208/1000
145/145 [==============================] - 0s 3ms/step - loss: 0.0801 -
accuracy: 0.9696 - val_loss: 0.1694 - val_accuracy: 0.9421
Epoch 209/1000
145/145 [==============================] - 1s 4ms/step - loss: 0.0773 -
accuracy: 0.9724 - val_loss: 0.1600 - val_accuracy: 0.9366
Epoch 210/1000
145/145 [==============================] - 0s 3ms/step - loss: 0.0784 -
accuracy: 0.9689 - val_loss: 0.1824 - val_accuracy: 0.9366
Epoch 211/1000
145/145 [==============================] - 1s 3ms/step - loss: 0.0782 -
accuracy: 0.9717 - val_loss: 0.1937 - val_accuracy: 0.9394
Epoch 212/1000
145/145 [==============================] - 0s 3ms/step - loss: 0.0743 -
accuracy: 0.9731 - val_loss: 0.1819 - val_accuracy: 0.9366
Epoch 213/1000
```

```
145/145 [==============================] - 1s 3ms/step - loss: 0.0771 -
accuracy: 0.9710 - val_loss: 0.1776 - val_accuracy: 0.9366
Epoch 214/1000
145/145 [==============================] - 0s 3ms/step - loss: 0.0767 -
accuracy: 0.9738 - val_loss: 0.1715 - val_accuracy: 0.9449
Epoch 215/1000
145/145 [==============================] - 1s 4ms/step - loss: 0.0753 -
accuracy: 0.9710 - val_loss: 0.1698 - val_accuracy: 0.9366
Epoch 216/1000
145/145 [==============================] - 0s 3ms/step - loss: 0.0769 -
accuracy: 0.9703 - val_loss: 0.1733 - val_accuracy: 0.9449
Epoch 217/1000
145/145 [==============================] - 0s 3ms/step - loss: 0.0764 -
accuracy: 0.9696 - val_loss: 0.1813 - val_accuracy: 0.9366
Epoch 218/1000
145/145 [==============================] - 0s 3ms/step - loss: 0.0772 -
accuracy: 0.9703 - val_loss: 0.1853 - val_accuracy: 0.9394
Epoch 219/1000
145/145 [==============================] - 1s 4ms/step - loss: 0.0804 -
accuracy: 0.9683 - val_loss: 0.1710 - val_accuracy: 0.9366
Epoch 220/1000
145/145 [==============================] - 0s 3ms/step - loss: 0.0767 -
accuracy: 0.9710 - val_loss: 0.1825 - val_accuracy: 0.9366
Epoch 221/1000
145/145 [==============================] - 1s 4ms/step - loss: 0.0768 -
accuracy: 0.9731 - val_loss: 0.1924 - val_accuracy: 0.9394
Epoch 222/1000
145/145 [==============================] - 1s 4ms/step - loss: 0.0760 -
accuracy: 0.9724 - val_loss: 0.1903 - val_accuracy: 0.9394
Epoch 223/1000
145/145 [==============================] - 1s 4ms/step - loss: 0.0748 -
accuracy: 0.9696 - val_loss: 0.1702 - val_accuracy: 0.9366
Epoch 224/1000
145/145 [==============================] - 1s 4ms/step - loss: 0.0793 -
accuracy: 0.9703 - val_loss: 0.1975 - val_accuracy: 0.9449
Epoch 225/1000
145/145 [==============================] - 1s 4ms/step - loss: 0.0765 -
accuracy: 0.9724 - val_loss: 0.1836 - val_accuracy: 0.9394
Epoch 226/1000
145/145 [==============================] - 1s 4ms/step - loss: 0.0745 -
accuracy: 0.9717 - val_loss: 0.1866 - val_accuracy: 0.9449
Epoch 227/1000
145/145 [==============================] - 0s 3ms/step - loss: 0.0736 -
accuracy: 0.9731 - val_loss: 0.1783 - val_accuracy: 0.9449
Epoch 228/1000
145/145 [==============================] - 1s 3ms/step - loss: 0.0752 -
accuracy: 0.9724 - val_loss: 0.1949 - val_accuracy: 0.9449
Epoch 229/1000
```

```
145/145 [==============================] - 1s 4ms/step - loss: 0.0772 -
accuracy: 0.9710 - val_loss: 0.1893 - val_accuracy: 0.9449
Epoch 230/1000
145/145 [==============================] - 1s 4ms/step - loss: 0.0772 -
accuracy: 0.9724 - val_loss: 0.1929 - val_accuracy: 0.9394
Epoch 231/1000
145/145 [==============================] - 0s 3ms/step - loss: 0.0774 -
accuracy: 0.9731 - val_loss: 0.1925 - val_accuracy: 0.9311
Epoch 232/1000
145/145 [==============================] - 1s 4ms/step - loss: 0.0754 -
accuracy: 0.9717 - val_loss: 0.1746 - val_accuracy: 0.9421
Epoch 233/1000
145/145 [==============================] - 1s 4ms/step - loss: 0.0761 -
accuracy: 0.9696 - val_loss: 0.1824 - val_accuracy: 0.9449
Epoch 234/1000
145/145 [==============================] - 0s 3ms/step - loss: 0.0736 -
accuracy: 0.9717 - val_loss: 0.1672 - val_accuracy: 0.9421
Epoch 235/1000
145/145 [==============================] - 1s 4ms/step - loss: 0.0783 -
accuracy: 0.9703 - val_loss: 0.1749 - val_accuracy: 0.9421
Epoch 236/1000
145/145 [==============================] - 1s 4ms/step - loss: 0.0789 -
accuracy: 0.9717 - val_loss: 0.1982 - val_accuracy: 0.9449
Epoch 237/1000
145/145 [==============================] - 0s 3ms/step - loss: 0.0775 -
accuracy: 0.9703 - val_loss: 0.1824 - val_accuracy: 0.9394
Epoch 238/1000
145/145 [==============================] - 1s 3ms/step - loss: 0.0764 -
accuracy: 0.9696 - val_loss: 0.1965 - val_accuracy: 0.9394
Epoch 239/1000
145/145 [==============================] - 1s 3ms/step - loss: 0.0736 -
accuracy: 0.9717 - val_loss: 0.2064 - val_accuracy: 0.9449
Epoch 240/1000
145/145 [==============================] - 1s 3ms/step - loss: 0.0757 -
accuracy: 0.9683 - val_loss: 0.1970 - val_accuracy: 0.9449
Epoch 241/1000
145/145 [==============================] - 0s 3ms/step - loss: 0.0742 -
accuracy: 0.9745 - val_loss: 0.1756 - val_accuracy: 0.9339
Epoch 242/1000
145/145 [==============================] - 1s 4ms/step - loss: 0.0777 -
accuracy: 0.9724 - val_loss: 0.1957 - val_accuracy: 0.9394
Epoch 243/1000
145/145 [==============================] - 1s 4ms/step - loss: 0.0764 -
accuracy: 0.9703 - val_loss: 0.1875 - val_accuracy: 0.9449
Epoch 244/1000
145/145 [==============================] - 1s 3ms/step - loss: 0.0758 -
accuracy: 0.9710 - val_loss: 0.1791 - val_accuracy: 0.9449
Epoch 245/1000
```

```
145/145 [==============================] - 0s 3ms/step - loss: 0.0765 -
accuracy: 0.9710 - val_loss: 0.2111 - val_accuracy: 0.9394
Epoch 246/1000
145/145 [==============================] - 0s 3ms/step - loss: 0.0768 -
accuracy: 0.9724 - val_loss: 0.1969 - val_accuracy: 0.9339
Epoch 247/1000
145/145 [==============================] - 1s 4ms/step - loss: 0.0772 -
accuracy: 0.9696 - val_loss: 0.2118 - val_accuracy: 0.9449
Epoch 248/1000
145/145 [==============================] - 1s 3ms/step - loss: 0.0762 -
accuracy: 0.9717 - val_loss: 0.1826 - val_accuracy: 0.9366
Epoch 249/1000
145/145 [==============================] - 1s 4ms/step - loss: 0.0759 -
accuracy: 0.9717 - val_loss: 0.2015 - val_accuracy: 0.9339
Epoch 250/1000
145/145 [==============================] - 0s 3ms/step - loss: 0.0770 -
accuracy: 0.9710 - val_loss: 0.1838 - val_accuracy: 0.9421
Epoch 251/1000
145/145 [==============================] - 0s 3ms/step - loss: 0.0756 -
accuracy: 0.9717 - val_loss: 0.1951 - val_accuracy: 0.9449
Epoch 252/1000
145/145 [==============================] - 1s 4ms/step - loss: 0.0778 -
accuracy: 0.9731 - val_loss: 0.1862 - val_accuracy: 0.9366
Epoch 253/1000
145/145 [==============================] - 1s 4ms/step - loss: 0.0751 -
accuracy: 0.9717 - val_loss: 0.1898 - val_accuracy: 0.9449
Epoch 254/1000
145/145 [==============================] - 1s 4ms/step - loss: 0.0772 -
accuracy: 0.9689 - val_loss: 0.2006 - val_accuracy: 0.9449
Epoch 255/1000
145/145 [==============================] - 1s 4ms/step - loss: 0.0760 -
accuracy: 0.9710 - val_loss: 0.1915 - val_accuracy: 0.9449
Epoch 256/1000
145/145 [==============================] - 0s 3ms/step - loss: 0.0793 -
accuracy: 0.9689 - val_loss: 0.1974 - val_accuracy: 0.9449
Epoch 257/1000
145/145 [==============================] - 1s 4ms/step - loss: 0.0772 -
accuracy: 0.9731 - val_loss: 0.1878 - val_accuracy: 0.9394
Epoch 258/1000
145/145 [==============================] - 1s 4ms/step - loss: 0.0751 -
accuracy: 0.9710 - val_loss: 0.2007 - val_accuracy: 0.9449
Epoch 259/1000
145/145 [==============================] - 1s 4ms/step - loss: 0.0760 -
accuracy: 0.9710 - val_loss: 0.1985 - val_accuracy: 0.9394
Epoch 260/1000
145/145 [==============================] - 1s 4ms/step - loss: 0.0770 -
accuracy: 0.9683 - val_loss: 0.1920 - val_accuracy: 0.9366
Epoch 261/1000
```

```
145/145 [==============================] - 1s 4ms/step - loss: 0.0759 -
accuracy: 0.9731 - val_loss: 0.1714 - val_accuracy: 0.9366
Epoch 262/1000
145/145 [==============================] - 1s 4ms/step - loss: 0.0742 -
accuracy: 0.9717 - val_loss: 0.1851 - val_accuracy: 0.9366
Epoch 263/1000
145/145 [==============================] - 1s 4ms/step - loss: 0.0735 -
accuracy: 0.9752 - val_loss: 0.1706 - val_accuracy: 0.9394
Epoch 264/1000
145/145 [==============================] - 1s 4ms/step - loss: 0.0742 -
accuracy: 0.9710 - val_loss: 0.2223 - val_accuracy: 0.9449
Epoch 265/1000
145/145 [==============================] - 1s 4ms/step - loss: 0.0752 -
accuracy: 0.9710 - val_loss: 0.1913 - val_accuracy: 0.9449
Epoch 266/1000
145/145 [==============================] - 1s 4ms/step - loss: 0.0772 -
accuracy: 0.9731 - val_loss: 0.1753 - val_accuracy: 0.9449
Epoch 267/1000
145/145 [==============================] - 0s 3ms/step - loss: 0.0780 -
accuracy: 0.9683 - val_loss: 0.1828 - val_accuracy: 0.9449
Epoch 268/1000
145/145 [==============================] - 1s 4ms/step - loss: 0.0754 -
accuracy: 0.9689 - val_loss: 0.1774 - val_accuracy: 0.9449
Epoch 269/1000
145/145 [==============================] - 1s 4ms/step - loss: 0.0781 -
accuracy: 0.9689 - val_loss: 0.1842 - val_accuracy: 0.9449
Epoch 270/1000
145/145 [==============================] - 1s 4ms/step - loss: 0.0749 -
accuracy: 0.9724 - val_loss: 0.1743 - val_accuracy: 0.9421
Epoch 271/1000
145/145 [==============================] - 1s 4ms/step - loss: 0.0752 -
accuracy: 0.9703 - val_loss: 0.2236 - val_accuracy: 0.9449
Epoch 272/1000
145/145 [==============================] - 1s 3ms/step - loss: 0.0797 -
accuracy: 0.9710 - val_loss: 0.1810 - val_accuracy: 0.9421
Epoch 273/1000
145/145 [==============================] - 1s 4ms/step - loss: 0.0746 -
accuracy: 0.9710 - val_loss: 0.2158 - val_accuracy: 0.9449
Epoch 274/1000
145/145 [==============================] - 1s 4ms/step - loss: 0.0746 -
accuracy: 0.9731 - val_loss: 0.2191 - val_accuracy: 0.9339
Epoch 275/1000
145/145 [==============================] - 1s 4ms/step - loss: 0.0774 -
accuracy: 0.9696 - val_loss: 0.2132 - val_accuracy: 0.9394
Epoch 276/1000
145/145 [==============================] - 1s 4ms/step - loss: 0.0760 -
accuracy: 0.9717 - val_loss: 0.1986 - val_accuracy: 0.9366
Epoch 277/1000
```

```
145/145 [==============================] - 1s 4ms/step - loss: 0.0772 -
accuracy: 0.9717 - val_loss: 0.1770 - val_accuracy: 0.9421
Epoch 278/1000
145/145 [==============================] - 1s 4ms/step - loss: 0.0744 -
accuracy: 0.9717 - val_loss: 0.1823 - val_accuracy: 0.9449
Epoch 279/1000
145/145 [==============================] - 1s 4ms/step - loss: 0.0736 -
accuracy: 0.9731 - val_loss: 0.1840 - val_accuracy: 0.9421
Epoch 280/1000
145/145 [==============================] - 1s 4ms/step - loss: 0.0741 -
accuracy: 0.9696 - val_loss: 0.1804 - val_accuracy: 0.9421
Epoch 281/1000
145/145 [==============================] - 1s 4ms/step - loss: 0.0753 -
accuracy: 0.9703 - val_loss: 0.1930 - val_accuracy: 0.9366
Epoch 282/1000
145/145 [==============================] - 1s 4ms/step - loss: 0.0773 -
accuracy: 0.9710 - val_loss: 0.1707 - val_accuracy: 0.9339
Epoch 283/1000
145/145 [==============================] - 1s 4ms/step - loss: 0.0744 -
accuracy: 0.9703 - val_loss: 0.1744 - val_accuracy: 0.9421
Epoch 284/1000
145/145 [==============================] - 1s 4ms/step - loss: 0.0807 -
accuracy: 0.9689 - val_loss: 0.1894 - val_accuracy: 0.9449
Epoch 285/1000
145/145 [==============================] - 1s 4ms/step - loss: 0.0741 -
accuracy: 0.9710 - val_loss: 0.2384 - val_accuracy: 0.9394
Epoch 286/1000
145/145 [==============================] - 1s 4ms/step - loss: 0.0782 -
accuracy: 0.9703 - val_loss: 0.1790 - val_accuracy: 0.9449
Epoch 287/1000
145/145 [==============================] - 1s 4ms/step - loss: 0.0762 -
accuracy: 0.9683 - val_loss: 0.1946 - val_accuracy: 0.9449
Epoch 288/1000
145/145 [==============================] - 1s 4ms/step - loss: 0.0774 -
accuracy: 0.9717 - val_loss: 0.2076 - val_accuracy: 0.9394
Epoch 289/1000
145/145 [==============================] - 1s 4ms/step - loss: 0.0768 -
accuracy: 0.9696 - val_loss: 0.1669 - val_accuracy: 0.9421
Epoch 290/1000
145/145 [==============================] - 1s 4ms/step - loss: 0.0768 -
accuracy: 0.9731 - val_loss: 0.1813 - val_accuracy: 0.9449
Epoch 291/1000
145/145 [==============================] - 1s 3ms/step - loss: 0.0759 -
accuracy: 0.9717 - val_loss: 0.1920 - val_accuracy: 0.9394
Epoch 292/1000
145/145 [==============================] - 1s 4ms/step - loss: 0.0772 -
accuracy: 0.9724 - val_loss: 0.1850 - val_accuracy: 0.9394
Epoch 293/1000
```

```
145/145 [==============================] - 1s 4ms/step - loss: 0.0767 -
accuracy: 0.9717 - val_loss: 0.1850 - val_accuracy: 0.9394
Epoch 294/1000
145/145 [==============================] - 1s 4ms/step - loss: 0.0780 -
accuracy: 0.9731 - val_loss: 0.1760 - val_accuracy: 0.9421
Epoch 295/1000
145/145 [==============================] - 1s 4ms/step - loss: 0.0796 -
accuracy: 0.9710 - val_loss: 0.1736 - val_accuracy: 0.9449
Epoch 296/1000
145/145 [==============================] - 1s 3ms/step - loss: 0.0774 -
accuracy: 0.9703 - val_loss: 0.2051 - val_accuracy: 0.9449
Epoch 297/1000
145/145 [==============================] - 1s 4ms/step - loss: 0.0759 -
accuracy: 0.9724 - val_loss: 0.1981 - val_accuracy: 0.9394
Epoch 298/1000
145/145 [==============================] - 1s 4ms/step - loss: 0.0740 -
accuracy: 0.9717 - val_loss: 0.1812 - val_accuracy: 0.9421
Epoch 299/1000
145/145 [==============================] - 1s 4ms/step - loss: 0.0768 -
accuracy: 0.9710 - val_loss: 0.1945 - val_accuracy: 0.9449
Epoch 300/1000
145/145 [==============================] - 1s 4ms/step - loss: 0.0763 -
accuracy: 0.9717 - val_loss: 0.1973 - val_accuracy: 0.9366
Epoch 301/1000
145/145 [==============================] - 1s 4ms/step - loss: 0.0785 -
accuracy: 0.9689 - val_loss: 0.1902 - val_accuracy: 0.9394
Epoch 302/1000
145/145 [==============================] - 1s 4ms/step - loss: 0.0782 -
accuracy: 0.9703 - val_loss: 0.2010 - val_accuracy: 0.9394
Epoch 303/1000
145/145 [==============================] - 1s 4ms/step - loss: 0.0747 -
accuracy: 0.9717 - val_loss: 0.1939 - val_accuracy: 0.9421
Epoch 304/1000
145/145 [==============================] - 1s 4ms/step - loss: 0.0739 -
accuracy: 0.9724 - val_loss: 0.1778 - val_accuracy: 0.9421
Epoch 305/1000
145/145 [==============================] - 1s 4ms/step - loss: 0.0760 -
accuracy: 0.9717 - val_loss: 0.2080 - val_accuracy: 0.9339
Epoch 306/1000
145/145 [==============================] - 1s 4ms/step - loss: 0.0763 -
accuracy: 0.9703 - val_loss: 0.1916 - val_accuracy: 0.9449
Epoch 307/1000
145/145 [==============================] - 1s 4ms/step - loss: 0.0738 -
accuracy: 0.9724 - val_loss: 0.2040 - val_accuracy: 0.9449
Epoch 308/1000
145/145 [==============================] - 1s 4ms/step - loss: 0.0768 -
accuracy: 0.9689 - val_loss: 0.1817 - val_accuracy: 0.9394
Epoch 309/1000
```

```
145/145 [==============================] - 1s 4ms/step - loss: 0.0768 -
accuracy: 0.9717 - val_loss: 0.1916 - val_accuracy: 0.9394
Epoch 310/1000
145/145 [==============================] - 1s 4ms/step - loss: 0.0772 -
accuracy: 0.9703 - val_loss: 0.1802 - val_accuracy: 0.9449
Epoch 311/1000
145/145 [==============================] - 1s 4ms/step - loss: 0.0743 -
accuracy: 0.9710 - val_loss: 0.1841 - val_accuracy: 0.9421
Epoch 312/1000
145/145 [==============================] - 1s 4ms/step - loss: 0.0756 -
accuracy: 0.9731 - val_loss: 0.1896 - val_accuracy: 0.9421
Epoch 313/1000
145/145 [==============================] - 1s 3ms/step - loss: 0.0743 -
accuracy: 0.9731 - val_loss: 0.1767 - val_accuracy: 0.9366
Epoch 314/1000
145/145 [==============================] - 1s 4ms/step - loss: 0.0782 -
accuracy: 0.9689 - val_loss: 0.1687 - val_accuracy: 0.9449
Epoch 315/1000
145/145 [==============================] - 1s 4ms/step - loss: 0.0760 -
accuracy: 0.9724 - val_loss: 0.1858 - val_accuracy: 0.9449
Epoch 316/1000
145/145 [==============================] - 1s 4ms/step - loss: 0.0782 -
accuracy: 0.9703 - val_loss: 0.1789 - val_accuracy: 0.9366
Epoch 317/1000
145/145 [==============================] - 1s 4ms/step - loss: 0.0769 -
accuracy: 0.9738 - val_loss: 0.1791 - val_accuracy: 0.9366
Epoch 318/1000
145/145 [==============================] - 1s 4ms/step - loss: 0.0773 -
accuracy: 0.9710 - val_loss: 0.1962 - val_accuracy: 0.9449
Epoch 319/1000
145/145 [==============================] - 1s 4ms/step - loss: 0.0755 -
accuracy: 0.9724 - val_loss: 0.2084 - val_accuracy: 0.9449
Epoch 320/1000
145/145 [==============================] - 1s 4ms/step - loss: 0.0756 -
accuracy: 0.9710 - val_loss: 0.1811 - val_accuracy: 0.9449
Epoch 321/1000
145/145 [==============================] - 1s 4ms/step - loss: 0.0756 -
accuracy: 0.9745 - val_loss: 0.2064 - val_accuracy: 0.9449
Epoch 322/1000
145/145 [==============================] - 1s 4ms/step - loss: 0.0765 -
accuracy: 0.9710 - val_loss: 0.1986 - val_accuracy: 0.9311
Epoch 323/1000
145/145 [==============================] - 1s 4ms/step - loss: 0.0800 -
accuracy: 0.9683 - val_loss: 0.1880 - val_accuracy: 0.9449
Epoch 324/1000
145/145 [==============================] - 1s 4ms/step - loss: 0.0772 -
accuracy: 0.9696 - val_loss: 0.1948 - val_accuracy: 0.9449
Epoch 325/1000
```

```
145/145 [==============================] - 1s 4ms/step - loss: 0.0769 -
accuracy: 0.9724 - val_loss: 0.1987 - val_accuracy: 0.9449
Epoch 326/1000
145/145 [==============================] - 1s 4ms/step - loss: 0.0751 -
accuracy: 0.9703 - val_loss: 0.2018 - val_accuracy: 0.9449
Epoch 327/1000
145/145 [==============================] - 1s 4ms/step - loss: 0.0781 -
accuracy: 0.9724 - val_loss: 0.1909 - val_accuracy: 0.9394
Epoch 328/1000
145/145 [==============================] - 1s 4ms/step - loss: 0.0749 -
accuracy: 0.9717 - val_loss: 0.1832 - val_accuracy: 0.9449
Epoch 329/1000
145/145 [==============================] - 1s 4ms/step - loss: 0.0755 -
accuracy: 0.9724 - val_loss: 0.1880 - val_accuracy: 0.9394
Epoch 330/1000
145/145 [==============================] - 1s 4ms/step - loss: 0.0736 -
accuracy: 0.9738 - val_loss: 0.1946 - val_accuracy: 0.9366
Epoch 331/1000
145/145 [==============================] - 1s 3ms/step - loss: 0.0798 -
accuracy: 0.9696 - val_loss: 0.1917 - val_accuracy: 0.9449
Epoch 332/1000
145/145 [==============================] - 1s 4ms/step - loss: 0.0779 -
accuracy: 0.9724 - val_loss: 0.2246 - val_accuracy: 0.9449
Epoch 333/1000
145/145 [==============================] - 1s 4ms/step - loss: 0.0797 -
accuracy: 0.9717 - val_loss: 0.1821 - val_accuracy: 0.9449
Epoch 334/1000
145/145 [==============================] - 1s 4ms/step - loss: 0.0756 -
accuracy: 0.9703 - val_loss: 0.1829 - val_accuracy: 0.9449
Epoch 335/1000
145/145 [==============================] - 1s 4ms/step - loss: 0.0748 -
accuracy: 0.9717 - val_loss: 0.2058 - val_accuracy: 0.9449
Epoch 336/1000
145/145 [==============================] - 1s 4ms/step - loss: 0.0756 -
accuracy: 0.9724 - val_loss: 0.1781 - val_accuracy: 0.9366
Epoch 337/1000
145/145 [==============================] - 1s 4ms/step - loss: 0.0769 -
accuracy: 0.9696 - val_loss: 0.2187 - val_accuracy: 0.9449
Epoch 338/1000
145/145 [==============================] - 1s 4ms/step - loss: 0.0761 -
accuracy: 0.9717 - val_loss: 0.1839 - val_accuracy: 0.9449
Epoch 339/1000
145/145 [==============================] - 1s 4ms/step - loss: 0.0753 -
accuracy: 0.9738 - val_loss: 0.1842 - val_accuracy: 0.9449
Epoch 340/1000
145/145 [==============================] - 1s 4ms/step - loss: 0.0746 -
accuracy: 0.9724 - val_loss: 0.1891 - val_accuracy: 0.9449
Epoch 341/1000
```

```
145/145 [==============================] - 1s 4ms/step - loss: 0.0764 -
accuracy: 0.9710 - val_loss: 0.1828 - val_accuracy: 0.9421
Epoch 342/1000
145/145 [==============================] - 1s 4ms/step - loss: 0.0747 -
accuracy: 0.9710 - val_loss: 0.1970 - val_accuracy: 0.9449
Epoch 343/1000
145/145 [==============================] - 1s 4ms/step - loss: 0.0771 -
accuracy: 0.9703 - val_loss: 0.1811 - val_accuracy: 0.9394
Epoch 344/1000
145/145 [==============================] - 1s 4ms/step - loss: 0.0743 -
accuracy: 0.9717 - val_loss: 0.1795 - val_accuracy: 0.9449
Epoch 345/1000
145/145 [==============================] - 1s 4ms/step - loss: 0.0750 -
accuracy: 0.9696 - val_loss: 0.1984 - val_accuracy: 0.9449
Epoch 346/1000
145/145 [==============================] - 1s 4ms/step - loss: 0.0768 -
accuracy: 0.9696 - val_loss: 0.2200 - val_accuracy: 0.9449
Epoch 347/1000
145/145 [==============================] - 1s 4ms/step - loss: 0.0740 -
accuracy: 0.9724 - val_loss: 0.2126 - val_accuracy: 0.9339
Epoch 348/1000
145/145 [==============================] - 1s 4ms/step - loss: 0.0778 -
accuracy: 0.9717 - val_loss: 0.1989 - val_accuracy: 0.9449
Epoch 349/1000
145/145 [==============================] - 1s 4ms/step - loss: 0.0753 -
accuracy: 0.9724 - val_loss: 0.1753 - val_accuracy: 0.9366
Epoch 350/1000
145/145 [==============================] - 1s 4ms/step - loss: 0.0773 -
accuracy: 0.9696 - val_loss: 0.2027 - val_accuracy: 0.9449
Epoch 351/1000
145/145 [==============================] - 1s 4ms/step - loss: 0.0749 -
accuracy: 0.9689 - val_loss: 0.2287 - val_accuracy: 0.9449
Epoch 352/1000
145/145 [==============================] - 1s 4ms/step - loss: 0.0831 -
accuracy: 0.9683 - val_loss: 0.2549 - val_accuracy: 0.9366
Epoch 353/1000
145/145 [==============================] - 1s 4ms/step - loss: 0.0855 -
accuracy: 0.9689 - val_loss: 0.1979 - val_accuracy: 0.9449
Epoch 354/1000
145/145 [==============================] - 1s 4ms/step - loss: 0.0721 -
accuracy: 0.9710 - val_loss: 0.2043 - val_accuracy: 0.9394
Epoch 355/1000
145/145 [==============================] - 1s 4ms/step - loss: 0.0715 -
accuracy: 0.9731 - val_loss: 0.1989 - val_accuracy: 0.9366
Epoch 356/1000
145/145 [==============================] - 1s 4ms/step - loss: 0.0747 -
accuracy: 0.9745 - val_loss: 0.1975 - val_accuracy: 0.9366
Epoch 357/1000
```

```
145/145 [==============================] - 1s 4ms/step - loss: 0.0740 -
accuracy: 0.9731 - val_loss: 0.2117 - val_accuracy: 0.9394
Epoch 358/1000
145/145 [==============================] - 1s 4ms/step - loss: 0.0751 -
accuracy: 0.9724 - val_loss: 0.2084 - val_accuracy: 0.9394
Epoch 359/1000
145/145 [==============================] - 1s 4ms/step - loss: 0.0743 -
accuracy: 0.9724 - val_loss: 0.2152 - val_accuracy: 0.9394
Epoch 360/1000
145/145 [==============================] - 1s 4ms/step - loss: 0.0746 -
accuracy: 0.9703 - val_loss: 0.1788 - val_accuracy: 0.9421
Epoch 361/1000
145/145 [==============================] - 1s 4ms/step - loss: 0.0768 -
accuracy: 0.9724 - val_loss: 0.1872 - val_accuracy: 0.9394
Epoch 362/1000
145/145 [==============================] - 1s 4ms/step - loss: 0.0760 -
accuracy: 0.9717 - val_loss: 0.2079 - val_accuracy: 0.9394
Epoch 363/1000
145/145 [==============================] - 1s 4ms/step - loss: 0.0769 -
accuracy: 0.9717 - val_loss: 0.1809 - val_accuracy: 0.9421
Epoch 364/1000
145/145 [==============================] - 1s 4ms/step - loss: 0.0738 -
accuracy: 0.9676 - val_loss: 0.1964 - val_accuracy: 0.9421
Epoch 365/1000
145/145 [==============================] - 1s 4ms/step - loss: 0.0756 -
accuracy: 0.9710 - val_loss: 0.1881 - val_accuracy: 0.9394
Epoch 366/1000
145/145 [==============================] - 1s 4ms/step - loss: 0.0776 -
accuracy: 0.9731 - val_loss: 0.2155 - val_accuracy: 0.9449
Epoch 367/1000
145/145 [==============================] - 1s 4ms/step - loss: 0.0755 -
accuracy: 0.9703 - val_loss: 0.1802 - val_accuracy: 0.9394
Epoch 368/1000
145/145 [==============================] - 1s 4ms/step - loss: 0.0755 -
accuracy: 0.9731 - val_loss: 0.2207 - val_accuracy: 0.9394
Epoch 369/1000
145/145 [==============================] - 1s 4ms/step - loss: 0.0749 -
accuracy: 0.9710 - val_loss: 0.1779 - val_accuracy: 0.9421
Epoch 370/1000
145/145 [==============================] - 1s 4ms/step - loss: 0.0759 -
accuracy: 0.9703 - val_loss: 0.1932 - val_accuracy: 0.9421
Epoch 371/1000
145/145 [==============================] - 1s 4ms/step - loss: 0.0751 -
accuracy: 0.9689 - val_loss: 0.1920 - val_accuracy: 0.9366
Epoch 372/1000
145/145 [==============================] - 1s 4ms/step - loss: 0.0760 -
accuracy: 0.9724 - val_loss: 0.1983 - val_accuracy: 0.9394
Epoch 373/1000
```

```
145/145 [==============================] - 1s 4ms/step - loss: 0.0780 -
accuracy: 0.9703 - val_loss: 0.1760 - val_accuracy: 0.9421
Epoch 374/1000
145/145 [==============================] - 1s 4ms/step - loss: 0.0778 -
accuracy: 0.9689 - val_loss: 0.1875 - val_accuracy: 0.9449
Epoch 375/1000
145/145 [==============================] - 1s 4ms/step - loss: 0.0748 -
accuracy: 0.9703 - val_loss: 0.2028 - val_accuracy: 0.9421
Epoch 376/1000
145/145 [==============================] - 1s 4ms/step - loss: 0.0732 -
accuracy: 0.9724 - val_loss: 0.2132 - val_accuracy: 0.9339
Epoch 377/1000
145/145 [==============================] - 1s 4ms/step - loss: 0.0783 -
accuracy: 0.9669 - val_loss: 0.1923 - val_accuracy: 0.9421
Epoch 378/1000
145/145 [==============================] - 1s 4ms/step - loss: 0.0731 -
accuracy: 0.9731 - val_loss: 0.1870 - val_accuracy: 0.9449
Epoch 379/1000
145/145 [==============================] - 1s 4ms/step - loss: 0.0764 -
accuracy: 0.9703 - val_loss: 0.1828 - val_accuracy: 0.9366
Epoch 380/1000
145/145 [==============================] - 1s 4ms/step - loss: 0.0751 -
accuracy: 0.9710 - val_loss: 0.2040 - val_accuracy: 0.9449
Epoch 381/1000
145/145 [==============================] - 1s 4ms/step - loss: 0.0777 -
accuracy: 0.9689 - val_loss: 0.2094 - val_accuracy: 0.9394
Epoch 382/1000
145/145 [==============================] - 1s 4ms/step - loss: 0.0758 -
accuracy: 0.9703 - val_loss: 0.1990 - val_accuracy: 0.9394
Epoch 383/1000
145/145 [==============================] - 1s 4ms/step - loss: 0.0772 -
accuracy: 0.9703 - val_loss: 0.1894 - val_accuracy: 0.9394
Epoch 384/1000
145/145 [==============================] - 1s 4ms/step - loss: 0.0742 -
accuracy: 0.9710 - val_loss: 0.1924 - val_accuracy: 0.9421
Epoch 385/1000
145/145 [==============================] - 1s 4ms/step - loss: 0.0764 -
accuracy: 0.9731 - val_loss: 0.1910 - val_accuracy: 0.9394
Epoch 386/1000
145/145 [==============================] - 1s 4ms/step - loss: 0.0748 -
accuracy: 0.9717 - val_loss: 0.1946 - val_accuracy: 0.9449
Epoch 387/1000
145/145 [==============================] - 1s 4ms/step - loss: 0.0756 -
accuracy: 0.9710 - val_loss: 0.1934 - val_accuracy: 0.9394
Epoch 388/1000
145/145 [==============================] - 1s 4ms/step - loss: 0.0751 -
accuracy: 0.9703 - val_loss: 0.1844 - val_accuracy: 0.9449
Epoch 389/1000
```

```
145/145 [==============================] - 1s 4ms/step - loss: 0.0747 -
accuracy: 0.9724 - val_loss: 0.1997 - val_accuracy: 0.9394
Epoch 390/1000
145/145 [==============================] - 1s 4ms/step - loss: 0.0769 -
accuracy: 0.9717 - val_loss: 0.1857 - val_accuracy: 0.9449
Epoch 391/1000
145/145 [==============================] - 1s 4ms/step - loss: 0.0757 -
accuracy: 0.9703 - val_loss: 0.1759 - val_accuracy: 0.9366
Epoch 392/1000
145/145 [==============================] - 1s 4ms/step - loss: 0.0774 -
accuracy: 0.9710 - val_loss: 0.2234 - val_accuracy: 0.9421
Epoch 393/1000
145/145 [==============================] - 1s 4ms/step - loss: 0.0728 -
accuracy: 0.9724 - val_loss: 0.1955 - val_accuracy: 0.9394
Epoch 394/1000
145/145 [==============================] - 1s 4ms/step - loss: 0.0754 -
accuracy: 0.9703 - val_loss: 0.1770 - val_accuracy: 0.9449
Epoch 395/1000
145/145 [==============================] - 1s 4ms/step - loss: 0.0762 -
accuracy: 0.9717 - val_loss: 0.1709 - val_accuracy: 0.9421
Epoch 396/1000
145/145 [==============================] - 1s 4ms/step - loss: 0.0762 -
accuracy: 0.9717 - val_loss: 0.1968 - val_accuracy: 0.9394
Epoch 397/1000
145/145 [==============================] - 1s 4ms/step - loss: 0.0747 -
accuracy: 0.9696 - val_loss: 0.1992 - val_accuracy: 0.9394
Epoch 398/1000
145/145 [==============================] - 1s 4ms/step - loss: 0.0763 -
accuracy: 0.9683 - val_loss: 0.1695 - val_accuracy: 0.9339
Epoch 399/1000
145/145 [==============================] - 1s 4ms/step - loss: 0.0759 -
accuracy: 0.9724 - val_loss: 0.1938 - val_accuracy: 0.9449
Epoch 400/1000
145/145 [==============================] - 1s 4ms/step - loss: 0.0784 -
accuracy: 0.9696 - val_loss: 0.2005 - val_accuracy: 0.9394
Epoch 401/1000
145/145 [==============================] - 1s 4ms/step - loss: 0.0781 -
accuracy: 0.9710 - val_loss: 0.1843 - val_accuracy: 0.9449
Epoch 402/1000
145/145 [==============================] - 1s 4ms/step - loss: 0.0746 -
accuracy: 0.9738 - val_loss: 0.1927 - val_accuracy: 0.9394
Epoch 403/1000
145/145 [==============================] - 1s 4ms/step - loss: 0.0746 -
accuracy: 0.9724 - val_loss: 0.2127 - val_accuracy: 0.9449
Epoch 404/1000
145/145 [==============================] - 1s 4ms/step - loss: 0.0774 -
accuracy: 0.9717 - val_loss: 0.2006 - val_accuracy: 0.9449
Epoch 405/1000
```

```
145/145 [==============================] - 1s 4ms/step - loss: 0.0759 -
accuracy: 0.9710 - val_loss: 0.1913 - val_accuracy: 0.9421
Epoch 406/1000
145/145 [==============================] - 1s 4ms/step - loss: 0.0757 -
accuracy: 0.9717 - val_loss: 0.2266 - val_accuracy: 0.9449
Epoch 407/1000
145/145 [==============================] - 1s 4ms/step - loss: 0.0765 -
accuracy: 0.9724 - val_loss: 0.1786 - val_accuracy: 0.9394
Epoch 408/1000
145/145 [==============================] - 1s 4ms/step - loss: 0.0768 -
accuracy: 0.9703 - val_loss: 0.1859 - val_accuracy: 0.9449
Epoch 409/1000
145/145 [==============================] - 1s 4ms/step - loss: 0.0737 -
accuracy: 0.9724 - val_loss: 0.1776 - val_accuracy: 0.9394
Epoch 410/1000
145/145 [==============================] - 1s 4ms/step - loss: 0.0759 -
accuracy: 0.9703 - val_loss: 0.1926 - val_accuracy: 0.9394
Epoch 411/1000
145/145 [==============================] - 1s 4ms/step - loss: 0.0745 -
accuracy: 0.9703 - val_loss: 0.2068 - val_accuracy: 0.9394
Epoch 412/1000
145/145 [==============================] - 1s 4ms/step - loss: 0.0794 -
accuracy: 0.9703 - val_loss: 0.1844 - val_accuracy: 0.9366
Epoch 413/1000
145/145 [==============================] - 1s 4ms/step - loss: 0.0750 -
accuracy: 0.9738 - val_loss: 0.2100 - val_accuracy: 0.9394
Epoch 414/1000
145/145 [==============================] - 1s 4ms/step - loss: 0.0729 -
accuracy: 0.9738 - val_loss: 0.1982 - val_accuracy: 0.9449
Epoch 415/1000
145/145 [==============================] - 1s 4ms/step - loss: 0.0736 -
accuracy: 0.9696 - val_loss: 0.1928 - val_accuracy: 0.9366
Epoch 416/1000
145/145 [==============================] - 1s 4ms/step - loss: 0.0768 -
accuracy: 0.9710 - val_loss: 0.2034 - val_accuracy: 0.9421
Epoch 417/1000
145/145 [==============================] - 1s 4ms/step - loss: 0.0750 -
accuracy: 0.9731 - val_loss: 0.1843 - val_accuracy: 0.9449
Epoch 418/1000
145/145 [==============================] - 1s 4ms/step - loss: 0.0770 -
accuracy: 0.9703 - val_loss: 0.1915 - val_accuracy: 0.9421
Epoch 419/1000
145/145 [==============================] - 1s 4ms/step - loss: 0.0760 -
accuracy: 0.9710 - val_loss: 0.2050 - val_accuracy: 0.9449
Epoch 420/1000
145/145 [==============================] - 1s 4ms/step - loss: 0.0715 -
accuracy: 0.9717 - val_loss: 0.2071 - val_accuracy: 0.9394
Epoch 421/1000
```

```
145/145 [==============================] - 1s 4ms/step - loss: 0.0758 -
accuracy: 0.9724 - val_loss: 0.1736 - val_accuracy: 0.9421
Epoch 422/1000
145/145 [==============================] - 1s 4ms/step - loss: 0.0766 -
accuracy: 0.9703 - val_loss: 0.2093 - val_accuracy: 0.9394
Epoch 423/1000
145/145 [==============================] - 1s 4ms/step - loss: 0.0748 -
accuracy: 0.9689 - val_loss: 0.2006 - val_accuracy: 0.9449
Epoch 424/1000
145/145 [==============================] - 1s 4ms/step - loss: 0.0768 -
accuracy: 0.9703 - val_loss: 0.1853 - val_accuracy: 0.9421
Epoch 425/1000
145/145 [==============================] - 1s 4ms/step - loss: 0.0748 -
accuracy: 0.9696 - val_loss: 0.1847 - val_accuracy: 0.9449
Epoch 426/1000
145/145 [==============================] - 1s 4ms/step - loss: 0.0769 -
accuracy: 0.9710 - val_loss: 0.1960 - val_accuracy: 0.9449
Epoch 427/1000
145/145 [==============================] - 1s 4ms/step - loss: 0.0762 -
accuracy: 0.9738 - val_loss: 0.1698 - val_accuracy: 0.9449
Epoch 428/1000
145/145 [==============================] - 1s 4ms/step - loss: 0.0741 -
accuracy: 0.9731 - val_loss: 0.1797 - val_accuracy: 0.9394
Epoch 429/1000
145/145 [==============================] - 1s 4ms/step - loss: 0.0748 -
accuracy: 0.9724 - val_loss: 0.1703 - val_accuracy: 0.9449
Epoch 430/1000
145/145 [==============================] - 1s 4ms/step - loss: 0.0758 -
accuracy: 0.9703 - val_loss: 0.1917 - val_accuracy: 0.9449
Epoch 431/1000
145/145 [==============================] - 1s 4ms/step - loss: 0.0736 -
accuracy: 0.9717 - val_loss: 0.1733 - val_accuracy: 0.9339
Epoch 432/1000
145/145 [==============================] - 1s 4ms/step - loss: 0.0739 -
accuracy: 0.9710 - val_loss: 0.2137 - val_accuracy: 0.9449
Epoch 433/1000
145/145 [==============================] - 1s 4ms/step - loss: 0.0731 -
accuracy: 0.9717 - val_loss: 0.2316 - val_accuracy: 0.9449
Epoch 434/1000
145/145 [==============================] - 1s 4ms/step - loss: 0.0766 -
accuracy: 0.9738 - val_loss: 0.2101 - val_accuracy: 0.9449
Epoch 435/1000
145/145 [==============================] - 1s 4ms/step - loss: 0.0740 -
accuracy: 0.9703 - val_loss: 0.1889 - val_accuracy: 0.9449
Epoch 436/1000
145/145 [==============================] - 1s 4ms/step - loss: 0.0753 -
accuracy: 0.9731 - val_loss: 0.2043 - val_accuracy: 0.9449
Epoch 437/1000
```

```
145/145 [==============================] - 1s 4ms/step - loss: 0.0772 -
accuracy: 0.9703 - val_loss: 0.1885 - val_accuracy: 0.9449
Epoch 438/1000
145/145 [==============================] - 1s 4ms/step - loss: 0.0761 -
accuracy: 0.9724 - val_loss: 0.1890 - val_accuracy: 0.9421
Epoch 439/1000
145/145 [==============================] - 1s 4ms/step - loss: 0.0742 -
accuracy: 0.9703 - val_loss: 0.1773 - val_accuracy: 0.9449
Epoch 440/1000
145/145 [==============================] - 1s 4ms/step - loss: 0.0731 -
accuracy: 0.9738 - val_loss: 0.1898 - val_accuracy: 0.9449
Epoch 441/1000
145/145 [==============================] - 1s 4ms/step - loss: 0.0762 -
accuracy: 0.9703 - val_loss: 0.1750 - val_accuracy: 0.9449
Epoch 442/1000
145/145 [==============================] - 1s 4ms/step - loss: 0.0753 -
accuracy: 0.9696 - val_loss: 0.1919 - val_accuracy: 0.9366
Epoch 443/1000
145/145 [==============================] - 1s 4ms/step - loss: 0.0737 -
accuracy: 0.9731 - val_loss: 0.2183 - val_accuracy: 0.9449
Epoch 444/1000
145/145 [==============================] - 1s 4ms/step - loss: 0.0749 -
accuracy: 0.9731 - val_loss: 0.1878 - val_accuracy: 0.9449
Epoch 445/1000
145/145 [==============================] - 1s 4ms/step - loss: 0.0772 -
accuracy: 0.9696 - val_loss: 0.2003 - val_accuracy: 0.9449
Epoch 446/1000
145/145 [==============================] - 1s 4ms/step - loss: 0.0752 -
accuracy: 0.9724 - val_loss: 0.1917 - val_accuracy: 0.9394
Epoch 447/1000
145/145 [==============================] - 1s 4ms/step - loss: 0.0709 -
accuracy: 0.9738 - val_loss: 0.2069 - val_accuracy: 0.9394
Epoch 448/1000
145/145 [==============================] - 1s 4ms/step - loss: 0.0764 -
accuracy: 0.9738 - val_loss: 0.2013 - val_accuracy: 0.9449
Epoch 449/1000
145/145 [==============================] - 1s 4ms/step - loss: 0.0732 -
accuracy: 0.9689 - val_loss: 0.2077 - val_accuracy: 0.9394
Epoch 450/1000
145/145 [==============================] - 1s 4ms/step - loss: 0.0807 -
accuracy: 0.9689 - val_loss: 0.2057 - val_accuracy: 0.9449
Epoch 451/1000
145/145 [==============================] - 1s 4ms/step - loss: 0.0761 -
accuracy: 0.9683 - val_loss: 0.2214 - val_accuracy: 0.9449
Epoch 452/1000
145/145 [==============================] - 1s 4ms/step - loss: 0.0735 -
accuracy: 0.9717 - val_loss: 0.2022 - val_accuracy: 0.9449
Epoch 453/1000
```

```
145/145 [==============================] - 1s 4ms/step - loss: 0.0760 -
accuracy: 0.9710 - val_loss: 0.1809 - val_accuracy: 0.9366
Epoch 454/1000
145/145 [==============================] - 1s 4ms/step - loss: 0.0749 -
accuracy: 0.9752 - val_loss: 0.2188 - val_accuracy: 0.9394
Epoch 455/1000
145/145 [==============================] - 1s 4ms/step - loss: 0.0756 -
accuracy: 0.9717 - val_loss: 0.1981 - val_accuracy: 0.9449
Epoch 456/1000
145/145 [==============================] - 1s 4ms/step - loss: 0.0789 -
accuracy: 0.9689 - val_loss: 0.1993 - val_accuracy: 0.9421
Epoch 457/1000
145/145 [==============================] - 1s 4ms/step - loss: 0.0762 -
accuracy: 0.9689 - val_loss: 0.1904 - val_accuracy: 0.9421
Epoch 458/1000
145/145 [==============================] - 1s 4ms/step - loss: 0.0759 -
accuracy: 0.9696 - val_loss: 0.2446 - val_accuracy: 0.9394
Epoch 459/1000
145/145 [==============================] - 1s 4ms/step - loss: 0.0726 -
accuracy: 0.9710 - val_loss: 0.1896 - val_accuracy: 0.9366
Epoch 460/1000
145/145 [==============================] - 1s 4ms/step - loss: 0.0752 -
accuracy: 0.9710 - val_loss: 0.1950 - val_accuracy: 0.9421
Epoch 461/1000
145/145 [==============================] - 1s 4ms/step - loss: 0.0752 -
accuracy: 0.9717 - val_loss: 0.1749 - val_accuracy: 0.9394
Epoch 462/1000
145/145 [==============================] - 1s 4ms/step - loss: 0.0752 -
accuracy: 0.9738 - val_loss: 0.1996 - val_accuracy: 0.9394
Epoch 463/1000
145/145 [==============================] - 1s 4ms/step - loss: 0.0771 -
accuracy: 0.9689 - val_loss: 0.1771 - val_accuracy: 0.9421
Epoch 464/1000
145/145 [==============================] - 1s 4ms/step - loss: 0.0737 -
accuracy: 0.9731 - val_loss: 0.1903 - val_accuracy: 0.9449
Epoch 465/1000
145/145 [==============================] - 1s 4ms/step - loss: 0.0734 -
accuracy: 0.9710 - val_loss: 0.1927 - val_accuracy: 0.9449
Epoch 466/1000
145/145 [==============================] - 1s 5ms/step - loss: 0.0755 -
accuracy: 0.9710 - val_loss: 0.2048 - val_accuracy: 0.9449
Epoch 467/1000
145/145 [==============================] - 1s 4ms/step - loss: 0.0769 -
accuracy: 0.9703 - val_loss: 0.1841 - val_accuracy: 0.9449
Epoch 468/1000
145/145 [==============================] - 1s 4ms/step - loss: 0.0734 -
accuracy: 0.9676 - val_loss: 0.1867 - val_accuracy: 0.9394
Epoch 469/1000
```

```
145/145 [==============================] - 1s 4ms/step - loss: 0.0745 -
accuracy: 0.9710 - val_loss: 0.1995 - val_accuracy: 0.9449
Epoch 470/1000
145/145 [==============================] - 1s 4ms/step - loss: 0.0757 -
accuracy: 0.9703 - val_loss: 0.1882 - val_accuracy: 0.9421
Epoch 471/1000
145/145 [==============================] - 1s 4ms/step - loss: 0.0767 -
accuracy: 0.9717 - val_loss: 0.1895 - val_accuracy: 0.9449
Epoch 472/1000
145/145 [==============================] - 1s 4ms/step - loss: 0.0780 -
accuracy: 0.9703 - val_loss: 0.1793 - val_accuracy: 0.9394
Epoch 473/1000
145/145 [==============================] - 1s 4ms/step - loss: 0.0738 -
accuracy: 0.9710 - val_loss: 0.2209 - val_accuracy: 0.9394
Epoch 474/1000
145/145 [==============================] - 1s 4ms/step - loss: 0.0754 -
accuracy: 0.9696 - val_loss: 0.1984 - val_accuracy: 0.9421
Epoch 475/1000
145/145 [==============================] - 1s 4ms/step - loss: 0.0770 -
accuracy: 0.9696 - val_loss: 0.1803 - val_accuracy: 0.9449
Epoch 476/1000
145/145 [==============================] - 1s 4ms/step - loss: 0.0745 -
accuracy: 0.9738 - val_loss: 0.2043 - val_accuracy: 0.9449
Epoch 477/1000
145/145 [==============================] - 1s 4ms/step - loss: 0.0769 -
accuracy: 0.9683 - val_loss: 0.1808 - val_accuracy: 0.9394
Epoch 478/1000
145/145 [==============================] - 1s 4ms/step - loss: 0.0749 -
accuracy: 0.9738 - val_loss: 0.1759 - val_accuracy: 0.9449
Epoch 479/1000
145/145 [==============================] - 1s 4ms/step - loss: 0.0753 -
accuracy: 0.9717 - val_loss: 0.1917 - val_accuracy: 0.9394
Epoch 480/1000
145/145 [==============================] - 1s 4ms/step - loss: 0.0751 -
accuracy: 0.9683 - val_loss: 0.2051 - val_accuracy: 0.9394
Epoch 481/1000
145/145 [==============================] - 1s 4ms/step - loss: 0.0764 -
accuracy: 0.9717 - val_loss: 0.1935 - val_accuracy: 0.9394
Epoch 482/1000
145/145 [==============================] - 1s 4ms/step - loss: 0.0756 -
accuracy: 0.9710 - val_loss: 0.2004 - val_accuracy: 0.9449
Epoch 483/1000
145/145 [==============================] - 1s 4ms/step - loss: 0.0748 -
accuracy: 0.9731 - val_loss: 0.2030 - val_accuracy: 0.9394
Epoch 484/1000
145/145 [==============================] - 1s 4ms/step - loss: 0.0745 -
accuracy: 0.9724 - val_loss: 0.2043 - val_accuracy: 0.9449
Epoch 485/1000
```

```
145/145 [==============================] - 1s 4ms/step - loss: 0.0754 -
accuracy: 0.9724 - val_loss: 0.1984 - val_accuracy: 0.9421
Epoch 486/1000
145/145 [==============================] - 1s 4ms/step - loss: 0.0758 -
accuracy: 0.9696 - val_loss: 0.2044 - val_accuracy: 0.9394
Epoch 487/1000
145/145 [==============================] - 1s 4ms/step - loss: 0.0781 -
accuracy: 0.9696 - val_loss: 0.2021 - val_accuracy: 0.9449
Epoch 488/1000
145/145 [==============================] - 1s 4ms/step - loss: 0.0745 -
accuracy: 0.9703 - val_loss: 0.2126 - val_accuracy: 0.9449
Epoch 489/1000
145/145 [==============================] - 1s 4ms/step - loss: 0.0734 -
accuracy: 0.9717 - val_loss: 0.2122 - val_accuracy: 0.9394
Epoch 490/1000
145/145 [==============================] - 1s 4ms/step - loss: 0.0738 -
accuracy: 0.9703 - val_loss: 0.2027 - val_accuracy: 0.9394
Epoch 491/1000
145/145 [==============================] - 1s 4ms/step - loss: 0.0748 -
accuracy: 0.9738 - val_loss: 0.1869 - val_accuracy: 0.9366
Epoch 492/1000
145/145 [==============================] - 1s 4ms/step - loss: 0.0755 -
accuracy: 0.9703 - val_loss: 0.1875 - val_accuracy: 0.9449
Epoch 493/1000
145/145 [==============================] - 1s 4ms/step - loss: 0.0744 -
accuracy: 0.9703 - val_loss: 0.1910 - val_accuracy: 0.9421
Epoch 494/1000
145/145 [==============================] - 1s 4ms/step - loss: 0.0773 -
accuracy: 0.9696 - val_loss: 0.1956 - val_accuracy: 0.9421
Epoch 495/1000
145/145 [==============================] - 1s 4ms/step - loss: 0.0745 -
accuracy: 0.9731 - val_loss: 0.2083 - val_accuracy: 0.9449
Epoch 496/1000
145/145 [==============================] - 1s 4ms/step - loss: 0.0735 -
accuracy: 0.9710 - val_loss: 0.1796 - val_accuracy: 0.9477
Epoch 497/1000
145/145 [==============================] - 1s 4ms/step - loss: 0.0742 -
accuracy: 0.9724 - val_loss: 0.1961 - val_accuracy: 0.9394
Epoch 498/1000
145/145 [==============================] - 1s 4ms/step - loss: 0.0763 -
accuracy: 0.9717 - val_loss: 0.2127 - val_accuracy: 0.9394
Epoch 499/1000
145/145 [==============================] - 1s 4ms/step - loss: 0.0734 -
accuracy: 0.9676 - val_loss: 0.1858 - val_accuracy: 0.9366
Epoch 500/1000
145/145 [==============================] - 1s 4ms/step - loss: 0.0768 -
accuracy: 0.9717 - val_loss: 0.1864 - val_accuracy: 0.9421
Epoch 501/1000
```

```
145/145 [==============================] - 1s 4ms/step - loss: 0.0751 -
accuracy: 0.9731 - val_loss: 0.1864 - val_accuracy: 0.9394
Epoch 502/1000
145/145 [==============================] - 1s 4ms/step - loss: 0.0773 -
accuracy: 0.9703 - val_loss: 0.2001 - val_accuracy: 0.9394
Epoch 503/1000
145/145 [==============================] - 1s 4ms/step - loss: 0.0742 -
accuracy: 0.9717 - val_loss: 0.1885 - val_accuracy: 0.9421
Epoch 504/1000
145/145 [==============================] - 1s 4ms/step - loss: 0.0732 -
accuracy: 0.9703 - val_loss: 0.2120 - val_accuracy: 0.9366
Epoch 505/1000
145/145 [==============================] - 1s 4ms/step - loss: 0.0758 -
accuracy: 0.9703 - val_loss: 0.1806 - val_accuracy: 0.9421
Epoch 506/1000
145/145 [==============================] - 1s 4ms/step - loss: 0.0754 -
accuracy: 0.9717 - val_loss: 0.1883 - val_accuracy: 0.9449
Epoch 507/1000
145/145 [==============================] - 1s 4ms/step - loss: 0.0752 -
accuracy: 0.9689 - val_loss: 0.2162 - val_accuracy: 0.9449
Epoch 508/1000
145/145 [==============================] - 1s 4ms/step - loss: 0.0748 -
accuracy: 0.9703 - val_loss: 0.1964 - val_accuracy: 0.9449
Epoch 509/1000
145/145 [==============================] - 1s 4ms/step - loss: 0.0749 -
accuracy: 0.9703 - val_loss: 0.2164 - val_accuracy: 0.9394
Epoch 510/1000
145/145 [==============================] - 1s 4ms/step - loss: 0.0758 -
accuracy: 0.9738 - val_loss: 0.2389 - val_accuracy: 0.9449
Epoch 511/1000
145/145 [==============================] - 1s 4ms/step - loss: 0.0750 -
accuracy: 0.9710 - val_loss: 0.1913 - val_accuracy: 0.9421
Epoch 512/1000
145/145 [==============================] - 1s 4ms/step - loss: 0.0731 -
accuracy: 0.9738 - val_loss: 0.1918 - val_accuracy: 0.9449
Epoch 513/1000
145/145 [==============================] - 1s 4ms/step - loss: 0.0755 -
accuracy: 0.9731 - val_loss: 0.2089 - val_accuracy: 0.9449
Epoch 514/1000
145/145 [==============================] - 1s 4ms/step - loss: 0.0768 -
accuracy: 0.9731 - val_loss: 0.2054 - val_accuracy: 0.9311
Epoch 515/1000
145/145 [==============================] - 1s 4ms/step - loss: 0.0781 -
accuracy: 0.9703 - val_loss: 0.1727 - val_accuracy: 0.9421
Epoch 516/1000
145/145 [==============================] - 1s 4ms/step - loss: 0.0775 -
accuracy: 0.9717 - val_loss: 0.1856 - val_accuracy: 0.9449
Epoch 517/1000
```

```
145/145 [==============================] - 1s 4ms/step - loss: 0.0736 -
accuracy: 0.9717 - val_loss: 0.1778 - val_accuracy: 0.9449
Epoch 518/1000
145/145 [==============================] - 1s 4ms/step - loss: 0.0730 -
accuracy: 0.9703 - val_loss: 0.2014 - val_accuracy: 0.9449
Epoch 519/1000
145/145 [==============================] - 1s 4ms/step - loss: 0.0773 -
accuracy: 0.9696 - val_loss: 0.1772 - val_accuracy: 0.9394
Epoch 520/1000
145/145 [==============================] - 1s 4ms/step - loss: 0.0751 -
accuracy: 0.9738 - val_loss: 0.1868 - val_accuracy: 0.9394
Epoch 521/1000
145/145 [==============================] - 1s 4ms/step - loss: 0.0730 -
accuracy: 0.9703 - val_loss: 0.2020 - val_accuracy: 0.9449
Epoch 522/1000
145/145 [==============================] - 1s 4ms/step - loss: 0.0750 -
accuracy: 0.9731 - val_loss: 0.2079 - val_accuracy: 0.9421
Epoch 523/1000
145/145 [==============================] - 1s 4ms/step - loss: 0.0765 -
accuracy: 0.9703 - val_loss: 0.1849 - val_accuracy: 0.9421
Epoch 524/1000
145/145 [==============================] - 1s 4ms/step - loss: 0.0797 -
accuracy: 0.9710 - val_loss: 0.2034 - val_accuracy: 0.9449
Epoch 525/1000
145/145 [==============================] - 1s 4ms/step - loss: 0.0723 -
accuracy: 0.9758 - val_loss: 0.2049 - val_accuracy: 0.9394
Epoch 526/1000
145/145 [==============================] - 1s 4ms/step - loss: 0.0756 -
accuracy: 0.9696 - val_loss: 0.2071 - val_accuracy: 0.9394
Epoch 527/1000
145/145 [==============================] - 1s 4ms/step - loss: 0.0729 -
accuracy: 0.9703 - val_loss: 0.1918 - val_accuracy: 0.9366
Epoch 528/1000
145/145 [==============================] - 1s 4ms/step - loss: 0.0749 -
accuracy: 0.9731 - val_loss: 0.1983 - val_accuracy: 0.9449
Epoch 529/1000
145/145 [==============================] - 1s 4ms/step - loss: 0.0764 -
accuracy: 0.9703 - val_loss: 0.1949 - val_accuracy: 0.9366
Epoch 530/1000
145/145 [==============================] - 1s 4ms/step - loss: 0.0775 -
accuracy: 0.9717 - val_loss: 0.2039 - val_accuracy: 0.9449
Epoch 531/1000
145/145 [==============================] - 1s 4ms/step - loss: 0.0738 -
accuracy: 0.9696 - val_loss: 0.1975 - val_accuracy: 0.9366
Epoch 532/1000
145/145 [==============================] - 1s 4ms/step - loss: 0.0748 -
accuracy: 0.9717 - val_loss: 0.2140 - val_accuracy: 0.9449
Epoch 533/1000
```

```
145/145 [==============================] - 1s 4ms/step - loss: 0.0748 -
accuracy: 0.9724 - val_loss: 0.2074 - val_accuracy: 0.9449
Epoch 534/1000
145/145 [==============================] - 1s 4ms/step - loss: 0.0749 -
accuracy: 0.9710 - val_loss: 0.1929 - val_accuracy: 0.9366
Epoch 535/1000
145/145 [==============================] - 1s 4ms/step - loss: 0.0747 -
accuracy: 0.9717 - val_loss: 0.1784 - val_accuracy: 0.9421
Epoch 536/1000
145/145 [==============================] - 1s 4ms/step - loss: 0.0729 -
accuracy: 0.9745 - val_loss: 0.2030 - val_accuracy: 0.9366
Epoch 537/1000
145/145 [==============================] - 1s 4ms/step - loss: 0.0748 -
accuracy: 0.9717 - val_loss: 0.1807 - val_accuracy: 0.9449
Epoch 538/1000
145/145 [==============================] - 1s 4ms/step - loss: 0.0753 -
accuracy: 0.9724 - val_loss: 0.2279 - val_accuracy: 0.9394
Epoch 539/1000
145/145 [==============================] - 1s 4ms/step - loss: 0.0815 -
accuracy: 0.9696 - val_loss: 0.1860 - val_accuracy: 0.9449
Epoch 540/1000
145/145 [==============================] - 1s 4ms/step - loss: 0.0733 -
accuracy: 0.9724 - val_loss: 0.2102 - val_accuracy: 0.9339
Epoch 541/1000
145/145 [==============================] - 1s 4ms/step - loss: 0.0740 -
accuracy: 0.9717 - val_loss: 0.2040 - val_accuracy: 0.9394
Epoch 542/1000
145/145 [==============================] - 1s 4ms/step - loss: 0.0759 -
accuracy: 0.9703 - val_loss: 0.1917 - val_accuracy: 0.9449
Epoch 543/1000
145/145 [==============================] - 1s 4ms/step - loss: 0.0738 -
accuracy: 0.9710 - val_loss: 0.1899 - val_accuracy: 0.9421
Epoch 544/1000
145/145 [==============================] - 1s 4ms/step - loss: 0.0751 -
accuracy: 0.9731 - val_loss: 0.1784 - val_accuracy: 0.9394
Epoch 545/1000
145/145 [==============================] - 1s 4ms/step - loss: 0.0749 -
accuracy: 0.9703 - val_loss: 0.1958 - val_accuracy: 0.9394
Epoch 546/1000
145/145 [==============================] - 1s 4ms/step - loss: 0.0749 -
accuracy: 0.9676 - val_loss: 0.2298 - val_accuracy: 0.9394
Epoch 547/1000
145/145 [==============================] - 1s 4ms/step - loss: 0.0766 -
accuracy: 0.9738 - val_loss: 0.1948 - val_accuracy: 0.9449
Epoch 548/1000
145/145 [==============================] - 1s 4ms/step - loss: 0.0737 -
accuracy: 0.9717 - val_loss: 0.2063 - val_accuracy: 0.9449
Epoch 549/1000
```

```
145/145 [==============================] - 1s 4ms/step - loss: 0.0737 -
accuracy: 0.9710 - val_loss: 0.1911 - val_accuracy: 0.9421
Epoch 550/1000
145/145 [==============================] - 1s 4ms/step - loss: 0.0736 -
accuracy: 0.9703 - val_loss: 0.1923 - val_accuracy: 0.9421
Epoch 551/1000
145/145 [==============================] - 1s 4ms/step - loss: 0.0764 -
accuracy: 0.9710 - val_loss: 0.2214 - val_accuracy: 0.9449
Epoch 552/1000
145/145 [==============================] - 1s 4ms/step - loss: 0.0738 -
accuracy: 0.9717 - val_loss: 0.1926 - val_accuracy: 0.9421
Epoch 553/1000
145/145 [==============================] - 1s 4ms/step - loss: 0.0741 -
accuracy: 0.9724 - val_loss: 0.2101 - val_accuracy: 0.9449
Epoch 554/1000
145/145 [==============================] - 1s 4ms/step - loss: 0.0758 -
accuracy: 0.9703 - val_loss: 0.2038 - val_accuracy: 0.9449
Epoch 555/1000
145/145 [==============================] - 1s 4ms/step - loss: 0.0784 -
accuracy: 0.9703 - val_loss: 0.2018 - val_accuracy: 0.9394
Epoch 556/1000
145/145 [==============================] - 1s 4ms/step - loss: 0.0750 -
accuracy: 0.9724 - val_loss: 0.1994 - val_accuracy: 0.9394
Epoch 557/1000
145/145 [==============================] - 1s 4ms/step - loss: 0.0754 -
accuracy: 0.9689 - val_loss: 0.1943 - val_accuracy: 0.9449
Epoch 558/1000
145/145 [==============================] - 1s 4ms/step - loss: 0.0746 -
accuracy: 0.9710 - val_loss: 0.2028 - val_accuracy: 0.9366
Epoch 559/1000
145/145 [==============================] - 1s 4ms/step - loss: 0.0766 -
accuracy: 0.9710 - val_loss: 0.1965 - val_accuracy: 0.9449
Epoch 560/1000
145/145 [==============================] - 1s 4ms/step - loss: 0.0738 -
accuracy: 0.9724 - val_loss: 0.1920 - val_accuracy: 0.9421
Epoch 561/1000
145/145 [==============================] - 1s 4ms/step - loss: 0.0748 -
accuracy: 0.9717 - val_loss: 0.2031 - val_accuracy: 0.9394
Epoch 562/1000
145/145 [==============================] - 1s 4ms/step - loss: 0.0738 -
accuracy: 0.9724 - val_loss: 0.1810 - val_accuracy: 0.9421
Epoch 563/1000
145/145 [==============================] - 1s 4ms/step - loss: 0.0747 -
accuracy: 0.9696 - val_loss: 0.2021 - val_accuracy: 0.9449
Epoch 564/1000
145/145 [==============================] - 1s 4ms/step - loss: 0.0729 -
accuracy: 0.9731 - val_loss: 0.2277 - val_accuracy: 0.9449
Epoch 565/1000
```

```
145/145 [==============================] - 1s 4ms/step - loss: 0.0737 -
accuracy: 0.9724 - val_loss: 0.1891 - val_accuracy: 0.9366
Epoch 566/1000
145/145 [==============================] - 1s 4ms/step - loss: 0.0733 -
accuracy: 0.9724 - val_loss: 0.1843 - val_accuracy: 0.9366
Epoch 567/1000
145/145 [==============================] - 1s 4ms/step - loss: 0.0754 -
accuracy: 0.9710 - val_loss: 0.2045 - val_accuracy: 0.9366
Epoch 568/1000
145/145 [==============================] - 1s 4ms/step - loss: 0.0738 -
accuracy: 0.9717 - val_loss: 0.2146 - val_accuracy: 0.9394
Epoch 569/1000
145/145 [==============================] - 1s 5ms/step - loss: 0.0740 -
accuracy: 0.9703 - val_loss: 0.1908 - val_accuracy: 0.9366
Epoch 570/1000
145/145 [==============================] - 1s 4ms/step - loss: 0.0807 -
accuracy: 0.9689 - val_loss: 0.2196 - val_accuracy: 0.9311
Epoch 571/1000
145/145 [==============================] - 1s 4ms/step - loss: 0.0809 -
accuracy: 0.9696 - val_loss: 0.1991 - val_accuracy: 0.9339
Epoch 572/1000
145/145 [==============================] - 1s 4ms/step - loss: 0.0762 -
accuracy: 0.9710 - val_loss: 0.2175 - val_accuracy: 0.9394
Epoch 573/1000
145/145 [==============================] - 1s 5ms/step - loss: 0.0728 -
accuracy: 0.9731 - val_loss: 0.2026 - val_accuracy: 0.9449
Epoch 574/1000
145/145 [==============================] - 1s 5ms/step - loss: 0.0714 -
accuracy: 0.9710 - val_loss: 0.1984 - val_accuracy: 0.9421
Epoch 575/1000
145/145 [==============================] - 1s 4ms/step - loss: 0.0733 -
accuracy: 0.9703 - val_loss: 0.1825 - val_accuracy: 0.9421
Epoch 576/1000
145/145 [==============================] - 1s 4ms/step - loss: 0.0737 -
accuracy: 0.9717 - val_loss: 0.1938 - val_accuracy: 0.9449
Epoch 577/1000
145/145 [==============================] - 1s 4ms/step - loss: 0.0745 -
accuracy: 0.9717 - val_loss: 0.1879 - val_accuracy: 0.9449
Epoch 578/1000
145/145 [==============================] - 1s 4ms/step - loss: 0.0729 -
accuracy: 0.9724 - val_loss: 0.1976 - val_accuracy: 0.9394
Epoch 579/1000
145/145 [==============================] - 1s 4ms/step - loss: 0.0752 -
accuracy: 0.9731 - val_loss: 0.1931 - val_accuracy: 0.9366
Epoch 580/1000
145/145 [==============================] - 1s 4ms/step - loss: 0.0710 -
accuracy: 0.9696 - val_loss: 0.2206 - val_accuracy: 0.9394
Epoch 581/1000
```

```
145/145 [==============================] - 1s 4ms/step - loss: 0.0738 -
accuracy: 0.9738 - val_loss: 0.2137 - val_accuracy: 0.9449
Epoch 582/1000
145/145 [==============================] - 1s 4ms/step - loss: 0.0759 -
accuracy: 0.9689 - val_loss: 0.1937 - val_accuracy: 0.9449
Epoch 583/1000
145/145 [==============================] - 1s 4ms/step - loss: 0.0741 -
accuracy: 0.9717 - val_loss: 0.1973 - val_accuracy: 0.9449
Epoch 584/1000
145/145 [==============================] - 1s 4ms/step - loss: 0.0738 -
accuracy: 0.9717 - val_loss: 0.1988 - val_accuracy: 0.9449
Epoch 585/1000
145/145 [==============================] - 1s 4ms/step - loss: 0.0731 -
accuracy: 0.9710 - val_loss: 0.2089 - val_accuracy: 0.9394
Epoch 586/1000
145/145 [==============================] - 1s 4ms/step - loss: 0.0716 -
accuracy: 0.9710 - val_loss: 0.2114 - val_accuracy: 0.9394
Epoch 587/1000
145/145 [==============================] - 1s 4ms/step - loss: 0.0748 -
accuracy: 0.9696 - val_loss: 0.1962 - val_accuracy: 0.9394
Epoch 588/1000
145/145 [==============================] - 1s 4ms/step - loss: 0.0765 -
accuracy: 0.9710 - val_loss: 0.1886 - val_accuracy: 0.9421
Epoch 589/1000
145/145 [==============================] - 1s 4ms/step - loss: 0.0760 -
accuracy: 0.9724 - val_loss: 0.1993 - val_accuracy: 0.9421
Epoch 590/1000
145/145 [==============================] - 1s 4ms/step - loss: 0.0734 -
accuracy: 0.9696 - val_loss: 0.1971 - val_accuracy: 0.9394
Epoch 591/1000
145/145 [==============================] - 1s 4ms/step - loss: 0.0813 -
accuracy: 0.9683 - val_loss: 0.1830 - val_accuracy: 0.9421
Epoch 592/1000
145/145 [==============================] - 1s 4ms/step - loss: 0.0750 -
accuracy: 0.9717 - val_loss: 0.2034 - val_accuracy: 0.9449
Epoch 593/1000
145/145 [==============================] - 1s 4ms/step - loss: 0.0726 -
accuracy: 0.9724 - val_loss: 0.2032 - val_accuracy: 0.9394
Epoch 594/1000
145/145 [==============================] - 1s 4ms/step - loss: 0.0772 -
accuracy: 0.9683 - val_loss: 0.1880 - val_accuracy: 0.9339
Epoch 595/1000
145/145 [==============================] - 1s 4ms/step - loss: 0.0758 -
accuracy: 0.9703 - val_loss: 0.2191 - val_accuracy: 0.9394
Epoch 596/1000
145/145 [==============================] - 1s 4ms/step - loss: 0.0751 -
accuracy: 0.9731 - val_loss: 0.1885 - val_accuracy: 0.9366
Epoch 597/1000
```

```
145/145 [==============================] - 1s 4ms/step - loss: 0.0763 -
accuracy: 0.9703 - val_loss: 0.1959 - val_accuracy: 0.9449
Epoch 598/1000
145/145 [==============================] - 1s 4ms/step - loss: 0.0740 -
accuracy: 0.9724 - val_loss: 0.2008 - val_accuracy: 0.9449
Epoch 599/1000
145/145 [==============================] - 1s 4ms/step - loss: 0.0723 -
accuracy: 0.9696 - val_loss: 0.2174 - val_accuracy: 0.9421
Epoch 600/1000
145/145 [==============================] - 1s 4ms/step - loss: 0.0751 -
accuracy: 0.9689 - val_loss: 0.2170 - val_accuracy: 0.9394
Epoch 601/1000
145/145 [==============================] - 1s 4ms/step - loss: 0.0763 -
accuracy: 0.9717 - val_loss: 0.1790 - val_accuracy: 0.9449
Epoch 602/1000
145/145 [==============================] - 1s 4ms/step - loss: 0.0756 -
accuracy: 0.9676 - val_loss: 0.2009 - val_accuracy: 0.9449
Epoch 603/1000
145/145 [==============================] - 1s 4ms/step - loss: 0.0747 -
accuracy: 0.9703 - val_loss: 0.1987 - val_accuracy: 0.9366
Epoch 604/1000
145/145 [==============================] - 1s 4ms/step - loss: 0.0724 -
accuracy: 0.9717 - val_loss: 0.1874 - val_accuracy: 0.9394
Epoch 605/1000
145/145 [==============================] - 1s 4ms/step - loss: 0.0764 -
accuracy: 0.9717 - val_loss: 0.1997 - val_accuracy: 0.9449
Epoch 606/1000
145/145 [==============================] - 1s 4ms/step - loss: 0.0749 -
accuracy: 0.9710 - val_loss: 0.2356 - val_accuracy: 0.9449
Epoch 607/1000
145/145 [==============================] - 1s 4ms/step - loss: 0.0740 -
accuracy: 0.9703 - val_loss: 0.2112 - val_accuracy: 0.9449
Epoch 608/1000
145/145 [==============================] - 1s 4ms/step - loss: 0.0731 -
accuracy: 0.9724 - val_loss: 0.1923 - val_accuracy: 0.9421
Epoch 609/1000
145/145 [==============================] - 1s 4ms/step - loss: 0.0744 -
accuracy: 0.9703 - val_loss: 0.2036 - val_accuracy: 0.9394
Epoch 610/1000
145/145 [==============================] - 1s 4ms/step - loss: 0.0749 -
accuracy: 0.9703 - val_loss: 0.2028 - val_accuracy: 0.9394
Epoch 611/1000
145/145 [==============================] - 1s 4ms/step - loss: 0.0778 -
accuracy: 0.9696 - val_loss: 0.2123 - val_accuracy: 0.9449
Epoch 612/1000
145/145 [==============================] - 1s 4ms/step - loss: 0.0759 -
accuracy: 0.9703 - val_loss: 0.1932 - val_accuracy: 0.9421
Epoch 613/1000
```

```
145/145 [==============================] - 1s 5ms/step - loss: 0.0752 -
accuracy: 0.9717 - val_loss: 0.1997 - val_accuracy: 0.9449
Epoch 614/1000
145/145 [==============================] - 1s 4ms/step - loss: 0.0750 -
accuracy: 0.9717 - val_loss: 0.2257 - val_accuracy: 0.9394
Epoch 615/1000
145/145 [==============================] - 1s 4ms/step - loss: 0.0737 -
accuracy: 0.9731 - val_loss: 0.2205 - val_accuracy: 0.9421
Epoch 616/1000
145/145 [==============================] - 1s 4ms/step - loss: 0.0757 -
accuracy: 0.9724 - val_loss: 0.2009 - val_accuracy: 0.9449
Epoch 617/1000
145/145 [==============================] - 1s 4ms/step - loss: 0.0748 -
accuracy: 0.9717 - val_loss: 0.2164 - val_accuracy: 0.9394
Epoch 618/1000
145/145 [==============================] - 1s 4ms/step - loss: 0.0750 -
accuracy: 0.9710 - val_loss: 0.2241 - val_accuracy: 0.9449
Epoch 619/1000
145/145 [==============================] - 1s 4ms/step - loss: 0.0767 -
accuracy: 0.9710 - val_loss: 0.2317 - val_accuracy: 0.9394
Epoch 620/1000
145/145 [==============================] - 1s 4ms/step - loss: 0.0749 -
accuracy: 0.9703 - val_loss: 0.2012 - val_accuracy: 0.9366
Epoch 621/1000
145/145 [==============================] - 1s 4ms/step - loss: 0.0762 -
accuracy: 0.9710 - val_loss: 0.1913 - val_accuracy: 0.9421
Epoch 622/1000
145/145 [==============================] - 1s 4ms/step - loss: 0.0749 -
accuracy: 0.9724 - val_loss: 0.1975 - val_accuracy: 0.9366
Epoch 623/1000
145/145 [==============================] - 1s 4ms/step - loss: 0.0750 -
accuracy: 0.9717 - val_loss: 0.2020 - val_accuracy: 0.9449
Epoch 624/1000
145/145 [==============================] - 1s 4ms/step - loss: 0.0733 -
accuracy: 0.9717 - val_loss: 0.2186 - val_accuracy: 0.9394
Epoch 625/1000
145/145 [==============================] - 1s 4ms/step - loss: 0.0784 -
accuracy: 0.9710 - val_loss: 0.2166 - val_accuracy: 0.9449
Epoch 626/1000
145/145 [==============================] - 1s 4ms/step - loss: 0.0723 -
accuracy: 0.9724 - val_loss: 0.1911 - val_accuracy: 0.9449
Epoch 627/1000
145/145 [==============================] - 1s 5ms/step - loss: 0.0794 -
accuracy: 0.9689 - val_loss: 0.2210 - val_accuracy: 0.9449
Epoch 628/1000
145/145 [==============================] - 1s 4ms/step - loss: 0.0745 -
accuracy: 0.9710 - val_loss: 0.2101 - val_accuracy: 0.9421
Epoch 629/1000
```

```
145/145 [==============================] - 1s 4ms/step - loss: 0.0748 -
accuracy: 0.9724 - val_loss: 0.2151 - val_accuracy: 0.9449
Epoch 630/1000
145/145 [==============================] - 1s 4ms/step - loss: 0.0745 -
accuracy: 0.9731 - val_loss: 0.2237 - val_accuracy: 0.9394
Epoch 631/1000
145/145 [==============================] - 1s 4ms/step - loss: 0.0767 -
accuracy: 0.9683 - val_loss: 0.1981 - val_accuracy: 0.9449
Epoch 632/1000
145/145 [==============================] - 1s 4ms/step - loss: 0.0737 -
accuracy: 0.9724 - val_loss: 0.1927 - val_accuracy: 0.9449
Epoch 633/1000
145/145 [==============================] - 1s 5ms/step - loss: 0.0760 -
accuracy: 0.9724 - val_loss: 0.2197 - val_accuracy: 0.9449
Epoch 634/1000
145/145 [==============================] - 1s 4ms/step - loss: 0.0749 -
accuracy: 0.9717 - val_loss: 0.1868 - val_accuracy: 0.9394
Epoch 635/1000
145/145 [==============================] - 1s 4ms/step - loss: 0.0737 -
accuracy: 0.9703 - val_loss: 0.1817 - val_accuracy: 0.9394
Epoch 636/1000
145/145 [==============================] - 1s 4ms/step - loss: 0.0736 -
accuracy: 0.9717 - val_loss: 0.1840 - val_accuracy: 0.9449
Epoch 637/1000
145/145 [==============================] - 1s 4ms/step - loss: 0.0736 -
accuracy: 0.9724 - val_loss: 0.2095 - val_accuracy: 0.9421
Epoch 638/1000
145/145 [==============================] - 1s 4ms/step - loss: 0.0779 -
accuracy: 0.9703 - val_loss: 0.1947 - val_accuracy: 0.9449
Epoch 639/1000
145/145 [==============================] - 1s 4ms/step - loss: 0.0733 -
accuracy: 0.9745 - val_loss: 0.1977 - val_accuracy: 0.9449
Epoch 640/1000
145/145 [==============================] - 1s 4ms/step - loss: 0.0790 -
accuracy: 0.9662 - val_loss: 0.2043 - val_accuracy: 0.9339
Epoch 641/1000
145/145 [==============================] - 1s 4ms/step - loss: 0.0742 -
accuracy: 0.9738 - val_loss: 0.2055 - val_accuracy: 0.9449
Epoch 642/1000
145/145 [==============================] - 1s 4ms/step - loss: 0.0771 -
accuracy: 0.9696 - val_loss: 0.2127 - val_accuracy: 0.9421
Epoch 643/1000
145/145 [==============================] - 1s 5ms/step - loss: 0.0745 -
accuracy: 0.9731 - val_loss: 0.2268 - val_accuracy: 0.9394
Epoch 644/1000
145/145 [==============================] - 1s 4ms/step - loss: 0.0731 -
accuracy: 0.9696 - val_loss: 0.2057 - val_accuracy: 0.9394
Epoch 645/1000
```

```
145/145 [==============================] - 1s 4ms/step - loss: 0.0730 -
accuracy: 0.9731 - val_loss: 0.2091 - val_accuracy: 0.9366
Epoch 646/1000
145/145 [==============================] - 1s 4ms/step - loss: 0.0748 -
accuracy: 0.9731 - val_loss: 0.1905 - val_accuracy: 0.9449
Epoch 647/1000
145/145 [==============================] - 1s 4ms/step - loss: 0.0733 -
accuracy: 0.9731 - val_loss: 0.1943 - val_accuracy: 0.9449
Epoch 648/1000
145/145 [==============================] - 1s 4ms/step - loss: 0.0741 -
accuracy: 0.9724 - val_loss: 0.1887 - val_accuracy: 0.9449
Epoch 649/1000
145/145 [==============================] - 1s 4ms/step - loss: 0.0752 -
accuracy: 0.9717 - val_loss: 0.2028 - val_accuracy: 0.9449
Epoch 650/1000
145/145 [==============================] - 1s 4ms/step - loss: 0.0756 -
accuracy: 0.9710 - val_loss: 0.2007 - val_accuracy: 0.9449
Epoch 651/1000
145/145 [==============================] - 1s 4ms/step - loss: 0.0739 -
accuracy: 0.9724 - val_loss: 0.2133 - val_accuracy: 0.9394
Epoch 652/1000
145/145 [==============================] - 1s 4ms/step - loss: 0.0741 -
accuracy: 0.9717 - val_loss: 0.2078 - val_accuracy: 0.9284
Epoch 653/1000
145/145 [==============================] - 1s 4ms/step - loss: 0.0739 -
accuracy: 0.9717 - val_loss: 0.2183 - val_accuracy: 0.9394
Epoch 654/1000
145/145 [==============================] - 1s 4ms/step - loss: 0.0736 -
accuracy: 0.9710 - val_loss: 0.1976 - val_accuracy: 0.9394
Epoch 655/1000
145/145 [==============================] - 1s 4ms/step - loss: 0.0749 -
accuracy: 0.9717 - val_loss: 0.1972 - val_accuracy: 0.9394
Epoch 656/1000
145/145 [==============================] - 1s 4ms/step - loss: 0.0724 -
accuracy: 0.9717 - val_loss: 0.2107 - val_accuracy: 0.9449
Epoch 657/1000
145/145 [==============================] - 1s 4ms/step - loss: 0.0704 -
accuracy: 0.9724 - val_loss: 0.2179 - val_accuracy: 0.9449
Epoch 658/1000
145/145 [==============================] - 1s 4ms/step - loss: 0.0757 -
accuracy: 0.9710 - val_loss: 0.2017 - val_accuracy: 0.9449
Epoch 659/1000
145/145 [==============================] - 1s 4ms/step - loss: 0.0755 -
accuracy: 0.9731 - val_loss: 0.2067 - val_accuracy: 0.9394
Epoch 660/1000
145/145 [==============================] - 1s 4ms/step - loss: 0.0766 -
accuracy: 0.9703 - val_loss: 0.2297 - val_accuracy: 0.9394
Epoch 661/1000
```

```
145/145 [==============================] - 1s 4ms/step - loss: 0.0733 -
accuracy: 0.9717 - val_loss: 0.1943 - val_accuracy: 0.9394
Epoch 662/1000
145/145 [==============================] - 1s 4ms/step - loss: 0.0751 -
accuracy: 0.9683 - val_loss: 0.2069 - val_accuracy: 0.9449
Epoch 663/1000
145/145 [==============================] - 1s 4ms/step - loss: 0.0740 -
accuracy: 0.9717 - val_loss: 0.2011 - val_accuracy: 0.9394
Epoch 664/1000
145/145 [==============================] - 1s 4ms/step - loss: 0.0747 -
accuracy: 0.9703 - val_loss: 0.1972 - val_accuracy: 0.9449
Epoch 665/1000
145/145 [==============================] - 1s 4ms/step - loss: 0.0747 -
accuracy: 0.9717 - val_loss: 0.1889 - val_accuracy: 0.9449
Epoch 666/1000
145/145 [==============================] - 1s 4ms/step - loss: 0.0725 -
accuracy: 0.9731 - val_loss: 0.2017 - val_accuracy: 0.9449
Epoch 667/1000
145/145 [==============================] - 1s 4ms/step - loss: 0.0732 -
accuracy: 0.9717 - val_loss: 0.2051 - val_accuracy: 0.9449
Epoch 668/1000
145/145 [==============================] - 1s 4ms/step - loss: 0.0772 -
accuracy: 0.9724 - val_loss: 0.1911 - val_accuracy: 0.9421
Epoch 669/1000
145/145 [==============================] - 1s 4ms/step - loss: 0.0749 -
accuracy: 0.9724 - val_loss: 0.1823 - val_accuracy: 0.9394
Epoch 670/1000
145/145 [==============================] - 1s 4ms/step - loss: 0.0744 -
accuracy: 0.9738 - val_loss: 0.1998 - val_accuracy: 0.9449
Epoch 671/1000
145/145 [==============================] - 1s 4ms/step - loss: 0.0766 -
accuracy: 0.9696 - val_loss: 0.2313 - val_accuracy: 0.9394
Epoch 672/1000
145/145 [==============================] - 1s 4ms/step - loss: 0.0759 -
accuracy: 0.9696 - val_loss: 0.2047 - val_accuracy: 0.9449
Epoch 673/1000
145/145 [==============================] - 1s 4ms/step - loss: 0.0731 -
accuracy: 0.9731 - val_loss: 0.1809 - val_accuracy: 0.9339
Epoch 674/1000
145/145 [==============================] - 1s 5ms/step - loss: 0.0761 -
accuracy: 0.9703 - val_loss: 0.1794 - val_accuracy: 0.9394
Epoch 675/1000
145/145 [==============================] - 1s 4ms/step - loss: 0.0722 -
accuracy: 0.9731 - val_loss: 0.1969 - val_accuracy: 0.9449
Epoch 676/1000
145/145 [==============================] - 1s 4ms/step - loss: 0.0736 -
accuracy: 0.9731 - val_loss: 0.2056 - val_accuracy: 0.9394
Epoch 677/1000
```

```
145/145 [==============================] - 1s 4ms/step - loss: 0.0713 -
accuracy: 0.9703 - val_loss: 0.1999 - val_accuracy: 0.9421
Epoch 678/1000
145/145 [==============================] - 1s 4ms/step - loss: 0.0784 -
accuracy: 0.9683 - val_loss: 0.2010 - val_accuracy: 0.9421
Epoch 679/1000
145/145 [==============================] - 1s 4ms/step - loss: 0.0750 -
accuracy: 0.9717 - val_loss: 0.1980 - val_accuracy: 0.9421
Epoch 680/1000
145/145 [==============================] - 1s 4ms/step - loss: 0.0750 -
accuracy: 0.9731 - val_loss: 0.2052 - val_accuracy: 0.9394
Epoch 681/1000
145/145 [==============================] - 1s 4ms/step - loss: 0.0759 -
accuracy: 0.9710 - val_loss: 0.1713 - val_accuracy: 0.9421
Epoch 682/1000
145/145 [==============================] - 1s 5ms/step - loss: 0.0754 -
accuracy: 0.9738 - val_loss: 0.1919 - val_accuracy: 0.9366
Epoch 683/1000
145/145 [==============================] - 1s 4ms/step - loss: 0.0732 -
accuracy: 0.9689 - val_loss: 0.1972 - val_accuracy: 0.9366
Epoch 684/1000
145/145 [==============================] - 1s 4ms/step - loss: 0.0759 -
accuracy: 0.9710 - val_loss: 0.2064 - val_accuracy: 0.9421
Epoch 685/1000
145/145 [==============================] - 1s 4ms/step - loss: 0.0751 -
accuracy: 0.9710 - val_loss: 0.2210 - val_accuracy: 0.9421
Epoch 686/1000
145/145 [==============================] - 1s 4ms/step - loss: 0.0741 -
accuracy: 0.9676 - val_loss: 0.1971 - val_accuracy: 0.9449
Epoch 687/1000
145/145 [==============================] - 1s 4ms/step - loss: 0.0762 -
accuracy: 0.9724 - val_loss: 0.1945 - val_accuracy: 0.9421
Epoch 688/1000
145/145 [==============================] - 1s 4ms/step - loss: 0.0754 -
accuracy: 0.9703 - val_loss: 0.2111 - val_accuracy: 0.9449
Epoch 689/1000
145/145 [==============================] - 1s 4ms/step - loss: 0.0748 -
accuracy: 0.9724 - val_loss: 0.1981 - val_accuracy: 0.9394
Epoch 690/1000
145/145 [==============================] - 1s 4ms/step - loss: 0.0743 -
accuracy: 0.9683 - val_loss: 0.2023 - val_accuracy: 0.9449
Epoch 691/1000
145/145 [==============================] - 1s 4ms/step - loss: 0.0735 -
accuracy: 0.9710 - val_loss: 0.2232 - val_accuracy: 0.9394
Epoch 692/1000
145/145 [==============================] - 1s 4ms/step - loss: 0.0749 -
accuracy: 0.9703 - val_loss: 0.2051 - val_accuracy: 0.9449
Epoch 693/1000
```

```
145/145 [==============================] - 1s 4ms/step - loss: 0.0744 -
accuracy: 0.9710 - val_loss: 0.2114 - val_accuracy: 0.9449
Epoch 694/1000
145/145 [==============================] - 1s 4ms/step - loss: 0.0747 -
accuracy: 0.9696 - val_loss: 0.1944 - val_accuracy: 0.9421
Epoch 695/1000
145/145 [==============================] - 1s 4ms/step - loss: 0.0763 -
accuracy: 0.9710 - val_loss: 0.1921 - val_accuracy: 0.9449
Epoch 696/1000
145/145 [==============================] - 1s 4ms/step - loss: 0.0725 -
accuracy: 0.9724 - val_loss: 0.1964 - val_accuracy: 0.9421
Epoch 697/1000
145/145 [==============================] - 1s 5ms/step - loss: 0.0722 -
accuracy: 0.9724 - val_loss: 0.2227 - val_accuracy: 0.9394
Epoch 698/1000
145/145 [==============================] - 1s 4ms/step - loss: 0.0762 -
accuracy: 0.9683 - val_loss: 0.2270 - val_accuracy: 0.9449
Epoch 699/1000
145/145 [==============================] - 1s 4ms/step - loss: 0.0763 -
accuracy: 0.9703 - val_loss: 0.2025 - val_accuracy: 0.9394
Epoch 700/1000
145/145 [==============================] - 1s 4ms/step - loss: 0.0746 -
accuracy: 0.9717 - val_loss: 0.2122 - val_accuracy: 0.9449
Epoch 701/1000
145/145 [==============================] - 1s 5ms/step - loss: 0.0746 -
accuracy: 0.9724 - val_loss: 0.1874 - val_accuracy: 0.9449
Epoch 702/1000
145/145 [==============================] - 1s 4ms/step - loss: 0.0749 -
accuracy: 0.9717 - val_loss: 0.1994 - val_accuracy: 0.9449
Epoch 703/1000
145/145 [==============================] - 1s 4ms/step - loss: 0.0738 -
accuracy: 0.9724 - val_loss: 0.2094 - val_accuracy: 0.9394
Epoch 704/1000
145/145 [==============================] - 1s 4ms/step - loss: 0.0768 -
accuracy: 0.9710 - val_loss: 0.1924 - val_accuracy: 0.9421
Epoch 705/1000
145/145 [==============================] - 1s 5ms/step - loss: 0.0756 -
accuracy: 0.9717 - val_loss: 0.1944 - val_accuracy: 0.9449
Epoch 706/1000
145/145 [==============================] - 1s 4ms/step - loss: 0.0739 -
accuracy: 0.9724 - val_loss: 0.1847 - val_accuracy: 0.9449
Epoch 707/1000
145/145 [==============================] - 1s 4ms/step - loss: 0.0771 -
accuracy: 0.9696 - val_loss: 0.2087 - val_accuracy: 0.9449
Epoch 708/1000
145/145 [==============================] - 1s 4ms/step - loss: 0.0743 -
accuracy: 0.9696 - val_loss: 0.1866 - val_accuracy: 0.9449
Epoch 709/1000
```

```
145/145 [==============================] - 1s 4ms/step - loss: 0.0738 -
accuracy: 0.9710 - val_loss: 0.1924 - val_accuracy: 0.9449
Epoch 710/1000
145/145 [==============================] - 1s 4ms/step - loss: 0.0743 -
accuracy: 0.9717 - val_loss: 0.2016 - val_accuracy: 0.9449
Epoch 711/1000
145/145 [==============================] - 1s 4ms/step - loss: 0.0748 -
accuracy: 0.9717 - val_loss: 0.2279 - val_accuracy: 0.9449
Epoch 712/1000
145/145 [==============================] - 1s 4ms/step - loss: 0.0789 -
accuracy: 0.9724 - val_loss: 0.2033 - val_accuracy: 0.9339
Epoch 713/1000
145/145 [==============================] - 1s 4ms/step - loss: 0.0750 -
accuracy: 0.9703 - val_loss: 0.2066 - val_accuracy: 0.9449
Epoch 714/1000
145/145 [==============================] - 1s 5ms/step - loss: 0.0783 -
accuracy: 0.9696 - val_loss: 0.1894 - val_accuracy: 0.9449
Epoch 715/1000
145/145 [==============================] - 1s 4ms/step - loss: 0.0726 -
accuracy: 0.9724 - val_loss: 0.2181 - val_accuracy: 0.9394
Epoch 716/1000
145/145 [==============================] - 1s 5ms/step - loss: 0.0744 -
accuracy: 0.9710 - val_loss: 0.2065 - val_accuracy: 0.9449
Epoch 717/1000
145/145 [==============================] - 1s 4ms/step - loss: 0.0737 -
accuracy: 0.9731 - val_loss: 0.2080 - val_accuracy: 0.9449
Epoch 718/1000
145/145 [==============================] - 1s 5ms/step - loss: 0.0734 -
accuracy: 0.9724 - val_loss: 0.2025 - val_accuracy: 0.9449
Epoch 719/1000
145/145 [==============================] - 1s 5ms/step - loss: 0.0746 -
accuracy: 0.9717 - val_loss: 0.1944 - val_accuracy: 0.9421
Epoch 720/1000
145/145 [==============================] - 1s 4ms/step - loss: 0.0752 -
accuracy: 0.9731 - val_loss: 0.1930 - val_accuracy: 0.9394
Epoch 721/1000
145/145 [==============================] - 1s 5ms/step - loss: 0.0766 -
accuracy: 0.9717 - val_loss: 0.1925 - val_accuracy: 0.9421
Epoch 722/1000
145/145 [==============================] - 1s 5ms/step - loss: 0.0737 -
accuracy: 0.9717 - val_loss: 0.1922 - val_accuracy: 0.9394
Epoch 723/1000
145/145 [==============================] - 1s 4ms/step - loss: 0.0726 -
accuracy: 0.9724 - val_loss: 0.2043 - val_accuracy: 0.9394
Epoch 724/1000
145/145 [==============================] - 1s 4ms/step - loss: 0.0737 -
accuracy: 0.9717 - val_loss: 0.2200 - val_accuracy: 0.9449
Epoch 725/1000
```

```
145/145 [==============================] - 1s 4ms/step - loss: 0.0746 -
accuracy: 0.9703 - val_loss: 0.2067 - val_accuracy: 0.9449
Epoch 726/1000
145/145 [==============================] - 1s 4ms/step - loss: 0.0710 -
accuracy: 0.9724 - val_loss: 0.2181 - val_accuracy: 0.9449
Epoch 727/1000
145/145 [==============================] - 1s 4ms/step - loss: 0.0764 -
accuracy: 0.9710 - val_loss: 0.1970 - val_accuracy: 0.9394
Epoch 728/1000
145/145 [==============================] - 1s 4ms/step - loss: 0.0746 -
accuracy: 0.9724 - val_loss: 0.1929 - val_accuracy: 0.9449
Epoch 729/1000
145/145 [==============================] - 1s 4ms/step - loss: 0.0737 -
accuracy: 0.9724 - val_loss: 0.1828 - val_accuracy: 0.9449
Epoch 730/1000
145/145 [==============================] - 1s 4ms/step - loss: 0.0743 -
accuracy: 0.9717 - val_loss: 0.2089 - val_accuracy: 0.9449
Epoch 731/1000
145/145 [==============================] - 1s 4ms/step - loss: 0.0732 -
accuracy: 0.9717 - val_loss: 0.2023 - val_accuracy: 0.9421
Epoch 732/1000
145/145 [==============================] - 1s 4ms/step - loss: 0.0741 -
accuracy: 0.9696 - val_loss: 0.1916 - val_accuracy: 0.9421
Epoch 733/1000
145/145 [==============================] - 1s 4ms/step - loss: 0.0734 -
accuracy: 0.9731 - val_loss: 0.2152 - val_accuracy: 0.9394
Epoch 734/1000
145/145 [==============================] - 1s 4ms/step - loss: 0.0732 -
accuracy: 0.9710 - val_loss: 0.2156 - val_accuracy: 0.9421
Epoch 735/1000
145/145 [==============================] - 1s 4ms/step - loss: 0.0752 -
accuracy: 0.9703 - val_loss: 0.2031 - val_accuracy: 0.9366
Epoch 736/1000
145/145 [==============================] - 1s 4ms/step - loss: 0.0781 -
accuracy: 0.9710 - val_loss: 0.2148 - val_accuracy: 0.9394
Epoch 737/1000
145/145 [==============================] - 1s 4ms/step - loss: 0.0733 -
accuracy: 0.9703 - val_loss: 0.2132 - val_accuracy: 0.9394
Epoch 738/1000
145/145 [==============================] - 1s 4ms/step - loss: 0.0737 -
accuracy: 0.9696 - val_loss: 0.2237 - val_accuracy: 0.9394
Epoch 739/1000
145/145 [==============================] - 1s 4ms/step - loss: 0.0741 -
accuracy: 0.9703 - val_loss: 0.2153 - val_accuracy: 0.9394
Epoch 740/1000
145/145 [==============================] - 1s 4ms/step - loss: 0.0752 -
accuracy: 0.9724 - val_loss: 0.2014 - val_accuracy: 0.9449
Epoch 741/1000
```

```
145/145 [==============================] - 1s 4ms/step - loss: 0.0735 -
accuracy: 0.9703 - val_loss: 0.1972 - val_accuracy: 0.9339
Epoch 742/1000
145/145 [==============================] - 1s 4ms/step - loss: 0.0746 -
accuracy: 0.9703 - val_loss: 0.2066 - val_accuracy: 0.9449
Epoch 743/1000
145/145 [==============================] - 1s 4ms/step - loss: 0.0747 -
accuracy: 0.9689 - val_loss: 0.2067 - val_accuracy: 0.9394
Epoch 744/1000
145/145 [==============================] - 1s 4ms/step - loss: 0.0761 -
accuracy: 0.9710 - val_loss: 0.2024 - val_accuracy: 0.9449
Epoch 745/1000
145/145 [==============================] - 1s 4ms/step - loss: 0.0746 -
accuracy: 0.9724 - val_loss: 0.1951 - val_accuracy: 0.9449
Epoch 746/1000
145/145 [==============================] - 1s 4ms/step - loss: 0.0760 -
accuracy: 0.9717 - val_loss: 0.1995 - val_accuracy: 0.9449
Epoch 747/1000
145/145 [==============================] - 1s 4ms/step - loss: 0.0729 -
accuracy: 0.9710 - val_loss: 0.2228 - val_accuracy: 0.9394
Epoch 748/1000
145/145 [==============================] - 1s 4ms/step - loss: 0.0755 -
accuracy: 0.9689 - val_loss: 0.1999 - val_accuracy: 0.9449
Epoch 749/1000
145/145 [==============================] - 1s 4ms/step - loss: 0.0755 -
accuracy: 0.9731 - val_loss: 0.2028 - val_accuracy: 0.9449
Epoch 750/1000
145/145 [==============================] - 1s 4ms/step - loss: 0.0758 -
accuracy: 0.9710 - val_loss: 0.1807 - val_accuracy: 0.9394
Epoch 751/1000
145/145 [==============================] - 1s 4ms/step - loss: 0.0765 -
accuracy: 0.9683 - val_loss: 0.2038 - val_accuracy: 0.9449
Epoch 752/1000
145/145 [==============================] - 1s 4ms/step - loss: 0.0733 -
accuracy: 0.9731 - val_loss: 0.2270 - val_accuracy: 0.9311
Epoch 753/1000
145/145 [==============================] - 1s 4ms/step - loss: 0.0764 -
accuracy: 0.9731 - val_loss: 0.2043 - val_accuracy: 0.9394
Epoch 754/1000
145/145 [==============================] - 1s 4ms/step - loss: 0.0733 -
accuracy: 0.9717 - val_loss: 0.2058 - val_accuracy: 0.9394
Epoch 755/1000
145/145 [==============================] - 1s 4ms/step - loss: 0.0740 -
accuracy: 0.9717 - val_loss: 0.1939 - val_accuracy: 0.9449
Epoch 756/1000
145/145 [==============================] - 1s 4ms/step - loss: 0.0731 -
accuracy: 0.9717 - val_loss: 0.2057 - val_accuracy: 0.9394
Epoch 757/1000
```

```
145/145 [==============================] - 1s 4ms/step - loss: 0.0737 -
accuracy: 0.9738 - val_loss: 0.1849 - val_accuracy: 0.9394
Epoch 758/1000
145/145 [==============================] - 1s 5ms/step - loss: 0.0739 -
accuracy: 0.9683 - val_loss: 0.1872 - val_accuracy: 0.9339
Epoch 759/1000
145/145 [==============================] - 1s 4ms/step - loss: 0.0750 -
accuracy: 0.9696 - val_loss: 0.1931 - val_accuracy: 0.9339
Epoch 760/1000
145/145 [==============================] - 1s 4ms/step - loss: 0.0799 -
accuracy: 0.9655 - val_loss: 0.2016 - val_accuracy: 0.9421
Epoch 761/1000
145/145 [==============================] - 1s 4ms/step - loss: 0.0722 -
accuracy: 0.9731 - val_loss: 0.2228 - val_accuracy: 0.9449
Epoch 762/1000
145/145 [==============================] - 1s 4ms/step - loss: 0.0739 -
accuracy: 0.9731 - val_loss: 0.2055 - val_accuracy: 0.9449
Epoch 763/1000
145/145 [==============================] - 1s 4ms/step - loss: 0.0754 -
accuracy: 0.9717 - val_loss: 0.2077 - val_accuracy: 0.9421
Epoch 764/1000
145/145 [==============================] - 1s 4ms/step - loss: 0.0754 -
accuracy: 0.9710 - val_loss: 0.2040 - val_accuracy: 0.9421
Epoch 765/1000
145/145 [==============================] - 1s 4ms/step - loss: 0.0745 -
accuracy: 0.9703 - val_loss: 0.2123 - val_accuracy: 0.9449
Epoch 766/1000
145/145 [==============================] - 1s 4ms/step - loss: 0.0723 -
accuracy: 0.9724 - val_loss: 0.1912 - val_accuracy: 0.9421
Epoch 767/1000
145/145 [==============================] - 1s 4ms/step - loss: 0.0713 -
accuracy: 0.9724 - val_loss: 0.1940 - val_accuracy: 0.9394
Epoch 768/1000
145/145 [==============================] - 1s 4ms/step - loss: 0.0738 -
accuracy: 0.9724 - val_loss: 0.2123 - val_accuracy: 0.9394
Epoch 769/1000
145/145 [==============================] - 1s 5ms/step - loss: 0.0733 -
accuracy: 0.9724 - val_loss: 0.2093 - val_accuracy: 0.9449
Epoch 770/1000
145/145 [==============================] - 1s 4ms/step - loss: 0.0740 -
accuracy: 0.9717 - val_loss: 0.1884 - val_accuracy: 0.9366
Epoch 771/1000
145/145 [==============================] - 1s 5ms/step - loss: 0.0723 -
accuracy: 0.9717 - val_loss: 0.1843 - val_accuracy: 0.9394
Epoch 772/1000
145/145 [==============================] - 1s 4ms/step - loss: 0.0744 -
accuracy: 0.9724 - val_loss: 0.2125 - val_accuracy: 0.9366
Epoch 773/1000
```

```
145/145 [==============================] - 1s 4ms/step - loss: 0.0764 -
accuracy: 0.9710 - val_loss: 0.2110 - val_accuracy: 0.9449
Epoch 774/1000
145/145 [==============================] - 1s 4ms/step - loss: 0.0723 -
accuracy: 0.9703 - val_loss: 0.1887 - val_accuracy: 0.9421
Epoch 775/1000
145/145 [==============================] - 1s 4ms/step - loss: 0.0750 -
accuracy: 0.9710 - val_loss: 0.1978 - val_accuracy: 0.9449
Epoch 776/1000
145/145 [==============================] - 1s 4ms/step - loss: 0.0766 -
accuracy: 0.9710 - val_loss: 0.2129 - val_accuracy: 0.9449
Epoch 777/1000
145/145 [==============================] - 1s 4ms/step - loss: 0.0731 -
accuracy: 0.9710 - val_loss: 0.2113 - val_accuracy: 0.9394
Epoch 778/1000
145/145 [==============================] - 1s 4ms/step - loss: 0.0751 -
accuracy: 0.9683 - val_loss: 0.1982 - val_accuracy: 0.9421
Epoch 779/1000
145/145 [==============================] - 1s 4ms/step - loss: 0.0738 -
accuracy: 0.9717 - val_loss: 0.1906 - val_accuracy: 0.9366
Epoch 780/1000
145/145 [==============================] - 1s 4ms/step - loss: 0.0733 -
accuracy: 0.9724 - val_loss: 0.2145 - val_accuracy: 0.9449
Epoch 781/1000
145/145 [==============================] - 1s 4ms/step - loss: 0.0726 -
accuracy: 0.9731 - val_loss: 0.1893 - val_accuracy: 0.9366
Epoch 782/1000
145/145 [==============================] - 1s 4ms/step - loss: 0.0742 -
accuracy: 0.9738 - val_loss: 0.2157 - val_accuracy: 0.9449
Epoch 783/1000
145/145 [==============================] - 1s 4ms/step - loss: 0.0736 -
accuracy: 0.9731 - val_loss: 0.2065 - val_accuracy: 0.9449
Epoch 784/1000
145/145 [==============================] - 1s 4ms/step - loss: 0.0745 -
accuracy: 0.9703 - val_loss: 0.1930 - val_accuracy: 0.9449
Epoch 785/1000
145/145 [==============================] - 1s 4ms/step - loss: 0.0756 -
accuracy: 0.9724 - val_loss: 0.1994 - val_accuracy: 0.9394
Epoch 786/1000
145/145 [==============================] - 1s 5ms/step - loss: 0.0735 -
accuracy: 0.9689 - val_loss: 0.1786 - val_accuracy: 0.9394
Epoch 787/1000
145/145 [==============================] - 1s 4ms/step - loss: 0.0707 -
accuracy: 0.9731 - val_loss: 0.1980 - val_accuracy: 0.9421
Epoch 788/1000
145/145 [==============================] - 1s 5ms/step - loss: 0.0722 -
accuracy: 0.9717 - val_loss: 0.1956 - val_accuracy: 0.9394
Epoch 789/1000
```

```
145/145 [==============================] - 1s 4ms/step - loss: 0.0733 -
accuracy: 0.9731 - val_loss: 0.2195 - val_accuracy: 0.9449
Epoch 790/1000
145/145 [==============================] - 1s 4ms/step - loss: 0.0756 -
accuracy: 0.9710 - val_loss: 0.1783 - val_accuracy: 0.9394
Epoch 791/1000
145/145 [==============================] - 1s 4ms/step - loss: 0.0733 -
accuracy: 0.9717 - val_loss: 0.2080 - val_accuracy: 0.9449
Epoch 792/1000
145/145 [==============================] - 1s 4ms/step - loss: 0.0743 -
accuracy: 0.9724 - val_loss: 0.2246 - val_accuracy: 0.9394
Epoch 793/1000
145/145 [==============================] - 1s 4ms/step - loss: 0.0745 -
accuracy: 0.9717 - val_loss: 0.1859 - val_accuracy: 0.9421
Epoch 794/1000
145/145 [==============================] - 1s 4ms/step - loss: 0.0745 -
accuracy: 0.9724 - val_loss: 0.1892 - val_accuracy: 0.9339
Epoch 795/1000
145/145 [==============================] - 1s 4ms/step - loss: 0.0747 -
accuracy: 0.9738 - val_loss: 0.2109 - val_accuracy: 0.9394
Epoch 796/1000
145/145 [==============================] - 1s 4ms/step - loss: 0.0763 -
accuracy: 0.9676 - val_loss: 0.1920 - val_accuracy: 0.9421
Epoch 797/1000
145/145 [==============================] - 1s 4ms/step - loss: 0.0745 -
accuracy: 0.9683 - val_loss: 0.1794 - val_accuracy: 0.9366
Epoch 798/1000
145/145 [==============================] - 1s 4ms/step - loss: 0.0754 -
accuracy: 0.9731 - val_loss: 0.2052 - val_accuracy: 0.9449
Epoch 799/1000
145/145 [==============================] - 1s 4ms/step - loss: 0.0730 -
accuracy: 0.9724 - val_loss: 0.1829 - val_accuracy: 0.9421
Epoch 800/1000
145/145 [==============================] - 1s 4ms/step - loss: 0.0734 -
accuracy: 0.9717 - val_loss: 0.2022 - val_accuracy: 0.9449
Epoch 801/1000
145/145 [==============================] - 1s 4ms/step - loss: 0.0745 -
accuracy: 0.9703 - val_loss: 0.1895 - val_accuracy: 0.9449
Epoch 802/1000
145/145 [==============================] - 1s 4ms/step - loss: 0.0738 -
accuracy: 0.9724 - val_loss: 0.1953 - val_accuracy: 0.9449
Epoch 803/1000
145/145 [==============================] - 1s 4ms/step - loss: 0.0737 -
accuracy: 0.9724 - val_loss: 0.1829 - val_accuracy: 0.9421
Epoch 804/1000
145/145 [==============================] - 1s 4ms/step - loss: 0.0740 -
accuracy: 0.9731 - val_loss: 0.2072 - val_accuracy: 0.9449
Epoch 805/1000
```

```
145/145 [==============================] - 1s 4ms/step - loss: 0.0755 -
accuracy: 0.9710 - val_loss: 0.2231 - val_accuracy: 0.9449
Epoch 806/1000
145/145 [==============================] - 1s 4ms/step - loss: 0.0752 -
accuracy: 0.9724 - val_loss: 0.1867 - val_accuracy: 0.9421
Epoch 807/1000
145/145 [==============================] - 1s 4ms/step - loss: 0.0734 -
accuracy: 0.9724 - val_loss: 0.1855 - val_accuracy: 0.9449
Epoch 808/1000
145/145 [==============================] - 1s 4ms/step - loss: 0.0746 -
accuracy: 0.9717 - val_loss: 0.1865 - val_accuracy: 0.9366
Epoch 809/1000
145/145 [==============================] - 1s 4ms/step - loss: 0.0722 -
accuracy: 0.9731 - val_loss: 0.2015 - val_accuracy: 0.9449
Epoch 810/1000
145/145 [==============================] - 1s 5ms/step - loss: 0.0747 -
accuracy: 0.9710 - val_loss: 0.1974 - val_accuracy: 0.9421
Epoch 811/1000
145/145 [==============================] - 1s 5ms/step - loss: 0.0732 -
accuracy: 0.9731 - val_loss: 0.2041 - val_accuracy: 0.9394
Epoch 812/1000
145/145 [==============================] - 1s 4ms/step - loss: 0.0717 -
accuracy: 0.9710 - val_loss: 0.1896 - val_accuracy: 0.9504
Epoch 813/1000
145/145 [==============================] - 1s 4ms/step - loss: 0.0756 -
accuracy: 0.9724 - val_loss: 0.1856 - val_accuracy: 0.9394
Epoch 814/1000
145/145 [==============================] - 1s 4ms/step - loss: 0.0749 -
accuracy: 0.9724 - val_loss: 0.2037 - val_accuracy: 0.9449
Epoch 815/1000
145/145 [==============================] - 1s 4ms/step - loss: 0.0727 -
accuracy: 0.9724 - val_loss: 0.2332 - val_accuracy: 0.9339
Epoch 816/1000
145/145 [==============================] - 1s 5ms/step - loss: 0.0735 -
accuracy: 0.9703 - val_loss: 0.1859 - val_accuracy: 0.9449
Epoch 817/1000
145/145 [==============================] - 1s 4ms/step - loss: 0.0728 -
accuracy: 0.9731 - val_loss: 0.2197 - val_accuracy: 0.9394
Epoch 818/1000
145/145 [==============================] - 1s 4ms/step - loss: 0.0733 -
accuracy: 0.9717 - val_loss: 0.2089 - val_accuracy: 0.9394
Epoch 819/1000
145/145 [==============================] - 1s 4ms/step - loss: 0.0762 -
accuracy: 0.9676 - val_loss: 0.1906 - val_accuracy: 0.9366
Epoch 820/1000
145/145 [==============================] - 1s 4ms/step - loss: 0.0741 -
accuracy: 0.9731 - val_loss: 0.1925 - val_accuracy: 0.9449
Epoch 821/1000
```

```
145/145 [==============================] - 1s 4ms/step - loss: 0.0749 -
accuracy: 0.9703 - val_loss: 0.1979 - val_accuracy: 0.9449
Epoch 822/1000
145/145 [==============================] - 1s 5ms/step - loss: 0.0735 -
accuracy: 0.9710 - val_loss: 0.1930 - val_accuracy: 0.9421
Epoch 823/1000
145/145 [==============================] - 1s 4ms/step - loss: 0.0755 -
accuracy: 0.9703 - val_loss: 0.1929 - val_accuracy: 0.9366
Epoch 824/1000
145/145 [==============================] - 1s 4ms/step - loss: 0.0732 -
accuracy: 0.9710 - val_loss: 0.1860 - val_accuracy: 0.9394
Epoch 825/1000
145/145 [==============================] - 1s 4ms/step - loss: 0.0734 -
accuracy: 0.9738 - val_loss: 0.1941 - val_accuracy: 0.9394
Epoch 826/1000
145/145 [==============================] - 1s 4ms/step - loss: 0.0716 -
accuracy: 0.9676 - val_loss: 0.1955 - val_accuracy: 0.9449
Epoch 827/1000
145/145 [==============================] - 1s 4ms/step - loss: 0.0755 -
accuracy: 0.9703 - val_loss: 0.1925 - val_accuracy: 0.9449
Epoch 828/1000
145/145 [==============================] - 1s 4ms/step - loss: 0.0746 -
accuracy: 0.9724 - val_loss: 0.1874 - val_accuracy: 0.9394
Epoch 829/1000
145/145 [==============================] - 1s 4ms/step - loss: 0.0875 -
accuracy: 0.9724 - val_loss: 0.2257 - val_accuracy: 0.9366
Epoch 830/1000
145/145 [==============================] - 1s 4ms/step - loss: 0.0729 -
accuracy: 0.9717 - val_loss: 0.2065 - val_accuracy: 0.9366
Epoch 831/1000
145/145 [==============================] - 1s 4ms/step - loss: 0.0727 -
accuracy: 0.9724 - val_loss: 0.1954 - val_accuracy: 0.9394
Epoch 832/1000
145/145 [==============================] - 1s 4ms/step - loss: 0.0743 -
accuracy: 0.9710 - val_loss: 0.1970 - val_accuracy: 0.9394
Epoch 833/1000
145/145 [==============================] - 1s 4ms/step - loss: 0.0750 -
accuracy: 0.9710 - val_loss: 0.1886 - val_accuracy: 0.9421
Epoch 834/1000
145/145 [==============================] - 1s 5ms/step - loss: 0.0706 -
accuracy: 0.9710 - val_loss: 0.2144 - val_accuracy: 0.9421
Epoch 835/1000
145/145 [==============================] - 1s 5ms/step - loss: 0.0748 -
accuracy: 0.9731 - val_loss: 0.2002 - val_accuracy: 0.9449
Epoch 836/1000
145/145 [==============================] - 1s 4ms/step - loss: 0.0730 -
accuracy: 0.9710 - val_loss: 0.2145 - val_accuracy: 0.9449
Epoch 837/1000
```

```
145/145 [==============================] - 1s 4ms/step - loss: 0.0728 -
accuracy: 0.9710 - val_loss: 0.1937 - val_accuracy: 0.9449
Epoch 838/1000
145/145 [==============================] - 1s 4ms/step - loss: 0.0714 -
accuracy: 0.9717 - val_loss: 0.2089 - val_accuracy: 0.9421
Epoch 839/1000
145/145 [==============================] - 1s 4ms/step - loss: 0.0724 -
accuracy: 0.9724 - val_loss: 0.2185 - val_accuracy: 0.9449
Epoch 840/1000
145/145 [==============================] - 1s 4ms/step - loss: 0.0732 -
accuracy: 0.9724 - val_loss: 0.2040 - val_accuracy: 0.9421
Epoch 841/1000
145/145 [==============================] - 1s 4ms/step - loss: 0.0745 -
accuracy: 0.9731 - val_loss: 0.1904 - val_accuracy: 0.9421
Epoch 842/1000
145/145 [==============================] - 1s 4ms/step - loss: 0.0722 -
accuracy: 0.9717 - val_loss: 0.1972 - val_accuracy: 0.9421
Epoch 843/1000
145/145 [==============================] - 1s 4ms/step - loss: 0.0760 -
accuracy: 0.9689 - val_loss: 0.1909 - val_accuracy: 0.9366
Epoch 844/1000
145/145 [==============================] - 1s 4ms/step - loss: 0.0715 -
accuracy: 0.9717 - val_loss: 0.1961 - val_accuracy: 0.9421
Epoch 845/1000
145/145 [==============================] - 1s 5ms/step - loss: 0.0748 -
accuracy: 0.9724 - val_loss: 0.2050 - val_accuracy: 0.9449
Epoch 846/1000
145/145 [==============================] - 1s 5ms/step - loss: 0.0730 -
accuracy: 0.9731 - val_loss: 0.1863 - val_accuracy: 0.9366
Epoch 847/1000
145/145 [==============================] - 1s 4ms/step - loss: 0.0743 -
accuracy: 0.9731 - val_loss: 0.1926 - val_accuracy: 0.9366
Epoch 848/1000
145/145 [==============================] - 1s 5ms/step - loss: 0.0741 -
accuracy: 0.9717 - val_loss: 0.1863 - val_accuracy: 0.9421
Epoch 849/1000
145/145 [==============================] - 1s 5ms/step - loss: 0.0739 -
accuracy: 0.9724 - val_loss: 0.2099 - val_accuracy: 0.9394
Epoch 850/1000
145/145 [==============================] - 1s 4ms/step - loss: 0.0753 -
accuracy: 0.9724 - val_loss: 0.2083 - val_accuracy: 0.9366
Epoch 851/1000
145/145 [==============================] - 1s 4ms/step - loss: 0.0758 -
accuracy: 0.9703 - val_loss: 0.1854 - val_accuracy: 0.9394
Epoch 852/1000
145/145 [==============================] - 1s 4ms/step - loss: 0.0718 -
accuracy: 0.9703 - val_loss: 0.1981 - val_accuracy: 0.9421
Epoch 853/1000
```

```
145/145 [==============================] - 1s 4ms/step - loss: 0.0748 -
accuracy: 0.9710 - val_loss: 0.1949 - val_accuracy: 0.9449
Epoch 854/1000
145/145 [==============================] - 1s 4ms/step - loss: 0.0723 -
accuracy: 0.9724 - val_loss: 0.1880 - val_accuracy: 0.9421
Epoch 855/1000
145/145 [==============================] - 1s 5ms/step - loss: 0.0706 -
accuracy: 0.9731 - val_loss: 0.2300 - val_accuracy: 0.9394
Epoch 856/1000
145/145 [==============================] - 1s 5ms/step - loss: 0.0747 -
accuracy: 0.9696 - val_loss: 0.2132 - val_accuracy: 0.9449
Epoch 857/1000
145/145 [==============================] - 1s 4ms/step - loss: 0.0729 -
accuracy: 0.9717 - val_loss: 0.1983 - val_accuracy: 0.9449
Epoch 858/1000
145/145 [==============================] - 1s 4ms/step - loss: 0.0724 -
accuracy: 0.9731 - val_loss: 0.1864 - val_accuracy: 0.9421
Epoch 859/1000
145/145 [==============================] - 1s 5ms/step - loss: 0.0735 -
accuracy: 0.9717 - val_loss: 0.1993 - val_accuracy: 0.9421
Epoch 860/1000
145/145 [==============================] - 1s 4ms/step - loss: 0.0728 -
accuracy: 0.9731 - val_loss: 0.2039 - val_accuracy: 0.9449
Epoch 861/1000
145/145 [==============================] - 1s 4ms/step - loss: 0.0736 -
accuracy: 0.9689 - val_loss: 0.1857 - val_accuracy: 0.9421
Epoch 862/1000
145/145 [==============================] - 1s 4ms/step - loss: 0.0739 -
accuracy: 0.9738 - val_loss: 0.1909 - val_accuracy: 0.9394
Epoch 863/1000
145/145 [==============================] - 1s 4ms/step - loss: 0.0743 -
accuracy: 0.9703 - val_loss: 0.1943 - val_accuracy: 0.9449
Epoch 864/1000
145/145 [==============================] - 1s 4ms/step - loss: 0.0753 -
accuracy: 0.9717 - val_loss: 0.2133 - val_accuracy: 0.9421
Epoch 865/1000
145/145 [==============================] - 1s 4ms/step - loss: 0.0759 -
accuracy: 0.9696 - val_loss: 0.2141 - val_accuracy: 0.9449
Epoch 866/1000
145/145 [==============================] - 1s 4ms/step - loss: 0.0753 -
accuracy: 0.9703 - val_loss: 0.2034 - val_accuracy: 0.9421
Epoch 867/1000
145/145 [==============================] - 1s 4ms/step - loss: 0.0714 -
accuracy: 0.9724 - val_loss: 0.1974 - val_accuracy: 0.9449
Epoch 868/1000
145/145 [==============================] - 1s 4ms/step - loss: 0.0734 -
accuracy: 0.9724 - val_loss: 0.2205 - val_accuracy: 0.9394
Epoch 869/1000
```

```
145/145 [==============================] - 1s 5ms/step - loss: 0.0741 -
accuracy: 0.9703 - val_loss: 0.2141 - val_accuracy: 0.9449
Epoch 870/1000
145/145 [==============================] - 1s 5ms/step - loss: 0.0734 -
accuracy: 0.9731 - val_loss: 0.1899 - val_accuracy: 0.9421
Epoch 871/1000
145/145 [==============================] - 1s 5ms/step - loss: 0.0734 -
accuracy: 0.9724 - val_loss: 0.2175 - val_accuracy: 0.9394
Epoch 872/1000
145/145 [==============================] - 1s 5ms/step - loss: 0.0732 -
accuracy: 0.9738 - val_loss: 0.1856 - val_accuracy: 0.9449
Epoch 873/1000
145/145 [==============================] - 1s 5ms/step - loss: 0.0715 -
accuracy: 0.9710 - val_loss: 0.2063 - val_accuracy: 0.9339
Epoch 874/1000
145/145 [==============================] - 1s 4ms/step - loss: 0.0740 -
accuracy: 0.9724 - val_loss: 0.2095 - val_accuracy: 0.9449
Epoch 875/1000
145/145 [==============================] - 1s 4ms/step - loss: 0.0743 -
accuracy: 0.9710 - val_loss: 0.1961 - val_accuracy: 0.9339
Epoch 876/1000
145/145 [==============================] - 1s 5ms/step - loss: 0.0739 -
accuracy: 0.9703 - val_loss: 0.2110 - val_accuracy: 0.9421
Epoch 877/1000
145/145 [==============================] - 1s 4ms/step - loss: 0.0744 -
accuracy: 0.9717 - val_loss: 0.2144 - val_accuracy: 0.9421
Epoch 878/1000
145/145 [==============================] - 1s 4ms/step - loss: 0.0741 -
accuracy: 0.9724 - val_loss: 0.2036 - val_accuracy: 0.9449
Epoch 879/1000
145/145 [==============================] - 1s 4ms/step - loss: 0.0756 -
accuracy: 0.9724 - val_loss: 0.1884 - val_accuracy: 0.9366
Epoch 880/1000
145/145 [==============================] - 1s 4ms/step - loss: 0.0753 -
accuracy: 0.9710 - val_loss: 0.1993 - val_accuracy: 0.9421
Epoch 881/1000
145/145 [==============================] - 1s 4ms/step - loss: 0.0730 -
accuracy: 0.9738 - val_loss: 0.1866 - val_accuracy: 0.9421
Epoch 882/1000
145/145 [==============================] - 1s 4ms/step - loss: 0.0729 -
accuracy: 0.9724 - val_loss: 0.1855 - val_accuracy: 0.9449
Epoch 883/1000
145/145 [==============================] - 1s 4ms/step - loss: 0.0722 -
accuracy: 0.9717 - val_loss: 0.2174 - val_accuracy: 0.9394
Epoch 884/1000
145/145 [==============================] - 1s 4ms/step - loss: 0.0730 -
accuracy: 0.9717 - val_loss: 0.1902 - val_accuracy: 0.9449
Epoch 885/1000
```

```
145/145 [==============================] - 1s 5ms/step - loss: 0.0723 -
accuracy: 0.9738 - val_loss: 0.1891 - val_accuracy: 0.9366
Epoch 886/1000
145/145 [==============================] - 1s 4ms/step - loss: 0.0743 -
accuracy: 0.9710 - val_loss: 0.2073 - val_accuracy: 0.9449
Epoch 887/1000
145/145 [==============================] - 1s 5ms/step - loss: 0.0731 -
accuracy: 0.9731 - val_loss: 0.2075 - val_accuracy: 0.9394
Epoch 888/1000
145/145 [==============================] - 1s 5ms/step - loss: 0.0721 -
accuracy: 0.9703 - val_loss: 0.2242 - val_accuracy: 0.9394
Epoch 889/1000
145/145 [==============================] - 1s 4ms/step - loss: 0.0711 -
accuracy: 0.9717 - val_loss: 0.1759 - val_accuracy: 0.9421
Epoch 890/1000
145/145 [==============================] - 1s 4ms/step - loss: 0.0757 -
accuracy: 0.9710 - val_loss: 0.1983 - val_accuracy: 0.9421
Epoch 891/1000
145/145 [==============================] - 1s 5ms/step - loss: 0.0725 -
accuracy: 0.9738 - val_loss: 0.1941 - val_accuracy: 0.9449
Epoch 892/1000
145/145 [==============================] - 1s 4ms/step - loss: 0.0759 -
accuracy: 0.9731 - val_loss: 0.2019 - val_accuracy: 0.9449
Epoch 893/1000
145/145 [==============================] - 1s 4ms/step - loss: 0.0733 -
accuracy: 0.9717 - val_loss: 0.1947 - val_accuracy: 0.9394
Epoch 894/1000
145/145 [==============================] - 1s 4ms/step - loss: 0.0728 -
accuracy: 0.9724 - val_loss: 0.2194 - val_accuracy: 0.9449
Epoch 895/1000
145/145 [==============================] - 1s 4ms/step - loss: 0.0756 -
accuracy: 0.9696 - val_loss: 0.2018 - val_accuracy: 0.9421
Epoch 896/1000
145/145 [==============================] - 1s 5ms/step - loss: 0.0738 -
accuracy: 0.9696 - val_loss: 0.2012 - val_accuracy: 0.9394
Epoch 897/1000
145/145 [==============================] - 1s 5ms/step - loss: 0.0732 -
accuracy: 0.9731 - val_loss: 0.1989 - val_accuracy: 0.9394
Epoch 898/1000
145/145 [==============================] - 1s 4ms/step - loss: 0.0727 -
accuracy: 0.9724 - val_loss: 0.2241 - val_accuracy: 0.9449
Epoch 899/1000
145/145 [==============================] - 1s 4ms/step - loss: 0.0730 -
accuracy: 0.9731 - val_loss: 0.2004 - val_accuracy: 0.9421
Epoch 900/1000
145/145 [==============================] - 1s 5ms/step - loss: 0.0744 -
accuracy: 0.9689 - val_loss: 0.2083 - val_accuracy: 0.9449
Epoch 901/1000
```

```
145/145 [==============================] - 1s 4ms/step - loss: 0.0745 -
accuracy: 0.9717 - val_loss: 0.2093 - val_accuracy: 0.9449
Epoch 902/1000
145/145 [==============================] - 1s 4ms/step - loss: 0.0757 -
accuracy: 0.9710 - val_loss: 0.2285 - val_accuracy: 0.9449
Epoch 903/1000
145/145 [==============================] - 1s 4ms/step - loss: 0.0753 -
accuracy: 0.9717 - val_loss: 0.2169 - val_accuracy: 0.9449
Epoch 904/1000
145/145 [==============================] - 1s 4ms/step - loss: 0.0730 -
accuracy: 0.9724 - val_loss: 0.2053 - val_accuracy: 0.9449
Epoch 905/1000
145/145 [==============================] - 1s 5ms/step - loss: 0.0726 -
accuracy: 0.9717 - val_loss: 0.2346 - val_accuracy: 0.9449
Epoch 906/1000
145/145 [==============================] - 1s 4ms/step - loss: 0.0735 -
accuracy: 0.9717 - val_loss: 0.2118 - val_accuracy: 0.9449
Epoch 907/1000
145/145 [==============================] - 1s 5ms/step - loss: 0.0725 -
accuracy: 0.9717 - val_loss: 0.2151 - val_accuracy: 0.9394
Epoch 908/1000
145/145 [==============================] - 1s 4ms/step - loss: 0.0720 -
accuracy: 0.9717 - val_loss: 0.2067 - val_accuracy: 0.9449
Epoch 909/1000
145/145 [==============================] - 1s 4ms/step - loss: 0.0742 -
accuracy: 0.9717 - val_loss: 0.1905 - val_accuracy: 0.9394
Epoch 910/1000
145/145 [==============================] - 1s 5ms/step - loss: 0.0705 -
accuracy: 0.9717 - val_loss: 0.2298 - val_accuracy: 0.9394
Epoch 911/1000
145/145 [==============================] - 1s 4ms/step - loss: 0.0731 -
accuracy: 0.9696 - val_loss: 0.2160 - val_accuracy: 0.9394
Epoch 912/1000
145/145 [==============================] - 1s 4ms/step - loss: 0.0756 -
accuracy: 0.9703 - val_loss: 0.1994 - val_accuracy: 0.9449
Epoch 913/1000
145/145 [==============================] - 1s 5ms/step - loss: 0.0742 -
accuracy: 0.9717 - val_loss: 0.2050 - val_accuracy: 0.9366
Epoch 914/1000
145/145 [==============================] - 1s 5ms/step - loss: 0.0745 -
accuracy: 0.9724 - val_loss: 0.2474 - val_accuracy: 0.9449
Epoch 915/1000
145/145 [==============================] - 1s 4ms/step - loss: 0.0723 -
accuracy: 0.9724 - val_loss: 0.2224 - val_accuracy: 0.9421
Epoch 916/1000
145/145 [==============================] - 1s 4ms/step - loss: 0.0739 -
accuracy: 0.9717 - val_loss: 0.2126 - val_accuracy: 0.9449
Epoch 917/1000
```

```
145/145 [==============================] - 1s 4ms/step - loss: 0.0750 -
accuracy: 0.9724 - val_loss: 0.2083 - val_accuracy: 0.9421
Epoch 918/1000
145/145 [==============================] - 1s 5ms/step - loss: 0.0707 -
accuracy: 0.9710 - val_loss: 0.2319 - val_accuracy: 0.9421
Epoch 919/1000
145/145 [==============================] - 1s 5ms/step - loss: 0.0739 -
accuracy: 0.9724 - val_loss: 0.2234 - val_accuracy: 0.9449
Epoch 920/1000
145/145 [==============================] - 1s 4ms/step - loss: 0.0710 -
accuracy: 0.9703 - val_loss: 0.2031 - val_accuracy: 0.9449
Epoch 921/1000
145/145 [==============================] - 1s 5ms/step - loss: 0.0716 -
accuracy: 0.9696 - val_loss: 0.2171 - val_accuracy: 0.9449
Epoch 922/1000
145/145 [==============================] - 1s 4ms/step - loss: 0.0711 -
accuracy: 0.9724 - val_loss: 0.1964 - val_accuracy: 0.9421
Epoch 923/1000
145/145 [==============================] - 1s 4ms/step - loss: 0.0733 -
accuracy: 0.9703 - val_loss: 0.2278 - val_accuracy: 0.9449
Epoch 924/1000
145/145 [==============================] - 1s 4ms/step - loss: 0.0724 -
accuracy: 0.9731 - val_loss: 0.1964 - val_accuracy: 0.9394
Epoch 925/1000
145/145 [==============================] - 1s 4ms/step - loss: 0.0751 -
accuracy: 0.9676 - val_loss: 0.2231 - val_accuracy: 0.9394
Epoch 926/1000
145/145 [==============================] - 1s 5ms/step - loss: 0.0706 -
accuracy: 0.9752 - val_loss: 0.2137 - val_accuracy: 0.9394
Epoch 927/1000
145/145 [==============================] - 1s 5ms/step - loss: 0.0743 -
accuracy: 0.9724 - val_loss: 0.2192 - val_accuracy: 0.9449
Epoch 928/1000
145/145 [==============================] - 1s 5ms/step - loss: 0.0710 -
accuracy: 0.9724 - val_loss: 0.2063 - val_accuracy: 0.9449
Epoch 929/1000
145/145 [==============================] - 1s 4ms/step - loss: 0.0730 -
accuracy: 0.9703 - val_loss: 0.2091 - val_accuracy: 0.9421
Epoch 930/1000
145/145 [==============================] - 1s 5ms/step - loss: 0.0724 -
accuracy: 0.9731 - val_loss: 0.1948 - val_accuracy: 0.9421
Epoch 931/1000
145/145 [==============================] - 1s 4ms/step - loss: 0.0725 -
accuracy: 0.9710 - val_loss: 0.2085 - val_accuracy: 0.9421
Epoch 932/1000
145/145 [==============================] - 1s 4ms/step - loss: 0.0716 -
accuracy: 0.9724 - val_loss: 0.2111 - val_accuracy: 0.9366
Epoch 933/1000
```

```
145/145 [==============================] - 1s 4ms/step - loss: 0.0724 -
accuracy: 0.9731 - val_loss: 0.2191 - val_accuracy: 0.9449
Epoch 934/1000
145/145 [==============================] - 1s 4ms/step - loss: 0.0712 -
accuracy: 0.9731 - val_loss: 0.1991 - val_accuracy: 0.9394
Epoch 935/1000
145/145 [==============================] - 1s 5ms/step - loss: 0.0712 -
accuracy: 0.9738 - val_loss: 0.2053 - val_accuracy: 0.9366
Epoch 936/1000
145/145 [==============================] - 1s 4ms/step - loss: 0.0754 -
accuracy: 0.9710 - val_loss: 0.2013 - val_accuracy: 0.9421
Epoch 937/1000
145/145 [==============================] - 1s 4ms/step - loss: 0.0734 -
accuracy: 0.9717 - val_loss: 0.2122 - val_accuracy: 0.9394
Epoch 938/1000
145/145 [==============================] - 1s 4ms/step - loss: 0.0722 -
accuracy: 0.9731 - val_loss: 0.2146 - val_accuracy: 0.9449
Epoch 939/1000
145/145 [==============================] - 1s 4ms/step - loss: 0.0733 -
accuracy: 0.9724 - val_loss: 0.2234 - val_accuracy: 0.9449
Epoch 940/1000
145/145 [==============================] - 1s 4ms/step - loss: 0.0714 -
accuracy: 0.9717 - val_loss: 0.2024 - val_accuracy: 0.9366
Epoch 941/1000
145/145 [==============================] - 1s 4ms/step - loss: 0.0707 -
accuracy: 0.9717 - val_loss: 0.1909 - val_accuracy: 0.9421
Epoch 942/1000
145/145 [==============================] - 1s 4ms/step - loss: 0.0727 -
accuracy: 0.9724 - val_loss: 0.2113 - val_accuracy: 0.9421
Epoch 943/1000
145/145 [==============================] - 1s 5ms/step - loss: 0.0727 -
accuracy: 0.9724 - val_loss: 0.2077 - val_accuracy: 0.9421
Epoch 944/1000
145/145 [==============================] - 1s 4ms/step - loss: 0.0715 -
accuracy: 0.9703 - val_loss: 0.2024 - val_accuracy: 0.9421
Epoch 945/1000
145/145 [==============================] - 1s 4ms/step - loss: 0.0731 -
accuracy: 0.9717 - val_loss: 0.2127 - val_accuracy: 0.9394
Epoch 946/1000
145/145 [==============================] - 1s 5ms/step - loss: 0.0741 -
accuracy: 0.9696 - val_loss: 0.2073 - val_accuracy: 0.9421
Epoch 947/1000
145/145 [==============================] - 1s 4ms/step - loss: 0.0739 -
accuracy: 0.9717 - val_loss: 0.1971 - val_accuracy: 0.9449
Epoch 948/1000
145/145 [==============================] - 1s 4ms/step - loss: 0.0722 -
accuracy: 0.9738 - val_loss: 0.2029 - val_accuracy: 0.9421
Epoch 949/1000
```

```
145/145 [==============================] - 1s 4ms/step - loss: 0.0719 -
accuracy: 0.9738 - val_loss: 0.2014 - val_accuracy: 0.9394
Epoch 950/1000
145/145 [==============================] - 1s 5ms/step - loss: 0.0745 -
accuracy: 0.9717 - val_loss: 0.1884 - val_accuracy: 0.9449
Epoch 951/1000
145/145 [==============================] - 1s 4ms/step - loss: 0.0733 -
accuracy: 0.9703 - val_loss: 0.2015 - val_accuracy: 0.9421
Epoch 952/1000
145/145 [==============================] - 1s 4ms/step - loss: 0.0706 -
accuracy: 0.9724 - val_loss: 0.1735 - val_accuracy: 0.9311
Epoch 953/1000
145/145 [==============================] - 1s 4ms/step - loss: 0.0764 -
accuracy: 0.9696 - val_loss: 0.1773 - val_accuracy: 0.9421
Epoch 954/1000
145/145 [==============================] - 1s 5ms/step - loss: 0.0726 -
accuracy: 0.9724 - val_loss: 0.2048 - val_accuracy: 0.9421
Epoch 955/1000
145/145 [==============================] - 1s 5ms/step - loss: 0.0710 -
accuracy: 0.9738 - val_loss: 0.1977 - val_accuracy: 0.9421
Epoch 956/1000
145/145 [==============================] - 1s 5ms/step - loss: 0.0736 -
accuracy: 0.9731 - val_loss: 0.2302 - val_accuracy: 0.9394
Epoch 957/1000
145/145 [==============================] - 1s 4ms/step - loss: 0.0730 -
accuracy: 0.9717 - val_loss: 0.1904 - val_accuracy: 0.9284
Epoch 958/1000
145/145 [==============================] - 1s 4ms/step - loss: 0.0752 -
accuracy: 0.9724 - val_loss: 0.2193 - val_accuracy: 0.9449
Epoch 959/1000
145/145 [==============================] - 1s 4ms/step - loss: 0.0736 -
accuracy: 0.9710 - val_loss: 0.1874 - val_accuracy: 0.9421
Epoch 960/1000
145/145 [==============================] - 1s 4ms/step - loss: 0.0715 -
accuracy: 0.9710 - val_loss: 0.1900 - val_accuracy: 0.9421
Epoch 961/1000
145/145 [==============================] - 1s 5ms/step - loss: 0.0714 -
accuracy: 0.9731 - val_loss: 0.2183 - val_accuracy: 0.9449
Epoch 962/1000
145/145 [==============================] - 1s 4ms/step - loss: 0.0714 -
accuracy: 0.9724 - val_loss: 0.2100 - val_accuracy: 0.9421
Epoch 963/1000
145/145 [==============================] - 1s 5ms/step - loss: 0.0707 -
accuracy: 0.9731 - val_loss: 0.2159 - val_accuracy: 0.9394
Epoch 964/1000
145/145 [==============================] - 1s 4ms/step - loss: 0.0729 -
accuracy: 0.9717 - val_loss: 0.2010 - val_accuracy: 0.9421
Epoch 965/1000
```

```
145/145 [==============================] - 1s 4ms/step - loss: 0.0721 -
accuracy: 0.9738 - val_loss: 0.1772 - val_accuracy: 0.9421
Epoch 966/1000
145/145 [==============================] - 1s 5ms/step - loss: 0.0705 -
accuracy: 0.9731 - val_loss: 0.1844 - val_accuracy: 0.9339
Epoch 967/1000
145/145 [==============================] - 1s 4ms/step - loss: 0.0733 -
accuracy: 0.9717 - val_loss: 0.2067 - val_accuracy: 0.9366
Epoch 968/1000
145/145 [==============================] - 1s 5ms/step - loss: 0.0734 -
accuracy: 0.9710 - val_loss: 0.1916 - val_accuracy: 0.9394
Epoch 969/1000
145/145 [==============================] - 1s 5ms/step - loss: 0.0729 -
accuracy: 0.9717 - val_loss: 0.2024 - val_accuracy: 0.9449
Epoch 970/1000
145/145 [==============================] - 1s 4ms/step - loss: 0.0728 -
accuracy: 0.9738 - val_loss: 0.2272 - val_accuracy: 0.9449
Epoch 971/1000
145/145 [==============================] - 1s 5ms/step - loss: 0.0740 -
accuracy: 0.9703 - val_loss: 0.2297 - val_accuracy: 0.9449
Epoch 972/1000
145/145 [==============================] - 1s 5ms/step - loss: 0.0714 -
accuracy: 0.9717 - val_loss: 0.1915 - val_accuracy: 0.9449
Epoch 973/1000
145/145 [==============================] - 1s 5ms/step - loss: 0.0726 -
accuracy: 0.9738 - val_loss: 0.1860 - val_accuracy: 0.9366
Epoch 974/1000
145/145 [==============================] - 1s 4ms/step - loss: 0.0735 -
accuracy: 0.9703 - val_loss: 0.1846 - val_accuracy: 0.9421
Epoch 975/1000
145/145 [==============================] - 1s 5ms/step - loss: 0.0704 -
accuracy: 0.9724 - val_loss: 0.2020 - val_accuracy: 0.9449
Epoch 976/1000
145/145 [==============================] - 1s 4ms/step - loss: 0.0725 -
accuracy: 0.9717 - val_loss: 0.1731 - val_accuracy: 0.9394
Epoch 977/1000
145/145 [==============================] - 1s 5ms/step - loss: 0.0709 -
accuracy: 0.9717 - val_loss: 0.1810 - val_accuracy: 0.9421
Epoch 978/1000
145/145 [==============================] - 1s 4ms/step - loss: 0.0731 -
accuracy: 0.9717 - val_loss: 0.1868 - val_accuracy: 0.9421
Epoch 979/1000
145/145 [==============================] - 1s 4ms/step - loss: 0.0732 -
accuracy: 0.9731 - val_loss: 0.2065 - val_accuracy: 0.9421
Epoch 980/1000
145/145 [==============================] - 1s 4ms/step - loss: 0.0734 -
accuracy: 0.9696 - val_loss: 0.2120 - val_accuracy: 0.9421
Epoch 981/1000
```

```
145/145 [==============================] - 1s 4ms/step - loss: 0.0715 -
accuracy: 0.9724 - val_loss: 0.2025 - val_accuracy: 0.9449
Epoch 982/1000
145/145 [==============================] - 1s 4ms/step - loss: 0.0706 -
accuracy: 0.9738 - val_loss: 0.1899 - val_accuracy: 0.9394
Epoch 983/1000
145/145 [==============================] - 1s 5ms/step - loss: 0.0730 -
accuracy: 0.9738 - val_loss: 0.2010 - val_accuracy: 0.9421
Epoch 984/1000
145/145 [==============================] - 1s 4ms/step - loss: 0.0711 -
accuracy: 0.9724 - val_loss: 0.1970 - val_accuracy: 0.9449
Epoch 985/1000
145/145 [==============================] - 1s 5ms/step - loss: 0.0719 -
accuracy: 0.9724 - val_loss: 0.2097 - val_accuracy: 0.9449
Epoch 986/1000
145/145 [==============================] - 1s 4ms/step - loss: 0.0732 -
accuracy: 0.9717 - val_loss: 0.1956 - val_accuracy: 0.9421
Epoch 987/1000
145/145 [==============================] - 1s 4ms/step - loss: 0.0732 -
accuracy: 0.9717 - val_loss: 0.2148 - val_accuracy: 0.9421
Epoch 988/1000
145/145 [==============================] - 1s 4ms/step - loss: 0.0715 -
accuracy: 0.9745 - val_loss: 0.2043 - val_accuracy: 0.9449
Epoch 989/1000
145/145 [==============================] - 1s 5ms/step - loss: 0.0722 -
accuracy: 0.9731 - val_loss: 0.2061 - val_accuracy: 0.9394
Epoch 990/1000
145/145 [==============================] - 1s 5ms/step - loss: 0.0722 -
accuracy: 0.9683 - val_loss: 0.2000 - val_accuracy: 0.9421
Epoch 991/1000
145/145 [==============================] - 1s 4ms/step - loss: 0.0726 -
accuracy: 0.9731 - val_loss: 0.2302 - val_accuracy: 0.9449
Epoch 992/1000
145/145 [==============================] - 1s 4ms/step - loss: 0.0721 -
accuracy: 0.9724 - val_loss: 0.1992 - val_accuracy: 0.9421
Epoch 993/1000
145/145 [==============================] - 1s 4ms/step - loss: 0.0719 -
accuracy: 0.9710 - val_loss: 0.1982 - val_accuracy: 0.9421
Epoch 994/1000
145/145 [==============================] - 1s 4ms/step - loss: 0.0719 -
accuracy: 0.9724 - val_loss: 0.1931 - val_accuracy: 0.9449
Epoch 995/1000
145/145 [==============================] - 1s 4ms/step - loss: 0.0711 -
accuracy: 0.9710 - val_loss: 0.2050 - val_accuracy: 0.9449
Epoch 996/1000
145/145 [==============================] - 1s 5ms/step - loss: 0.0727 -
accuracy: 0.9717 - val_loss: 0.2010 - val_accuracy: 0.9449
Epoch 997/1000
```

```
145/145 [==============================] - 1s 5ms/step - loss: 0.0749 -
accuracy: 0.9710 - val_loss: 0.2102 - val_accuracy: 0.9449
Epoch 998/1000
145/145 [==============================] - 1s 5ms/step - loss: 0.0722 -
accuracy: 0.9703 - val_loss: 0.2035 - val_accuracy: 0.9421
Epoch 999/1000
145/145 [==============================] - 1s 4ms/step - loss: 0.0708 -
accuracy: 0.9724 - val_loss: 0.1972 - val_accuracy: 0.9449
Epoch 1000/1000
145/145 [==============================] - 1s 4ms/step - loss: 0.0726 -
accuracy: 0.9710 - val_loss: 0.1786 - val_accuracy: 0.9339
```

[45]:

```python
from matplotlib import pyplot as plt
history_dict = history.history

# learning curve
# accuracy
acc = history_dict['accuracy']
val_acc = history_dict['val_accuracy']

# loss
loss = history_dict['loss']
val_loss = history_dict['val_loss']

# range of X (no. of epochs)
epochs = range(1, len(acc) + 1)


#plt.set_figheight(6)
#plt.set_figwidth(9)
plt.grid(True)

# plot
# "r" is for "solid red line"
plt.plot(epochs, acc, 'r', label='Training accuracy')
# b is for "solid blue line"
plt.plot(epochs, val_acc, 'b', label='Validation accuracy')
plt.title('Training and validation accuracy')
plt.xlabel('Epochs')
plt.ylabel('Accuracy')

#plt.legend()

plt.legend(loc="lower right",prop=dict(size=11))
#plt.savefig('ROC_4.pdf', dpi=600)

plt.savefig('accuracy.pdf', dpi=600)
```

```
plt.show()
```

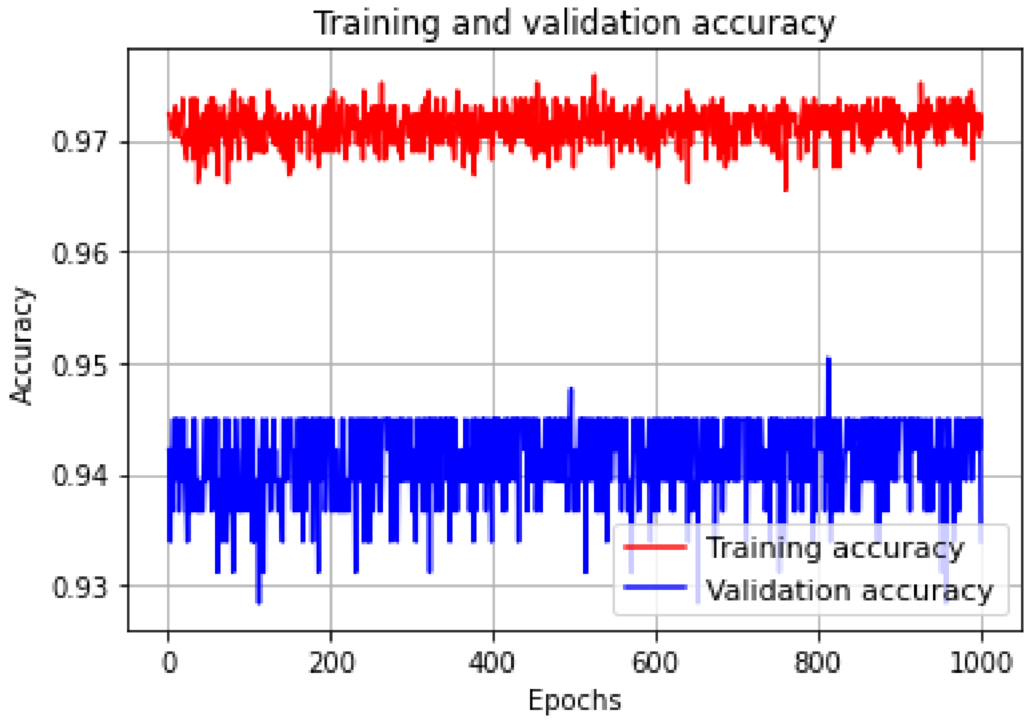

[57]:

```
from sklearn.metrics import confusion_matrix
from sklearn.metrics import classification_report

print("############### Confusion Martix Training Set ###############")

preds = model.predict(X_train)
print(preds[0])
print(np.sum(preds[0]))
matrix = confusion_matrix(dummy_y_train.argmax(axis=1), preds.argmax(axis=1))
matrix
print(classification_report(dummy_y_train.argmax(axis=1), preds.argmax(axis=1)))
matrix
```

```
############### Confusion Martix Training Set ###############
57/57 [==============================] - 0s 2ms/step
[7.2210831e-05 3.2135600e-03 3.5841504e-07 9.9671388e-01 4.7643413e-12
 6.7396009e-09]
1.0
              precision    recall  f1-score   support

           0       0.99      0.85      0.92       302
```

```
           1       0.88      0.97      0.92       302
           2       0.98      0.97      0.97       302
           3       0.96      1.00      0.98       302
           4       0.99      1.00      1.00       302
           5       1.00      0.99      1.00       302

    accuracy                           0.96      1812
   macro avg       0.97      0.96      0.96      1812
weighted avg       0.97      0.96      0.96      1812
```

[57]:
```
array([[258,  32,   6,   4,   2,   0],
       [  0, 293,   0,   9,   0,   0],
       [  0,   9, 293,   0,   0,   0],
       [  0,   0,   0, 302,   0,   0],
       [  0,   0,   0,   0, 302,   0],
       [  2,   0,   1,   0,   0, 299]])
```

[58]:
```python
print("############### Confusion Martix Training Set ###############")
print(classification_report(dummy_y_train.argmax(axis=1), preds.argmax(axis=1)))
matrix
```

```
############### Confusion Martix Training Set ###############
              precision    recall  f1-score   support

           0       0.99      0.85      0.92       302
           1       0.88      0.97      0.92       302
           2       0.98      0.97      0.97       302
           3       0.96      1.00      0.98       302
           4       0.99      1.00      1.00       302
           5       1.00      0.99      1.00       302

    accuracy                           0.96      1812
   macro avg       0.97      0.96      0.96      1812
weighted avg       0.97      0.96      0.96      1812
```

[58]:
```
array([[258,  32,   6,   4,   2,   0],
       [  0, 293,   0,   9,   0,   0],
       [  0,   9, 293,   0,   0,   0],
       [  0,   0,   0, 302,   0,   0],
       [  0,   0,   0,   0, 302,   0],
       [  2,   0,   1,   0,   0, 299]])
```

[59]:
```python
from sklearn.metrics import confusion_matrix
from sklearn.metrics import classification_report

print("############### Confusion Martix Validation Set ###############")
```

```
preds = model.predict(X_validate) # see how the model did!
print(preds[0])
print(np.sum(preds[0]))
matrix = confusion_matrix(dummy_y_validate.argmax(axis=1), preds.argmax(axis=1))
matrix
print(classification_report(dummy_y_validate.argmax(axis=1), preds.
  ↪argmax(axis=1)))
print("Confusion Martix Validation Set")
matrix
```

```
############### Confusion Martix Validation Set ###############
2/2 [==============================] - 0s 5ms/step
[2.1934321e-02 9.6082646e-01 1.7173499e-02 6.5686087e-05 8.8796820e-17
 5.6037817e-11]
1.0
              precision    recall  f1-score   support

           0       1.00      0.93      0.97        15
           1       0.88      1.00      0.93         7
           2       1.00      1.00      1.00         3
           3       1.00      1.00      1.00         2
           4       1.00      1.00      1.00         1
           5       1.00      1.00      1.00        30

    accuracy                           0.98        58
   macro avg       0.98      0.99      0.98        58
weighted avg       0.98      0.98      0.98        58

Confusion Martix Validation Set
```

[59]:
```
array([[14,  1,  0,  0,  0,  0],
       [ 0,  7,  0,  0,  0,  0],
       [ 0,  0,  3,  0,  0,  0],
       [ 0,  0,  0,  2,  0,  0],
       [ 0,  0,  0,  0,  1,  0],
       [ 0,  0,  0,  0,  0, 30]])
```

[60]:
```
from sklearn.metrics import confusion_matrix
from sklearn.metrics import classification_report

print("############### Confusion Martix Test Set ###############")

preds = model.predict(X_test) # see how the model did!
print(preds[0])
print(np.sum(preds[0]))
matrix = confusion_matrix(dummy_y_test.argmax(axis=1), preds.argmax(axis=1))
matrix
print(classification_report(dummy_y_test.argmax(axis=1), preds.argmax(axis=1)))
```

```
matrix
```

```
############### Confusion Martix Test Set ###############
4/4 [==============================] - 0s 2ms/step
[2.6484286e-09 5.1621100e-06 2.1403983e-09 9.9999487e-01 5.4475127e-29
 1.5168123e-12]
1.0
              precision    recall  f1-score   support

           0       1.00      0.90      0.95        31
           1       0.86      0.92      0.89        13
           2       0.88      1.00      0.93         7
           3       0.80      1.00      0.89         4
           4       1.00      1.00      1.00         1
           5       1.00      1.00      1.00        60

    accuracy                           0.97       116
   macro avg       0.92      0.97      0.94       116
weighted avg       0.97      0.97      0.97       116
```

[60]:
```
array([[28,  2,  1,  0,  0,  0],
       [ 0, 12,  0,  1,  0,  0],
       [ 0,  0,  7,  0,  0,  0],
       [ 0,  0,  0,  4,  0,  0],
       [ 0,  0,  0,  0,  1,  0],
       [ 0,  0,  0,  0,  0, 60]])
```
