# Supplementary material for: Survival estimation of oral cancer using fuzzy deep learning
Source: BMC Oral Health. 2024 May 2;24:519. doi: 10.1186/s12903-024-04279-6 (PMC11067185; doi:10.1186/s12903-024-04279-6)
Supplement: Supplementary file 2 — Supplementary Material 2. [file 12903_2024_4279_MOESM2_ESM.pdf]

FuzzyDeepLearningOralCancer_RF

April 17, 2024

[171]:
```
#Importing Libraries
import numpy as np
import pandas as pd
import joblib
from sklearn.model_selection import train_test_split
from sklearn.preprocessing import StandardScaler
from sklearn.ensemble import RandomForestClassifier
from sklearn.metrics import confusion_matrix

# from sklearn.externals import joblib
print('Libraries Imported')
```

```
Libraries Imported
```

[172]:
```
dataset = pd.read_csv("Data_RF_SVM/9.Training_oral_cancer.csv", header = None)
dataset.columns =␣
↪['gender','age_fuzzy','primary_tumor','pT_stage','pN_stage','Staging','Patho','lymph_metast
print('Shape of the dataset: ' + str(dataset.shape))
dataset.head()
```

```
Shape of the dataset: (1253, 14)
```

[172]:
```
   gender  age_fuzzy  primary_tumor  pT_stage  pN_stage  Staging  Patho  \
0       1          3              1         3         3        4      1
1       1          2              1         4         1        4      1
2       2          6              6         2         3        4      1
3       1          3              1         3         6        3      1
4       2          5              1         3         6        3      1

   lymph_metastasis  positive_margin  extranodal_extension  vascular_invasion  \
0                 1                2                     2                  2
1                 1                1                     2                  1
2                 1                2                     2                  2
3                 2                1                     2                  2
4                 2                2                     2                  2

   perineural_invasion  five_year_surv  target
0                    2               1       1
1                    1               1       1
```

```
2                     2                1       1
3                     2                1       1
4                     2                1       1
```

[173]:
```
factor = pd.factorize(dataset['target'])
dataset.target = factor[0]
definitions = factor[1]
print(dataset.target.head())
print(definitions)
```

```
0    0
1    0
2    0
3    0
4    0
Name: target, dtype: int64
Index([1, 2, 3, 4, 5, 6], dtype='int64')
```

[174]:
```
X_train = dataset.iloc[:,0:13].values
y_train = dataset.iloc[:,13].values
print('The independent features set: ')
print(X_train[:14,:])
print('The dependent variable: ')
print(y_train[:14])
```

```
The independent features set:
[[1 3 1 3 3 4 1 1 2 2 2 2 1]
 [1 2 1 4 1 4 1 1 1 2 1 1 1]
 [2 6 6 2 3 4 1 1 2 2 2 2 1]
 [1 3 1 3 6 3 1 2 1 2 2 2 1]
 [2 5 1 3 6 3 1 2 2 2 2 2 1]
 [1 4 2 1 6 1 1 2 2 2 2 2 1]
 [1 2 1 2 6 2 1 2 2 2 2 2 1]
 [2 3 1 3 3 4 1 1 2 1 1 1 1]
 [1 3 1 2 3 4 3 1 1 1 1 1 1]
 [2 4 3 4 6 4 1 2 2 2 2 2 1]
 [2 2 1 4 6 4 2 2 2 2 1 2 1]
 [2 4 1 2 3 4 1 1 2 1 2 2 1]
 [1 3 1 2 3 4 1 1 2 1 1 1 1]
 [2 4 1 2 3 4 1 1 2 1 1 2 1]]
The dependent variable:
[0 0 0 0 0 0 0 0 0 0 0 0 0 0]
```

[175]:
```
dataset_test = pd.read_csv("Data_RF_SVM/5.test_oral_cancer.csv", header = None)
dataset_test.columns =␣
 ↪['gender','age_fuzzy','primary_tumor','pT_stage','pN_stage','Staging','Patho','lymph_metast
print('Shape of the dataset: ' + str(dataset_test.shape))
dataset_test.head()
```

```
Shape of the dataset: (116, 14)
```

[175]:

```
   gender  age_fuzzy  primary_tumor  pT_stage  pN_stage  Staging  Patho  \
0       2          2              4         4         6        4      1
1       1          3              7         3         3        4      2
2       2          3              6         3         6        3      1
3       1          2              7         4         3        4      1
4       1          3              1         3         3        4      2

   lymph_metastasis  positive_margin  extranodal_extension  vascular_invasion  \
0                 2                1                     1                  1
1                 1                2                     2                  2
2                 2                2                     2                  2
3                 1                1                     2                  2
4                 1                2                     2                  2

   perineural_invasion  five_year_surv  target
0                    1               1       1
1                    2               1       1
2                    2               1       1
3                    2               1       1
4                    2               1       1
```

[176]:

```python
factor_test = pd.factorize(dataset_test['target'])
dataset_test.target = factor_test[0]
definitions = factor_test[1]
print(dataset_test.target.head())
print(definitions)
```

```
0    0
1    0
2    0
3    0
4    0
Name: target, dtype: int64
Index([1, 2, 3, 4, 5, 6], dtype='int64')
```

[177]:

```python
X_test = dataset_test.iloc[:,0:13].values
y_test = dataset_test.iloc[:,13].values
print('The independent features set: ')
print(X[:14,:])
print('The dependent variable: ')
print(y[:14])
```

```
The independent features set:
[[5.1 3.5 1.4 0.2]
 [4.9 3.  1.4 0.2]
 [4.7 3.2 1.3 0.2]
 [4.6 3.1 1.5 0.2]
```

```
 [5.  3.6 1.4 0.2]
 [5.4 3.9 1.7 0.4]
 [4.6 3.4 1.4 0.3]
 [5.  3.4 1.5 0.2]
 [4.4 2.9 1.4 0.2]
 [4.9 3.1 1.5 0.1]
 [5.4 3.7 1.5 0.2]
 [4.8 3.4 1.6 0.2]
 [4.8 3.  1.4 0.1]
 [4.3 3.  1.1 0.1]]
The dependent variable:
[[1 0 0 0 0 0]
 [1 0 0 0 0 0]
 [1 0 0 0 0 0]
 [1 0 0 0 0 0]
 [1 0 0 0 0 0]
 [1 0 0 0 0 0]
 [1 0 0 0 0 0]
 [1 0 0 0 0 0]
 [1 0 0 0 0 0]
 [1 0 0 0 0 0]
 [1 0 0 0 0 0]
 [1 0 0 0 0 0]
 [1 0 0 0 0 0]
 [1 0 0 0 0 0]]
```

[178]:
```
from sklearn.preprocessing import StandardScaler
scaler = StandardScaler()
X_train = scaler.fit_transform(X_train)
X_test = scaler.transform(X_test)
```

[179]:
```
classifier = RandomForestClassifier(n_estimators = 10, criterion = 'entropy',␣
  ↪random_state = 42)
classifier.fit(X_train, y_train)
```

[179]:
```
RandomForestClassifier(criterion='entropy', n_estimators=10, random_state=42)
```

[180]:
```
y_pred = classifier.predict(X_test)
reversefactor = dict(zip(range(6),definitions))
print(pd.crosstab(y_test, y_pred, rownames=['Actual Species'],␣
  ↪colnames=['Predicted Species']))
```

```
Predicted Species   0  1  2  3   5
Actual Species
0                  21  6  2  2   0
1                   9  4  0  0   0
2                   6  1  0  0   0
3                   1  3  0  0   0
```

```
4                    1  0  0  0   0
5                    0  0  0  0  60
```

[181]:
```
from sklearn.metrics import confusion_matrix,classification_report
print(classification_report(y_test, y_pred))
```

```
              precision    recall  f1-score   support

           0       0.55      0.68      0.61        31
           1       0.29      0.31      0.30        13
           2       0.00      0.00      0.00         7
           3       0.00      0.00      0.00         4
           4       0.00      0.00      0.00         1
           5       1.00      1.00      1.00        60

    accuracy                           0.73       116
   macro avg       0.31      0.33      0.32       116
weighted avg       0.70      0.73      0.71       116
```

```
/Users/rachasaksomyanonthanakul/anaconda3/lib/python3.11/site-
packages/sklearn/metrics/_classification.py:1344: UndefinedMetricWarning:
Precision and F-score are ill-defined and being set to 0.0 in labels with no
predicted samples. Use `zero_division` parameter to control this behavior.
  _warn_prf(average, modifier, msg_start, len(result))
/Users/rachasaksomyanonthanakul/anaconda3/lib/python3.11/site-
packages/sklearn/metrics/_classification.py:1344: UndefinedMetricWarning:
Precision and F-score are ill-defined and being set to 0.0 in labels with no
predicted samples. Use `zero_division` parameter to control this behavior.
  _warn_prf(average, modifier, msg_start, len(result))
/Users/rachasaksomyanonthanakul/anaconda3/lib/python3.11/site-
packages/sklearn/metrics/_classification.py:1344: UndefinedMetricWarning:
Precision and F-score are ill-defined and being set to 0.0 in labels with no
predicted samples. Use `zero_division` parameter to control this behavior.
  _warn_prf(average, modifier, msg_start, len(result))
```

[185]:
```
y_test
y_test = label_binarize(y_test, classes=[0, 1, 2, 3, 4, 5])
```

[186]:
```
y_test
```

[186]:
```
array([[1, 0, 0, 0, 0, 0],
       [1, 0, 0, 0, 0, 0],
       [1, 0, 0, 0, 0, 0],
       [1, 0, 0, 0, 0, 0],
       [1, 0, 0, 0, 0, 0],
       [1, 0, 0, 0, 0, 0],
       [1, 0, 0, 0, 0, 0],
       [1, 0, 0, 0, 0, 0],
```

```
[1, 0, 0, 0, 0, 0],
[1, 0, 0, 0, 0, 0],
[1, 0, 0, 0, 0, 0],
[1, 0, 0, 0, 0, 0],
[1, 0, 0, 0, 0, 0],
[1, 0, 0, 0, 0, 0],
[1, 0, 0, 0, 0, 0],
[1, 0, 0, 0, 0, 0],
[1, 0, 0, 0, 0, 0],
[1, 0, 0, 0, 0, 0],
[1, 0, 0, 0, 0, 0],
[1, 0, 0, 0, 0, 0],
[1, 0, 0, 0, 0, 0],
[1, 0, 0, 0, 0, 0],
[1, 0, 0, 0, 0, 0],
[1, 0, 0, 0, 0, 0],
[1, 0, 0, 0, 0, 0],
[1, 0, 0, 0, 0, 0],
[1, 0, 0, 0, 0, 0],
[1, 0, 0, 0, 0, 0],
[1, 0, 0, 0, 0, 0],
[1, 0, 0, 0, 0, 0],
[1, 0, 0, 0, 0, 0],
[0, 1, 0, 0, 0, 0],
[0, 1, 0, 0, 0, 0],
[0, 1, 0, 0, 0, 0],
[0, 1, 0, 0, 0, 0],
[0, 1, 0, 0, 0, 0],
[0, 1, 0, 0, 0, 0],
[0, 1, 0, 0, 0, 0],
[0, 1, 0, 0, 0, 0],
[0, 1, 0, 0, 0, 0],
[0, 1, 0, 0, 0, 0],
[0, 1, 0, 0, 0, 0],
[0, 1, 0, 0, 0, 0],
[0, 1, 0, 0, 0, 0],
[0, 0, 1, 0, 0, 0],
[0, 0, 1, 0, 0, 0],
[0, 0, 1, 0, 0, 0],
[0, 0, 1, 0, 0, 0],
[0, 0, 1, 0, 0, 0],
[0, 0, 1, 0, 0, 0],
[0, 0, 1, 0, 0, 0],
[0, 0, 0, 1, 0, 0],
[0, 0, 0, 1, 0, 0],
[0, 0, 0, 1, 0, 0],
[0, 0, 0, 1, 0, 0],
```

```
[0, 0, 0, 0, 1, 0],
[0, 0, 0, 0, 0, 1],
[0, 0, 0, 0, 0, 1],
[0, 0, 0, 0, 0, 1],
[0, 0, 0, 0, 0, 1],
[0, 0, 0, 0, 0, 1],
[0, 0, 0, 0, 0, 1],
[0, 0, 0, 0, 0, 1],
[0, 0, 0, 0, 0, 1],
[0, 0, 0, 0, 0, 1],
[0, 0, 0, 0, 0, 1],
[0, 0, 0, 0, 0, 1],
[0, 0, 0, 0, 0, 1],
[0, 0, 0, 0, 0, 1],
[0, 0, 0, 0, 0, 1],
[0, 0, 0, 0, 0, 1],
[0, 0, 0, 0, 0, 1],
[0, 0, 0, 0, 0, 1],
[0, 0, 0, 0, 0, 1],
[0, 0, 0, 0, 0, 1],
[0, 0, 0, 0, 0, 1],
[0, 0, 0, 0, 0, 1],
[0, 0, 0, 0, 0, 1],
[0, 0, 0, 0, 0, 1],
[0, 0, 0, 0, 0, 1],
[0, 0, 0, 0, 0, 1],
[0, 0, 0, 0, 0, 1],
[0, 0, 0, 0, 0, 1],
[0, 0, 0, 0, 0, 1],
[0, 0, 0, 0, 0, 1],
[0, 0, 0, 0, 0, 1],
[0, 0, 0, 0, 0, 1],
[0, 0, 0, 0, 0, 1],
[0, 0, 0, 0, 0, 1],
[0, 0, 0, 0, 0, 1],
[0, 0, 0, 0, 0, 1],
[0, 0, 0, 0, 0, 1],
[0, 0, 0, 0, 0, 1],
[0, 0, 0, 0, 0, 1],
[0, 0, 0, 0, 0, 1],
[0, 0, 0, 0, 0, 1],
[0, 0, 0, 0, 0, 1],
[0, 0, 0, 0, 0, 1],
[0, 0, 0, 0, 0, 1],
[0, 0, 0, 0, 0, 1],
[0, 0, 0, 0, 0, 1],
[0, 0, 0, 0, 0, 1],
```

```
       [0, 0, 0, 0, 0, 1],
       [0, 0, 0, 0, 0, 1],
       [0, 0, 0, 0, 0, 1],
       [0, 0, 0, 0, 0, 1],
       [0, 0, 0, 0, 0, 1],
       [0, 0, 0, 0, 0, 1],
       [0, 0, 0, 0, 0, 1],
       [0, 0, 0, 0, 0, 1],
       [0, 0, 0, 0, 0, 1],
       [0, 0, 0, 0, 0, 1],
       [0, 0, 0, 0, 0, 1],
       [0, 0, 0, 0, 0, 1],
       [0, 0, 0, 0, 0, 1],
       [0, 0, 0, 0, 0, 1]])
```

[188]:
```
y_pred = label_binarize(y_pred, classes=[0, 1, 2, 3, 4, 5])
y_score = y_pred
y_score
```

[188]:
```
array([[1, 0, 0, 0, 0, 0],
       [0, 0, 0, 1, 0, 0],
       [1, 0, 0, 0, 0, 0],
       [1, 0, 0, 0, 0, 0],
       [1, 0, 0, 0, 0, 0],
       [1, 0, 0, 0, 0, 0],
       [0, 1, 0, 0, 0, 0],
       [1, 0, 0, 0, 0, 0],
       [1, 0, 0, 0, 0, 0],
       [0, 0, 1, 0, 0, 0],
       [1, 0, 0, 0, 0, 0],
       [1, 0, 0, 0, 0, 0],
       [1, 0, 0, 0, 0, 0],
       [1, 0, 0, 0, 0, 0],
       [0, 1, 0, 0, 0, 0],
       [1, 0, 0, 0, 0, 0],
       [1, 0, 0, 0, 0, 0],
       [0, 1, 0, 0, 0, 0],
       [1, 0, 0, 0, 0, 0],
       [1, 0, 0, 0, 0, 0],
       [1, 0, 0, 0, 0, 0],
       [1, 0, 0, 0, 0, 0],
       [0, 1, 0, 0, 0, 0],
       [0, 0, 1, 0, 0, 0],
       [0, 1, 0, 0, 0, 0],
       [1, 0, 0, 0, 0, 0],
       [1, 0, 0, 0, 0, 0],
       [1, 0, 0, 0, 0, 0],
```

```
[0, 1, 0, 0, 0, 0],
[0, 0, 0, 1, 0, 0],
[1, 0, 0, 0, 0, 0],
[0, 1, 0, 0, 0, 0],
[1, 0, 0, 0, 0, 0],
[1, 0, 0, 0, 0, 0],
[0, 1, 0, 0, 0, 0],
[1, 0, 0, 0, 0, 0],
[0, 1, 0, 0, 0, 0],
[1, 0, 0, 0, 0, 0],
[1, 0, 0, 0, 0, 0],
[1, 0, 0, 0, 0, 0],
[1, 0, 0, 0, 0, 0],
[0, 1, 0, 0, 0, 0],
[1, 0, 0, 0, 0, 0],
[1, 0, 0, 0, 0, 0],
[0, 1, 0, 0, 0, 0],
[1, 0, 0, 0, 0, 0],
[1, 0, 0, 0, 0, 0],
[1, 0, 0, 0, 0, 0],
[1, 0, 0, 0, 0, 0],
[1, 0, 0, 0, 0, 0],
[1, 0, 0, 0, 0, 0],
[0, 1, 0, 0, 0, 0],
[0, 1, 0, 0, 0, 0],
[0, 1, 0, 0, 0, 0],
[1, 0, 0, 0, 0, 0],
[1, 0, 0, 0, 0, 0],
[0, 0, 0, 0, 0, 1],
[0, 0, 0, 0, 0, 1],
[0, 0, 0, 0, 0, 1],
[0, 0, 0, 0, 0, 1],
[0, 0, 0, 0, 0, 1],
[0, 0, 0, 0, 0, 1],
[0, 0, 0, 0, 0, 1],
[0, 0, 0, 0, 0, 1],
[0, 0, 0, 0, 0, 1],
[0, 0, 0, 0, 0, 1],
[0, 0, 0, 0, 0, 1],
[0, 0, 0, 0, 0, 1],
[0, 0, 0, 0, 0, 1],
[0, 0, 0, 0, 0, 1],
[0, 0, 0, 0, 0, 1],
[0, 0, 0, 0, 0, 1],
[0, 0, 0, 0, 0, 1],
[0, 0, 0, 0, 0, 1],
[0, 0, 0, 0, 0, 1],
```

```
       [0, 0, 0, 0, 0, 1],
       [0, 0, 0, 0, 0, 1],
       [0, 0, 0, 0, 0, 1],
       [0, 0, 0, 0, 0, 1],
       [0, 0, 0, 0, 0, 1],
       [0, 0, 0, 0, 0, 1],
       [0, 0, 0, 0, 0, 1],
       [0, 0, 0, 0, 0, 1],
       [0, 0, 0, 0, 0, 1],
       [0, 0, 0, 0, 0, 1],
       [0, 0, 0, 0, 0, 1],
       [0, 0, 0, 0, 0, 1],
       [0, 0, 0, 0, 0, 1],
       [0, 0, 0, 0, 0, 1],
       [0, 0, 0, 0, 0, 1],
       [0, 0, 0, 0, 0, 1],
       [0, 0, 0, 0, 0, 1],
       [0, 0, 0, 0, 0, 1],
       [0, 0, 0, 0, 0, 1],
       [0, 0, 0, 0, 0, 1],
       [0, 0, 0, 0, 0, 1],
       [0, 0, 0, 0, 0, 1],
       [0, 0, 0, 0, 0, 1],
       [0, 0, 0, 0, 0, 1],
       [0, 0, 0, 0, 0, 1],
       [0, 0, 0, 0, 0, 1],
       [0, 0, 0, 0, 0, 1],
       [0, 0, 0, 0, 0, 1],
       [0, 0, 0, 0, 0, 1],
       [0, 0, 0, 0, 0, 1],
       [0, 0, 0, 0, 0, 1],
       [0, 0, 0, 0, 0, 1],
       [0, 0, 0, 0, 0, 1],
       [0, 0, 0, 0, 0, 1],
       [0, 0, 0, 0, 0, 1],
       [0, 0, 0, 0, 0, 1],
       [0, 0, 0, 0, 0, 1],
       [0, 0, 0, 0, 0, 1],
       [0, 0, 0, 0, 0, 1],
       [0, 0, 0, 0, 0, 1],
       [0, 0, 0, 0, 0, 1]])
```

[189]:
```
n_classes = y_score.shape[1]
n_classes
```

[189]: 6

[221]:

```
import numpy as np
from scipy import interp
import matplotlib.pyplot as plt
from itertools import cycle
from sklearn.metrics import roc_curve, auc

n_classes = 6
lw = 2
fpr = dict()
tpr = dict()
roc_auc = dict()
for i in range(n_classes):
    fpr[i], tpr[i], _ = roc_curve(y_test[:, i], y_score[:, i])
    #fpr[i], tpr[i], _ = roc_curve(y_test[:, i], y_score[:, i])
    roc_auc[i] = auc(fpr[i], tpr[i])

fpr["micro"], tpr["micro"], _ = roc_curve(y_test.ravel(), y_score.ravel())
roc_auc["micro"] = auc(fpr["micro"], tpr["micro"])

all_fpr = np.unique(np.concatenate([fpr[i] for i in range(n_classes)]))

mean_tpr = np.zeros_like(all_fpr)
for i in range(n_classes):
    mean_tpr += np.interp(all_fpr, fpr[i], tpr[i])

# Finally average it and compute AUC
mean_tpr /= n_classes

fpr["macro"] = all_fpr
tpr["macro"] = mean_tpr
roc_auc["macro"] = auc(fpr["macro"], tpr["macro"])

# Plot all ROC curves
plt.figure(1)
plt.grid(True)
plt.plot(fpr["micro"], tpr["micro"],
         label='micro-average ROC curve (area = {0:0.2f})'
               ''.format(roc_auc["micro"]),
         color='deeppink', linestyle=':', linewidth=4)

plt.plot(fpr["macro"], tpr["macro"],
         label='macro-average ROC curve (area = {0:0.2f})'
               ''.format(roc_auc["macro"]),
         color='navy', linestyle=':', linewidth=4)

colors = cycle(['aqua', 'darkorange', 'cornflowerblue','red', 'yellow', 'blue'])
for i, color in zip(range(n_classes), colors):
```

```
    plt.plot(fpr[i], tpr[i], color=color, lw=lw,
             label='ROC curve of class {0} (area = {1:0.2f})'
             ''.format(i, roc_auc[i]))

plt.plot([0, 1], [0, 1], 'k--', lw=lw)
plt.xlim([0.0, 1.0])
plt.ylim([0.0, 1.05])
plt.xlabel('False Positive Rate')
plt.ylabel('True Positive Rate')
plt.title('Some extension of Receiver operating characteristic to multi-class')
plt.legend(loc="lower right")
plt.savefig('ROC_RF_1.tiff')
plt.show()


# Zoom in view of the upper left corner.
plt.figure(2)

plt.grid(True)

plt.xlim(0, 0.2)
plt.ylim(0.8, 1)
plt.plot(fpr["micro"], tpr["micro"],
         label='micro-average ROC curve (area = {0:0.2f})'
               ''.format(roc_auc["micro"]),
         color='deeppink', linestyle=':', linewidth=4)

plt.plot(fpr["macro"], tpr["macro"],
         label='macro-average ROC curve (area = {0:0.2f})'
               ''.format(roc_auc["macro"]),
         color='navy', linestyle=':', linewidth=4)

colors = cycle(['aqua', 'darkorange', 'cornflowerblue','red', 'yellow', 'blue'])
for i, color in zip(range(n_classes), colors):
    plt.plot(fpr[i], tpr[i], color=color, lw=lw,
             label='ROC curve of class {0} (area = {1:0.2f})'
             ''.format(i, roc_auc[i]))

plt.plot([0, 1], [0, 1], 'k--', lw=lw)
plt.xlabel('False Positive Rate')
plt.ylabel('True Positive Rate')
plt.title('Some extension of Receiver operating characteristic to multi-class')
plt.legend(loc="lower right")
plt.savefig('ROC_RF_2.pdf')
plt.show()
```

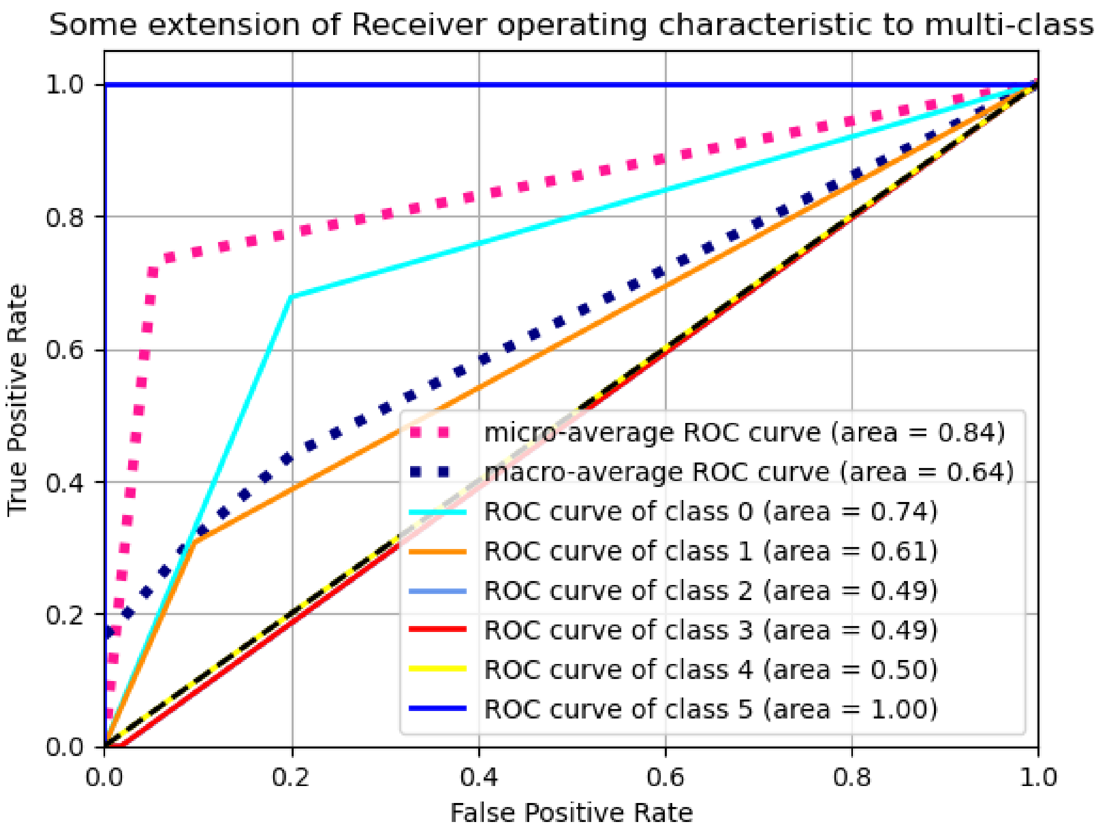

Some extension of Receiver operating characteristic to multi-class
True Positive Rate
False Positive Rate
0.0
0.2
0.4
0.6
0.8
1.0
micro-average ROC curve (area = 0.84)
macro-average ROC curve (area = 0.64)
ROC curve of class 0 (area = 0.74)
ROC curve of class 1 (area = 0.61)
ROC curve of class 2 (area = 0.49)
ROC curve of class 3 (area = 0.49)
ROC curve of class 4 (area = 0.50)
ROC curve of class 5 (area = 1.00)

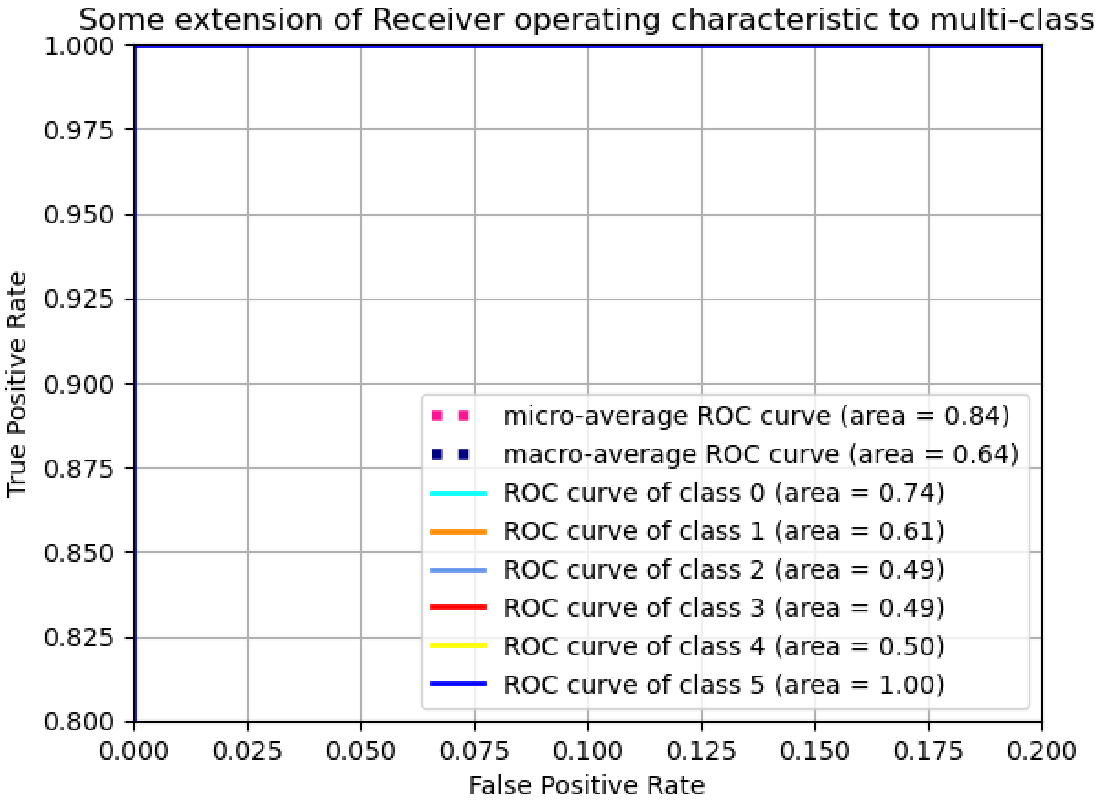
Some extension of Receiver operating characteristic to multi-class
1.000
0.975
0.950
0.925
0.900
0.875
0.850
0.825
0.800
True Positive Rate
micro-average ROC curve (area = 0.84)
macro-average ROC curve (area = 0.64)
ROC curve of class 0 (area = 0.74)
ROC curve of class 1 (area = 0.61)
ROC curve of class 2 (area = 0.49)
ROC curve of class 3 (area = 0.49)
ROC curve of class 4 (area = 0.50)
ROC curve of class 5 (area = 1.00)
0.000
0.025
0.050
0.075
0.100
0.125
0.150
0.175
0.200
False Positive Rate

```
[ ]:
```
