# Supplementary material for: Survival estimation of oral cancer using fuzzy deep learning
Source: BMC Oral Health. 2024 May 2;24:519. doi: 10.1186/s12903-024-04279-6 (PMC11067185; doi:10.1186/s12903-024-04279-6)
Supplement: Supplementary file 3 — Supplementary Material 3. [file 12903_2024_4279_MOESM3_ESM.pdf]

FuzzyDeepLearningOralCancer_SVM

April 17, 2024

[66]:
```
import numpy as np
import pylab as pl
import pandas as pd
import matplotlib.pyplot as plt
%matplotlib inline
import seaborn as sns
from sklearn.utils import shuffle
from sklearn.svm import SVC
from sklearn.metrics import confusion_matrix,classification_report
from sklearn.model_selection import cross_val_score, GridSearchCV

import os
print(os.listdir("Data_RF_SVM/"))
```

```
['.DS_Store', 'Convert_Label.xlsx', 'test.csv',
'9.Training_oral_cancer_1253_Fuzzy.csv', 'lung.csv',
'9.Training_oral_cancer_1253_Fuzzy_no_title.csv',
'4.validate_oral_cancer_58.csv', 'iris.csv', '5.test_oral_cancer_116.csv',
'iris_out.csv', '5.test_oral_cancer_116_no_title.csv', 'train.csv',
'9.Training_oral_cancer_1253_Fuzzy_cox.csv', 'heart_v2.csv',
'original_data_svm', 'iris_v01.csv']
```

[67]:
```
train = shuffle(pd.read_csv("Data_RF_SVM/train.csv"))
test = shuffle(pd.read_csv("Data_RF_SVM/test.csv"))
```

[68]:
```
print("Any missing sample in training set:",train.isnull().values.any())
print("Any missing sample in test set:",test.isnull().values.any(), "\n")
```

```
Any missing sample in training set: False
Any missing sample in test set: False
```

[69]:
```
#Frequency distribution of classes"
train_outcome = pd.crosstab(index=train["Activity"],  # Make a crosstab
                            columns="count")      # Name the count column

train_outcome
```

[69]:
```
col_0     count
Activity
A           213
B           210
C           210
D           207
E           201
F           212
```

[70]:
```
# Visualizing Outcome Distribution
temp = train["Activity"].value_counts()
df = pd.DataFrame({'labels': temp.index,
                   'values': temp.values
                  })

#df.plot(kind='pie',labels='labels',values='values', title='Activity␣
↪Ditribution',subplots= "True")

labels = df['labels']
sizes = df['values']
colors = ['yellowgreen', 'gold', 'lightskyblue',␣
↪'lightcoral','cyan','lightpink']
patches, texts = plt.pie(sizes, colors=colors, shadow=True, startangle=90,␣
↪pctdistance=1.1, labeldistance=1.2)
plt.legend(patches, labels, loc="best")
plt.axis('equal')
plt.tight_layout()
plt.show()
```

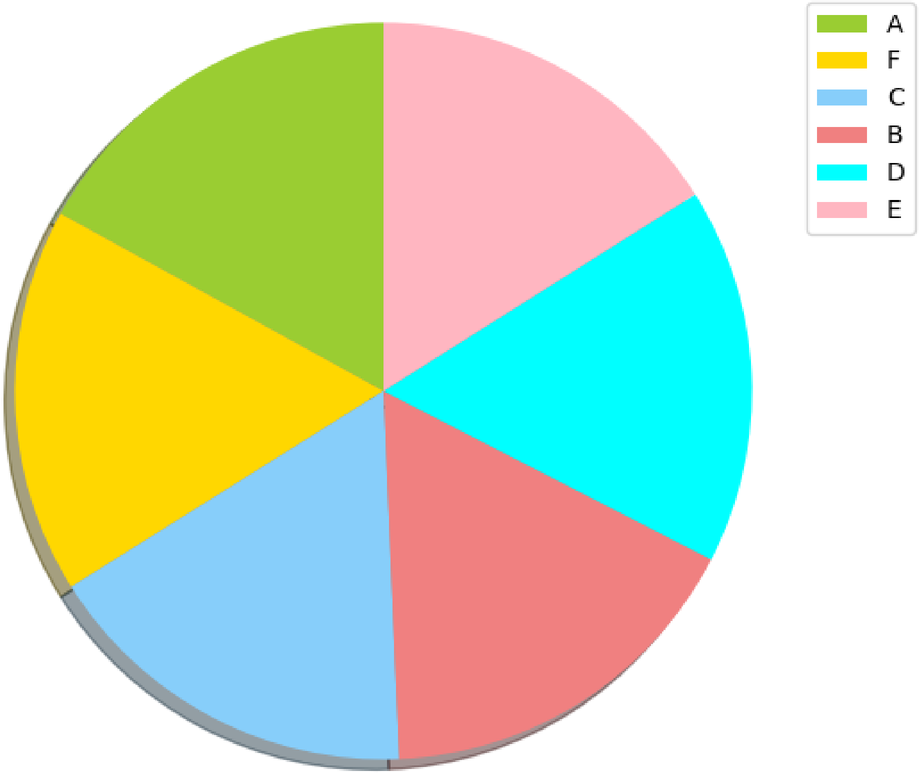

[71]:

```
X_train = pd.DataFrame(train.drop(['Activity','subject'],axis=1))
Y_train_label = train.Activity.values.astype(object)


X_test = pd.DataFrame(test.drop(['Activity','subject'],axis=1))
Y_test_label = test.Activity.values.astype(object)

print("Dimension of Train set",X_train.shape)
print("Dimension of Test set",X_test.shape,"\n")

from sklearn import preprocessing
encoder = preprocessing.LabelEncoder()

encoder.fit(Y_train_label)
Y_train = encoder.transform(Y_train_label)

encoder.fit(Y_test_label)
Y_test = encoder.transform(Y_test_label)

num_cols = X_train._get_numeric_data().columns
```

```python
print("Number of numeric features:",num_cols.size)
names_of_predictors = list(X_train.columns.values)

from sklearn.preprocessing import StandardScaler
scaler = StandardScaler()
X_train_scaled = scaler.fit_transform(X_train)
X_test_scaled = scaler.transform(X_test)
```

```
Dimension of Train set (1253, 12)
Dimension of Test set (116, 12)

Number of numeric features: 12
```

[55]:
```python
np.savetxt("SVM_Y_test.csv", Y_test, delimiter=",")
```

[72]:
```python
X_test_scaled
```

[72]:
```
array([[ 0.97400106, -1.56803019,  0.8486309 , …, -3.57340605,
        -2.29455878, -1.60623784],
       [ 0.97400106, -0.76935465,  0.8486309 , …,  0.27984505,
         0.43581363,  0.62257281],
       [ 0.97400106, -0.76935465,  0.24549164, …,  0.27984505,
         0.43581363,  0.62257281],
       …,
       [ 0.97400106, -0.76935465,  0.8486309 , …,  0.27984505,
        -2.29455878,  0.62257281],
       [-1.02669292, -0.76935465, -0.96078688, …,  0.27984505,
         0.43581363,  0.62257281],
       [ 0.97400106, -0.76935465,  2.05490941, …,  0.27984505,
         0.43581363,  0.62257281]])
```

[73]:
```python
params_grid = [{'kernel': ['rbf'], 'gamma': [1e-3, 1e-4],
                     'C': [1, 10, 100, 1000]},
                    {'kernel': ['linear'], 'C': [1, 10, 100, 1000]}]
```

[74]:
```python
# Performing CV to tune parameters for best SVM fit
svm_model = GridSearchCV(SVC(), params_grid, cv=5)
svm_model.fit(X_train_scaled, Y_train)
```

[74]:
```
GridSearchCV(cv=5, estimator=SVC(),
             param_grid=[{'C': [1, 10, 100, 1000], 'gamma': [0.001, 0.0001],
                          'kernel': ['rbf']},
                         {'C': [1, 10, 100, 1000], 'kernel': ['linear']}])
```

[75]:
```python
# View the accuracy score
print('Best score for training data:', svm_model.best_score_,"\n")

# View the best parameters for the model found using grid search
```

```python
print('Best C:',svm_model.best_estimator_.C,"\n")
print('Best Kernel:',svm_model.best_estimator_.kernel,"\n")
print('Best Gamma:',svm_model.best_estimator_.gamma,"\n")

final_model = svm_model.best_estimator_
Y_pred = final_model.predict(X_test_scaled)
Y_pred_label = list(encoder.inverse_transform(Y_pred))
```

```
Best score for training data: 0.5985402390438248

Best C: 1000

Best Kernel: rbf

Best Gamma: 0.001
```

[76]:
```python
np.savetxt("SVM_Y_pred.csv", Y_pred, delimiter=",")
```

[77]:
```python
print(confusion_matrix(Y_test_label,Y_pred_label))
print("\n")
print(classification_report(Y_test_label,Y_pred_label))

print("Training set score for SVM: %f" % final_model.score(X_train_scaled ,␣
    ↪Y_train))
print("Testing  set score for SVM: %f" % final_model.score(X_test_scaled  ,␣
    ↪Y_test ))

svm_model.score
```

```
[[ 8 11  5  1  0  6]
 [ 0  6  1  0  0  6]
 [ 3  1  1  1  0  1]
 [ 0  3  0  1  0  0]
 [ 0  1  0  0  0  0]
 [ 4 25  2  5  3 21]]


              precision    recall  f1-score   support

           A       0.53      0.26      0.35        31
           B       0.13      0.46      0.20        13
           C       0.11      0.14      0.12         7
           D       0.12      0.25      0.17         4
           E       0.00      0.00      0.00         1
           F       0.62      0.35      0.45        60

    accuracy                           0.32       116
```

```
   macro avg       0.25      0.24      0.21       116
weighted avg       0.49      0.32      0.36       116

Training set score for SVM: 0.655227
Testing  set score for SVM: 0.318966
```

[77]:
```
<bound method BaseSearchCV.score of GridSearchCV(cv=5, estimator=SVC(),
             param_grid=[{'C': [1, 10, 100, 1000], 'gamma': [0.001, 0.0001],
                          'kernel': ['rbf']},
                         {'C': [1, 10, 100, 1000], 'kernel': ['linear']}])>
```

[83]:
```
Y_test = label_binarize(Y_test, classes=[0, 1, 2, 3, 4, 5])
y_test = Y_test
y_test
```

[83]:
```
array([[1, 0, 0, 0, 0, 0],
       [1, 0, 0, 0, 0, 0],
       [0, 0, 0, 0, 0, 1],
       [0, 0, 0, 0, 0, 1],
       [0, 0, 0, 0, 0, 1],
       [0, 0, 0, 0, 0, 1],
       [1, 0, 0, 0, 0, 0],
       [1, 0, 0, 0, 0, 0],
       [0, 0, 0, 0, 0, 1],
       [0, 0, 0, 0, 0, 1],
       [0, 0, 0, 0, 0, 1],
       [1, 0, 0, 0, 0, 0],
       [0, 0, 1, 0, 0, 0],
       [0, 0, 0, 0, 0, 1],
       [0, 0, 0, 0, 0, 1],
       [0, 0, 0, 0, 0, 1],
       [1, 0, 0, 0, 0, 0],
       [0, 1, 0, 0, 0, 0],
       [0, 1, 0, 0, 0, 0],
       [0, 0, 0, 0, 0, 1],
       [0, 0, 0, 0, 0, 1],
       [0, 0, 0, 0, 0, 1],
       [0, 0, 0, 0, 0, 1],
       [0, 1, 0, 0, 0, 0],
       [1, 0, 0, 0, 0, 0],
       [1, 0, 0, 0, 0, 0],
       [1, 0, 0, 0, 0, 0],
       [0, 0, 0, 0, 0, 1],
       [0, 0, 0, 0, 0, 1],
       [0, 0, 1, 0, 0, 0],
       [0, 0, 0, 1, 0, 0],
       [0, 0, 0, 0, 0, 1],
       [1, 0, 0, 0, 0, 0],
```

```
[0, 0, 0, 0, 0, 1],
[0, 0, 0, 0, 0, 1],
[1, 0, 0, 0, 0, 0],
[0, 0, 0, 0, 0, 1],
[0, 1, 0, 0, 0, 0],
[0, 0, 0, 0, 0, 1],
[0, 1, 0, 0, 0, 0],
[0, 0, 0, 0, 0, 1],
[0, 0, 0, 0, 0, 1],
[1, 0, 0, 0, 0, 0],
[0, 0, 0, 0, 0, 1],
[0, 0, 0, 0, 0, 1],
[1, 0, 0, 0, 0, 0],
[0, 0, 0, 0, 0, 1],
[0, 0, 0, 0, 0, 1],
[0, 0, 0, 0, 0, 1],
[1, 0, 0, 0, 0, 0],
[0, 0, 0, 0, 0, 1],
[1, 0, 0, 0, 0, 0],
[0, 0, 0, 0, 0, 1],
[1, 0, 0, 0, 0, 0],
[0, 0, 0, 0, 0, 1],
[0, 1, 0, 0, 0, 0],
[0, 0, 1, 0, 0, 0],
[0, 0, 0, 0, 0, 1],
[0, 0, 0, 0, 0, 1],
[1, 0, 0, 0, 0, 0],
[0, 0, 0, 0, 0, 1],
[0, 0, 0, 0, 0, 1],
[0, 0, 0, 0, 0, 1],
[0, 0, 0, 0, 0, 1],
[0, 0, 0, 0, 0, 1],
[0, 1, 0, 0, 0, 0],
[0, 0, 0, 1, 0, 0],
[0, 0, 0, 0, 0, 1],
[0, 0, 0, 0, 0, 1],
[1, 0, 0, 0, 0, 0],
[0, 0, 0, 0, 0, 1],
[0, 1, 0, 0, 0, 0],
[0, 0, 0, 0, 0, 1],
[0, 1, 0, 0, 0, 0],
[0, 0, 1, 0, 0, 0],
[0, 0, 0, 0, 0, 1],
[1, 0, 0, 0, 0, 0],
[0, 0, 0, 0, 0, 1],
[0, 0, 0, 1, 0, 0],
[0, 0, 0, 0, 0, 1],
```

```
         [1, 0, 0, 0, 0, 0],
         [0, 0, 0, 0, 0, 1],
         [0, 1, 0, 0, 0, 0],
         [1, 0, 0, 0, 0, 0],
         [0, 0, 0, 1, 0, 0],
         [0, 1, 0, 0, 0, 0],
         [0, 1, 0, 0, 0, 0],
         [0, 0, 1, 0, 0, 0],
         [0, 0, 0, 0, 0, 1],
         [0, 0, 1, 0, 0, 0],
         [1, 0, 0, 0, 0, 0],
         [0, 0, 0, 0, 0, 1],
         [0, 0, 0, 0, 0, 1],
         [1, 0, 0, 0, 0, 0],
         [0, 0, 0, 0, 1, 0],
         [1, 0, 0, 0, 0, 0],
         [0, 0, 0, 0, 0, 1],
         [0, 0, 0, 0, 0, 1],
         [0, 0, 0, 0, 0, 1],
         [0, 0, 0, 0, 0, 1],
         [1, 0, 0, 0, 0, 0],
         [0, 0, 0, 0, 0, 1],
         [0, 1, 0, 0, 0, 0],
         [1, 0, 0, 0, 0, 0],
         [1, 0, 0, 0, 0, 0],
         [0, 0, 0, 0, 0, 1],
         [0, 0, 0, 0, 0, 1],
         [1, 0, 0, 0, 0, 0],
         [1, 0, 0, 0, 0, 0],
         [0, 0, 0, 0, 0, 1],
         [0, 0, 0, 0, 0, 1],
         [0, 0, 0, 0, 0, 1],
         [0, 0, 1, 0, 0, 0],
         [0, 0, 0, 0, 0, 1],
         [1, 0, 0, 0, 0, 0],
         [1, 0, 0, 0, 0, 0]])
```

[85]:
```
y_score = label_binarize(Y_pred, classes=[0, 1, 2, 3, 4, 5])
y_score
```

[85]:
```
array([[1, 0, 0, 0, 0, 0],
       [0, 1, 0, 0, 0, 0],
       [0, 0, 0, 0, 0, 1],
       [0, 1, 0, 0, 0, 0],
       [0, 0, 0, 0, 0, 1],
       [0, 1, 0, 0, 0, 0],
       [0, 0, 0, 1, 0, 0],
```

```
[0, 0, 0, 0, 0, 1],
[0, 1, 0, 0, 0, 0],
[0, 0, 0, 0, 0, 1],
[0, 1, 0, 0, 0, 0],
[0, 1, 0, 0, 0, 0],
[1, 0, 0, 0, 0, 0],
[0, 0, 0, 0, 0, 1],
[0, 0, 0, 0, 0, 1],
[0, 0, 0, 0, 0, 1],
[1, 0, 0, 0, 0, 0],
[0, 0, 0, 0, 0, 1],
[0, 1, 0, 0, 0, 0],
[0, 0, 0, 0, 1, 0],
[0, 0, 0, 0, 0, 1],
[0, 1, 0, 0, 0, 0],
[0, 1, 0, 0, 0, 0],
[0, 1, 0, 0, 0, 0],
[0, 0, 0, 0, 0, 1],
[1, 0, 0, 0, 0, 0],
[1, 0, 0, 0, 0, 0],
[0, 1, 0, 0, 0, 0],
[0, 0, 0, 1, 0, 0],
[1, 0, 0, 0, 0, 0],
[0, 1, 0, 0, 0, 0],
[0, 0, 1, 0, 0, 0],
[0, 0, 1, 0, 0, 0],
[0, 0, 0, 0, 0, 1],
[0, 0, 0, 0, 1, 0],
[0, 0, 1, 0, 0, 0],
[0, 0, 0, 0, 0, 1],
[0, 0, 0, 0, 0, 1],
[0, 0, 0, 1, 0, 0],
[0, 0, 1, 0, 0, 0],
[0, 1, 0, 0, 0, 0],
[0, 0, 0, 0, 0, 1],
[0, 1, 0, 0, 0, 0],
[0, 1, 0, 0, 0, 0],
[0, 1, 0, 0, 0, 0],
[1, 0, 0, 0, 0, 0],
[0, 0, 0, 0, 0, 1],
[0, 1, 0, 0, 0, 0],
[1, 0, 0, 0, 0, 0],
[0, 0, 1, 0, 0, 0],
[0, 1, 0, 0, 0, 0],
[0, 0, 0, 0, 0, 1],
[0, 0, 0, 1, 0, 0],
[0, 1, 0, 0, 0, 0],
```

```
[1, 0, 0, 0, 0, 0],
[0, 0, 0, 0, 0, 1],
[0, 1, 0, 0, 0, 0],
[0, 1, 0, 0, 0, 0],
[1, 0, 0, 0, 0, 0],
[0, 1, 0, 0, 0, 0],
[0, 0, 0, 0, 0, 1],
[0, 0, 0, 0, 0, 1],
[0, 1, 0, 0, 0, 0],
[0, 0, 0, 0, 0, 1],
[0, 1, 0, 0, 0, 0],
[0, 1, 0, 0, 0, 0],
[0, 0, 0, 1, 0, 0],
[0, 0, 0, 1, 0, 0],
[0, 1, 0, 0, 0, 0],
[0, 1, 0, 0, 0, 0],
[0, 1, 0, 0, 0, 0],
[0, 1, 0, 0, 0, 0],
[0, 0, 0, 0, 0, 1],
[0, 1, 0, 0, 0, 0],
[0, 0, 0, 0, 0, 1],
[0, 1, 0, 0, 0, 0],
[1, 0, 0, 0, 0, 0],
[0, 1, 0, 0, 0, 0],
[0, 1, 0, 0, 0, 0],
[0, 0, 0, 0, 1, 0],
[0, 1, 0, 0, 0, 0],
[1, 0, 0, 0, 0, 0],
[0, 0, 0, 0, 0, 1],
[0, 0, 0, 0, 0, 1],
[0, 1, 0, 0, 0, 0],
[0, 0, 0, 0, 0, 1],
[0, 0, 0, 0, 0, 1],
[0, 0, 1, 0, 0, 0],
[0, 1, 0, 0, 0, 0],
[1, 0, 0, 0, 0, 0],
[0, 1, 0, 0, 0, 0],
[0, 0, 0, 0, 0, 1],
[0, 0, 1, 0, 0, 0],
[0, 0, 0, 0, 0, 1],
[0, 1, 0, 0, 0, 0],
[0, 0, 0, 0, 0, 1],
[0, 0, 0, 1, 0, 0],
[0, 0, 0, 0, 0, 1],
[0, 1, 0, 0, 0, 0],
[0, 0, 0, 0, 0, 1],
[0, 1, 0, 0, 0, 0],
```

```
        [0, 0, 0, 0, 0, 1],
        [0, 1, 0, 0, 0, 0],
        [0, 1, 0, 0, 0, 0],
        [1, 0, 0, 0, 0, 0],
        [0, 1, 0, 0, 0, 0],
        [0, 1, 0, 0, 0, 0],
        [0, 0, 1, 0, 0, 0],
        [1, 0, 0, 0, 0, 0],
        [0, 1, 0, 0, 0, 0],
        [0, 1, 0, 0, 0, 0],
        [0, 0, 0, 0, 0, 1],
        [0, 0, 0, 1, 0, 0],
        [0, 0, 0, 0, 0, 1],
        [0, 0, 1, 0, 0, 0],
        [0, 1, 0, 0, 0, 0]])
```

[88]:

```
import numpy as np
from scipy import interp
import matplotlib.pyplot as plt
from itertools import cycle
from sklearn.metrics import roc_curve, auc

n_classes = 6
lw = 2

# Compute ROC curve and ROC area for each class
fpr = dict()
tpr = dict()
roc_auc = dict()
for i in range(n_classes):
    fpr[i], tpr[i], _ = roc_curve(y_test[:, i], y_score[:, i])
    #fpr[i], tpr[i], _ = roc_curve(y_test[:, i], y_score[:, i])
    roc_auc[i] = auc(fpr[i], tpr[i])

# Compute micro-average ROC curve and ROC area
#fpr["micro"], tpr["micro"], _ = roc_curve(y_test.ravel(), y_score.ravel())
fpr["micro"], tpr["micro"], _ = roc_curve(y_test.ravel(), y_score.ravel())
roc_auc["micro"] = auc(fpr["micro"], tpr["micro"])

# Compute macro-average ROC curve and ROC area

# First aggregate all false positive rates
all_fpr = np.unique(np.concatenate([fpr[i] for i in range(n_classes)]))

# Then interpolate all ROC curves at this points
mean_tpr = np.zeros_like(all_fpr)
for i in range(n_classes):
```

```
    mean_tpr += np.interp(all_fpr, fpr[i], tpr[i])

# Finally average it and compute AUC
mean_tpr /= n_classes

fpr["macro"] = all_fpr
tpr["macro"] = mean_tpr
roc_auc["macro"] = auc(fpr["macro"], tpr["macro"])

# Plot all ROC curves
plt.figure(1)

plt.grid(True)

plt.plot(fpr["micro"], tpr["micro"],
         label='micro-average ROC curve (area = {0:0.2f})'
               ''.format(roc_auc["micro"]),
         color='deeppink', linestyle=':', linewidth=4)

plt.plot(fpr["macro"], tpr["macro"],
         label='macro-average ROC curve (area = {0:0.2f})'
               ''.format(roc_auc["macro"]),
         color='navy', linestyle=':', linewidth=4)

colors = cycle(['aqua', 'darkorange', 'cornflowerblue','red', 'yellow', 'blue'])
for i, color in zip(range(n_classes), colors):
    plt.plot(fpr[i], tpr[i], color=color, lw=lw,
             label='ROC curve of class {0} (area = {1:0.2f})'
             ''.format(i, roc_auc[i]))

plt.plot([0, 1], [0, 1], 'k--', lw=lw)
plt.xlim([0.0, 1.0])
plt.ylim([0.0, 1.05])
plt.xlabel('False Positive Rate')
plt.ylabel('True Positive Rate')
plt.title('Some extension of Receiver operating characteristic to multi-class')
plt.legend(loc="lower right")
plt.savefig('ROC_SVM_1.tiff')
plt.show()


# Zoom in view of the upper left corner.
plt.figure(2)
plt.xlim(0, 0.2)
plt.ylim(0.8, 1)
plt.plot(fpr["micro"], tpr["micro"],
         label='micro-average ROC curve (area = {0:0.2f})'
```

```
                 ''.format(roc_auc["micro"]),
         color='deeppink', linestyle=':', linewidth=4)

plt.plot(fpr["macro"], tpr["macro"],
         label='macro-average ROC curve (area = {0:0.2f})'
               ''.format(roc_auc["macro"]),
         color='navy', linestyle=':', linewidth=4)

colors = cycle(['aqua', 'darkorange', 'cornflowerblue','red', 'yellow', 'blue'])
for i, color in zip(range(n_classes), colors):
    plt.plot(fpr[i], tpr[i], color=color, lw=lw,
             label='ROC curve of class {0} (area = {1:0.2f})'
             ''.format(i, roc_auc[i]))

plt.plot([0, 1], [0, 1], 'k--', lw=lw)
plt.xlabel('False Positive Rate')
plt.ylabel('True Positive Rate')
plt.title('Some extension of Receiver operating characteristic to multi-class')
plt.legend(loc="lower right")
plt.savefig('ROC_SVM_2.pdf')
plt.show()
```

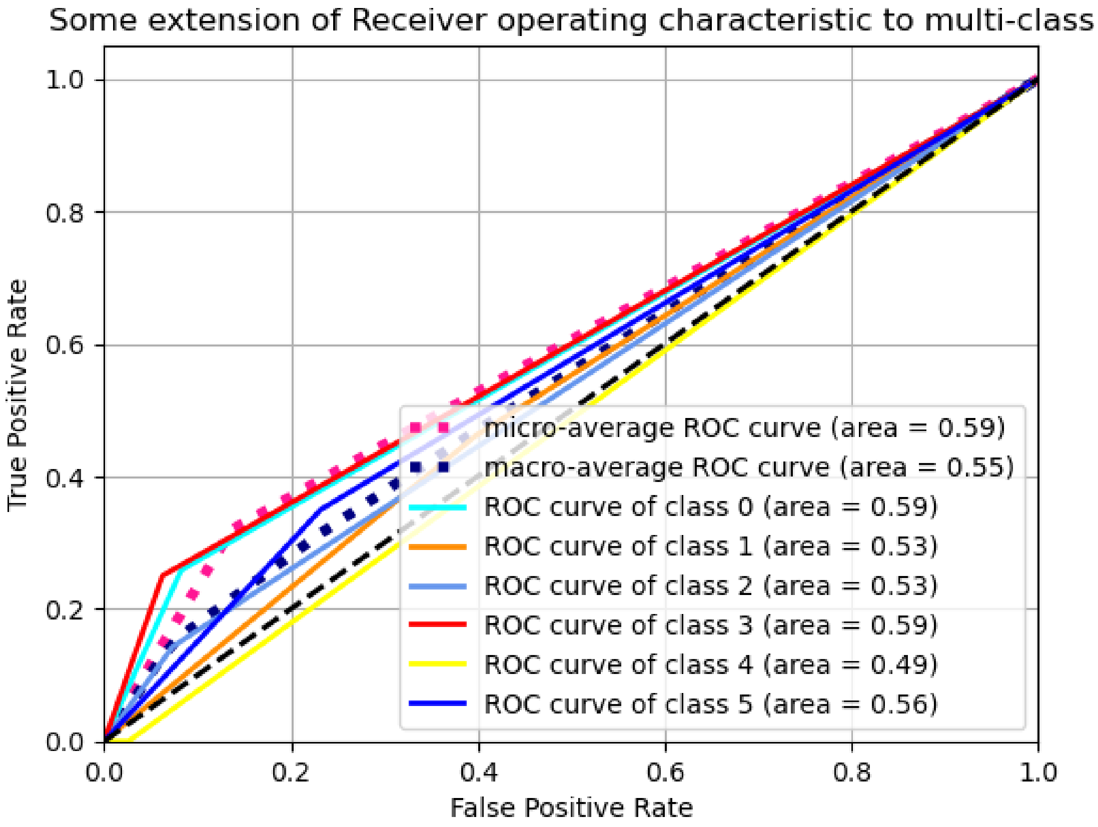

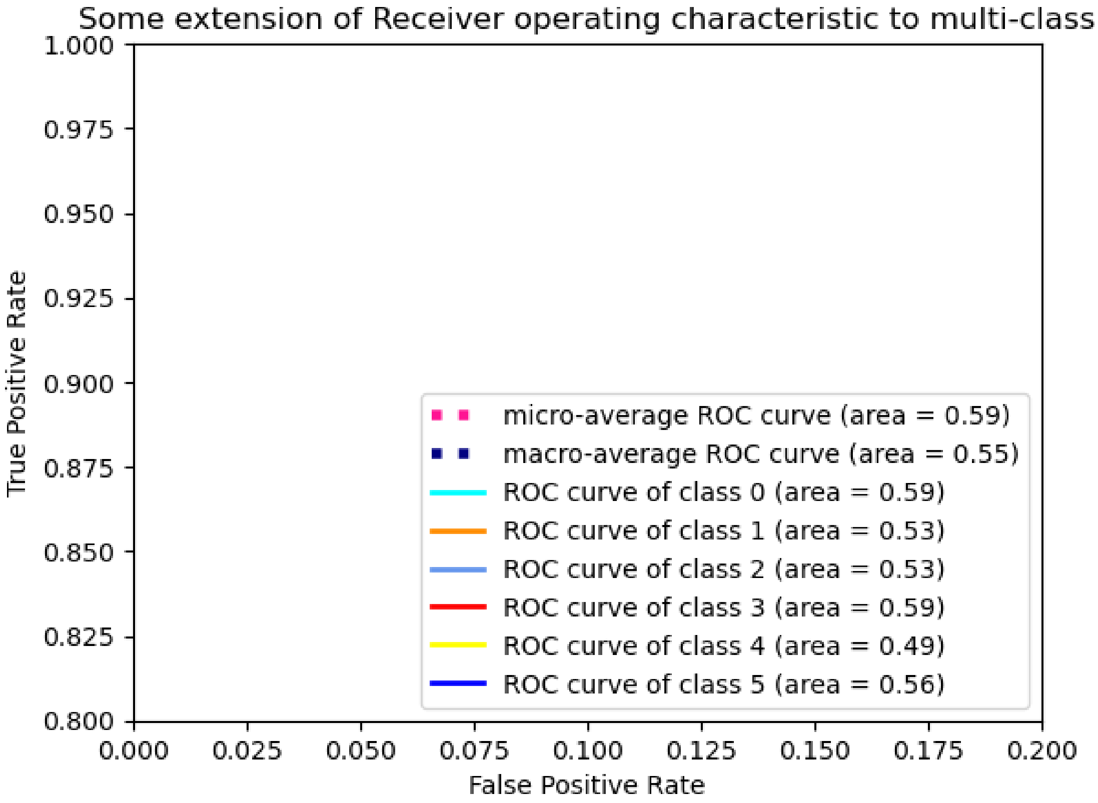
Some extension of Receiver operating characteristic to multi-class
1.000
0.975
0.950
0.925
0.900
0.875
0.850
0.825
0.800
True Positive Rate
0.000
0.025
0.050
0.075
0.100
0.125
0.150
0.175
0.200
False Positive Rate
micro-average ROC curve (area = 0.59)
macro-average ROC curve (area = 0.55)
ROC curve of class 0 (area = 0.59)
ROC curve of class 1 (area = 0.53)
ROC curve of class 2 (area = 0.53)
ROC curve of class 3 (area = 0.59)
ROC curve of class 4 (area = 0.49)
ROC curve of class 5 (area = 0.56)

[ ]:
